# A detailed method for copper doping of synthetic hydroxyapatite thin films via plasma-assisted pulsed laser deposition

**DOI:** 10.1371/journal.pone.0358441

**Published:** 2026-09-25

**Authors:** Leonardo Bohorquez Santiago, Diana Marcela Devia Narváez, Rogelio Ospina Ospina

**Affiliations:** 1 Universidad Tecnológica de Pereira, Pereira-Risaralda, Colombia; 2 Departamento de Física, Facultad de Ciencias Básicas, Universidad Tecnológica de Pereira, Pereira-Risaralda, Colombia; 3 Doctorado en Ciencias, Universidad Tecnológica de Pereira, Pereira-Risaralda, Colombia; 4 Universidad Industrial de Santander, Bucaramanga, Santander, Colombia; Shanghai Jiao Tong University Medical School Affiliated Ruijin Hospital, CHINA

## Abstract

This work provides a detailed and reproducible methodology for the fabrication of copper-doped hydroxyapatite (HAp-Cu) thin films on silicon substrates via Plasma-Assisted Pulsed Laser Deposition (PAPLD). The methodology is centered on the nanosecond pulsed laser ablation of a synthetic hydroxyapatite (HAp) target, physically modified through the incorporation of metallic copper filaments, which serve as a localized dopant source during the deposition process. This configuration enables the controlled co-deposition of hydroxyapatite and copper species, facilitating the formation of uniform thin films with potential bioactive properties. The proposed protocol comprises the following key elements: • **Preparation of Targets:** A comprehensive, stepwise procedure for the synthesis of hydroxyapatite powder and the subsequent fabrication of mechanically robust ceramic targets, including a novel approach for embedding copper filaments to ensure effective doping during laser ablation. • **Deposition Parameters:** Detailed specifications of the PAPLD system, encompassing laser characteristics (wavelength, fluence, repetition rate), target-to-substrate distance, and chamber vacuum conditions. These parameters have been optimized to ensure reproducibility in film growth and compositional consistency. • **Validation Procedure:** A systematic approach to validating the deposition process, focused on confirming the incorporation of copper into the hydroxyapatite matrix and verifying the formation of crystalline HAp-Cu films through targeted characterization techniques, distinct from exhaustive physicochemical or biological evaluations. The methodology described herein establishes a reliable framework for the fabrication of HAp-Cu coatings via PAPLD, offering a reproducible route for future studies requiring precise control over dopant incorporation within hydroxyapatite-based thin films. For further details regarding the physicochemical characterization and biological assessment of the resulting coatings, the reader is referred to a complementary publication by the authors.

## Section 1: Introduction

Hydroxyapatite (Ca_10_(PO_4_)_6_(OH)_2_, HAp) is a calcium phosphate ceramic widely recognized as a cornerstone material in biomedical engineering, owing to its compositional and structural resemblance to the mineral phase of vertebrate bone and teeth. This intrinsic similarity underlies its excellent biocompatibility, bioactivity, and osteoconductivity, making it highly suitable for applications such as bone void fillers, scaffolds for tissue regeneration, and, critically, as interfacial coatings on metallic implants to enhance osseointegration and mitigate the bio-inertness of metallic substrates [1]. However, native HAp exhibits negligible antimicrobial activity, which constitutes a significant limitation given the persistent challenge of implant-associated infections [2–5].

To overcome this drawback, the incorporation of therapeutic metallic ions into the HAp matrix has emerged as a widely explored strategy. Among these, copper (Cu) ions are particularly attractive due to their well-documented broad-spectrum antibacterial efficacy against a range of pathogenic microorganisms [3,6]. Furthermore, copper is recognized for its roles in stimulating angiogenesis and osteogenesis, biological processes essential for enhancing bone regeneration and the long-term success of implant integration [5,8,9].

Pulsed Laser Deposition (PLD) is a versatile and energetic physical vapor deposition technique capable of producing high-purity, adherent, and stoichiometric thin films of complex, multi-elemental materials such as HAp on various substrates [7,10–12]. The process involves the irradiation of a solid target with high-intensity laser pulses, resulting in the ejection of material in the form of a plasma plume, which subsequently condenses onto a substrate. In the present work, the plasma-assisted pulsed laser deposition configuration (PAPLD) affords precise control over the resulting film’s microstructure and properties through the adjustment of laser parameters (e.g., fluence, wavelength, repetition rate) and vacuum chamber conditions (e.g., background gas type and pressure).

Our research group has previously reported the successful synthesis of copper-doped HAp (HAp-Cu) thin films via a bespoke PAPLD approach and has undertaken an extensive characterization of their structural, compositional, morphological, surface, and biological properties [1]. That work primarily focused on the performance and scientific evaluation of the resulting materials. Recognizing the value of a detailed methodological account to ensure reproducibility and foster further advancements in this field, the present article provides a comprehensive and structured protocol for the fabrication of HAp-Cu coatings. This includes: (i) the synthesis of HAp powder and its consolidation into mechanically robust PLD targets; (ii) a practical method for the incorporation of metallic copper filaments onto the target surface to serve as the dopant reservoir; (iii) the specific configuration of the PAPLD system, encompassing both optical and vacuum components; and (iv) the precise deposition parameters required for the successful formation of HAp-Cu thin films on silicon substrates.

This detailed methodological account is intended to facilitate the adoption, replication, and potential refinement of this HAp-Cu deposition strategy within the biomaterials research community. Additional information regarding the subject area, method scope, and key methodological features is summarized in the Specifications Table.

A distinctive feature of the proposed methodology is the use of metallic copper strips directly incorporated onto the hydroxyapatite target surface, enabling in situ dopant incorporation through simultaneous laser ablation of both materials. Unlike conventional approaches based on chemically pre-doped hydroxyapatite powders or pre-fabricated Cu-containing targets, the present strategy provides a flexible and experimentally simple route for controlling copper incorporation during film growth. While our previous publication focused on the physicochemical and biological properties of HAp-Cu coatings, the present work provides the complete experimental protocol required for reproducible fabrication and adaptation of the methodology by other researchers.

**Fig 1:**
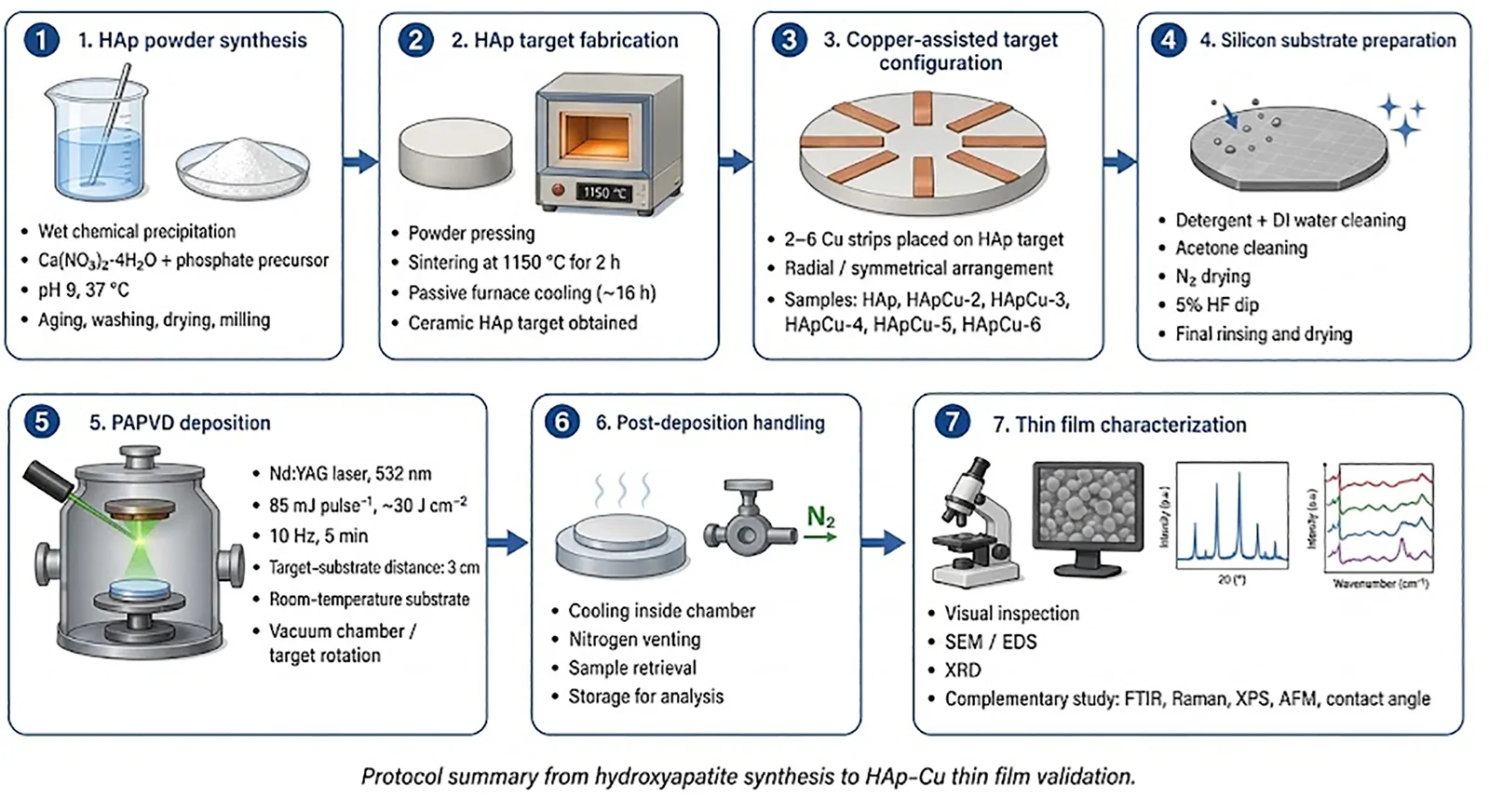
Flowchart summarizing the overall experimental procedure used for the fabrication and validation of copper-doped hydroxyapatite (HAp-Cu) thin films, including HAp synthesis, target fabrication, copper-strip incorporation, silicon substrate cleaning, PAPVD deposition, post-deposition handling, and thin-film characterization.

For clarity, the overall experimental workflow followed in this study is summarized in Fig 1.

## Section 2: Materials and methods

The described peer-reviewed protocol is published on protocols.io: https://doi.org/10.17504/protocols.io.q26g7kjdklwz/v1

### Ethics statements

This research involved no human subjects or animal experiments. All materials and procedures were handled in accordance with standard laboratory safety protocols.

### Materials and target preparation

The quality and composition of the PLD target are critical factors influencing the resulting thin film properties. In this methodology, hydroxyapatite (HAp) targets were fabricated in-house from synthetically prepared HAp powder to ensure precise control over purity and stoichiometry. The preparation process involved the synthesis of HAp powder, followed by consolidation and high-temperature sintering to produce mechanically robust ceramic targets suitable for pulsed laser deposition [12].

### Synthesis of hydroxyapatite powder

- Precursors: Analytical-grade calcium nitrate tetrahydrate (Ca(NO_3_)_2_·4H_2_O, ≥ 99.0%, [CAS No. 13477-34-4, e.g., Sigma-Aldrich]) and diammonium hydrogen phosphate ((NH_4_)_2_HPO_4_, ≥ 98%, [CAS No. 7783-28-0, e.g., Merck]) were employed as calcium and phosphate sources, respectively. Ammonium hydroxide solution (NH_4_OH, 28–30% NH_3_ basis, [e.g., Sigma-Aldrich]) was used for pH adjustment. High-purity deionized water (Milli-Q grade or equivalent, resistivity >18 MΩ·cm) was used throughout all procedures.
- Precipitation Reaction: Hydroxyapatite was synthesized via a wet chemical precipitation route. A 1.0 M aqueous solution of Ca(NO_3_)_2_·4H_2_O and a separate 0.6 M aqueous solution of (NH_4_)_2_HPO_4_ were prepared. The phosphate solution was added dropwise (~5 mL/min using a peristaltic pump or burette) into the calcium solution under vigorous mechanical stirring (400 rpm, overhead stirrer) in a reaction vessel maintained at 37 ± 0.5 °C using a temperature-controlled water bath. The precursor concentrations were selected to provide the stoichiometric Ca/P molar ratio of 1.67 required for hydroxyapatite formation. Throughout the addition, the pH was continuously monitored using a calibrated pH meter and actively maintained at 9.0 ± 0.2 by the controlled addition of NH_4_OH solution. This pH range is critical for favoring the formation of hydroxyapatite while minimizing the precipitation of undesired calcium phosphate phases [12].
- **Aging, Washing, and Filtration:** Upon completion of precursor addition (~2 hours), the resulting suspension was aged for 48 hours at room temperature without stirring to promote crystal growth and enhance stoichiometry. The aged precipitate was then separated from the supernatant by vacuum filtration using Whatman No. 42 ashless filter paper. The collected filter cake was thoroughly washed with multiple volumes of hot (~80 °C) deionized water to remove residual nitrates, ammonium ions, and other soluble by-products. Washing was continued until the electrical conductivity of the filtrate decreased below 5 µS/cm, indicating adequate removal of ionic contaminants.
- **Drying and Milling:** The purified precipitate was dried in a convection oven at 100 °C for 24 hours to eliminate residual moisture. The dried HAp, typically presenting as soft agglomerates, was manually ground to a fine powder using an agate mortar and pestle to achieve a uniform particle size distribution. The resulting powder was then sieved through a 77 µm aperture stainless steel mesh to ensure homogeneity, rendering it suitable for subsequent pressing and sintering processes.

The hydroxyapatite phase obtained using this synthesis route and subsequently deposited as thin films was experimentally verified in our previously published study [1]. X-ray diffraction (XRD) confirmed the characteristic crystalline HAp phase, with the diffraction patterns indexed according to PDF No. 00-009-0432. In addition, Fourier transform infrared spectroscopy (FTIR) showed the characteristic phosphate vibrational bands of HAp at approximately 1024 and 960 cm^-1^ [1].

### Fabrication of HAp ceramic targets

- Pressing: For each target, 5.0 grams of the synthesized and sieved HAp powder were weighed and uniaxially pressed in a 26 mm diameter hardened steel die. A compaction pressure of 200 MPa was applied for 10–15 minutes using a hydraulic press to form mechanically stable green compacts. No organic binders or additives were introduced to preserve the chemical purity of the material.
- Sintering: The green compacts were sintered in a programmable muffle furnace (e.g., Carbolite^TM^ CWF1300) under ambient air to improve consolidation and mechanical integrity suitable for PLD applications. The sintering schedule was as follows:
  1. **Initial heating ramp:** 3 °C/min from room temperature to 700 °C, with a 30-minute dwell at 700 °C to promote the gradual removal of adsorbed species and to minimize the risk of cracking.
  2. **Secondary heating ramp:** 1 °C/min from 700 °C to 1150 °C, followed by a 2-hour dwell at 1150 °C to facilitate densification and grain growth.
  3. **Cooling:** Following the 2 h dwell at 1150 °C, the furnace was powered off, and the targets were allowed to cool naturally to room temperature within the furnace chamber over approximately 16 hours. Since the cooling stage was not actively programmed, the cooling rate was not constant; the overall average cooling rate was approximately 1.2 °C/min. This slow furnace-cooling procedure was employed to minimize thermal stresses and prevent cracking of the sintered ceramic targets.

The resultant HAp targets exhibited dimensions of approximately 24–25 mm in diameter and 3–4 mm in thickness. The sintered targets were white, mechanically robust, and demonstrated suitable density and integrity for subsequent use in the PLD process [12].

### Substrate cleaning protocol

The preparation of pristine substrate surfaces is essential to ensure optimal film adhesion and to achieve the desired physical and chemical properties of the deposited coatings. In this study, single-crystal silicon (100) wafers (n-type or p-type, specify as appropriate) with dimensions of 10 mm × 10 mm × 0.5 mm and a resistivity <0.005 Ω·cm were used as substrates. The following multi-step wet chemical cleaning protocol was rigorously implemented immediately prior to introducing the substrates into the PLD system.

### Cleaning procedure

- **Degreasing:** Substrates were immersed in a 1:1 (v/v) solution of laboratory-grade neutral detergent (e.g., Decon 90 or Liquinox, 1:1 (v/v) in deionized water) and deionized water. The beaker containing the substrates was placed in an ultrasonic bath (40 kHz, 100 W for 10 minutes to remove organic contaminants and particulates.
- **Deionized Water Rinsing:** Following degreasing, substrates were thoroughly rinsed with flowing deionized water and subsequently subjected to three sequential 8-minute ultrasonic cleaning cycles in fresh deionized water to ensure complete removal of detergent residues.
- **Organic Solvent Cleaning:** The substrates were then immersed in analytical-grade acetone (≥99.8%, e.g., Sigma-Aldrich) and sonicated for 10 minutes to eliminate residual organic films.
- **Nitrogen Drying:** After solvent cleaning, substrates were immediately dried using a directed stream of high-purity (99.999%) filtered nitrogen gas.
- **Native Oxide Removal (HF Dip):** To remove the native silicon dioxide (SiO_2_) layer and achieve a hydrogen-terminated or oxide-free silicon surface (depending on the desired surface chemistry), substrates were briefly immersed for 30 seconds in a 5% (v/v) hydrofluoric acid solution prepared from a 49% HF stock solution and deionized water.

[Critical Safety Note:] Hydrofluoric acid is highly hazardous. This step must be conducted in a certified, well-ventilated fume hood specifically approved for HF use. Appropriate personal protective equipment (PPE) includes HF-resistant gloves (e.g., nitrile over neoprene), chemical splash goggles, a face shield, and an HF-impervious apron. Calcium gluconate gel must be readily available as an emergency first-aid measure for HF exposure.

- **Final Deionized Water Rinsing:** Immediately after the HF dip, substrates were thoroughly rinsed with copious amounts of deionized water and subjected to three additional 8-minute ultrasonic cleaning cycles in fresh deionized water to ensure complete removal of HF residues and any reaction by-products.
- **Final Nitrogen Drying:** Cleaned substrates were dried thoroughly using high-purity nitrogen gas. Substrates were either loaded into the load-lock chamber of the PLD system within 30 minutes or stored in a clean, sealed container (e.g., Fluoroware^®^) under a dry nitrogen atmosphere to prevent recontamination and minimize native oxide regrowth.

### Experimental Setup for Pulsed Laser Ablation (PAPLD)

**Fig 2:**
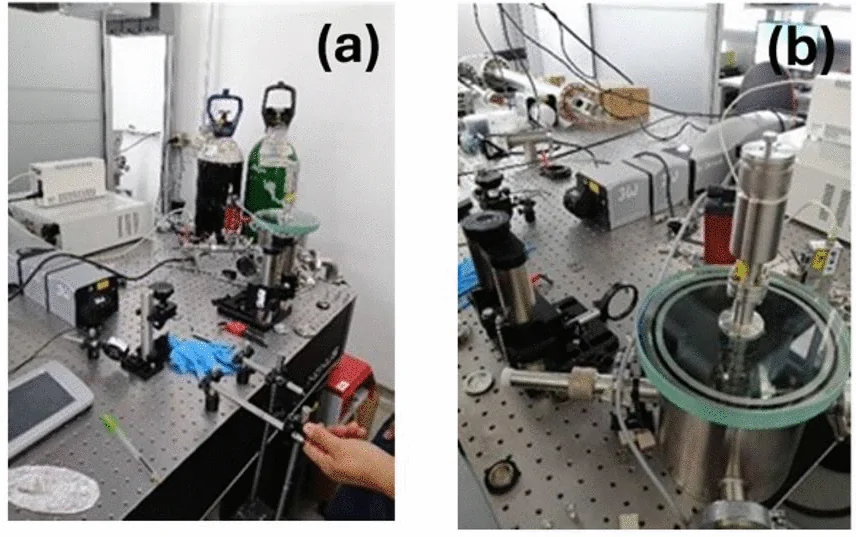
Experimental setup for coating production. (a) Front view of the experimental setup on the vibration-isolating table. (b) Side view of the experimental setup on the vibration-isolating table. (Source: Author).

**Fig 3:**
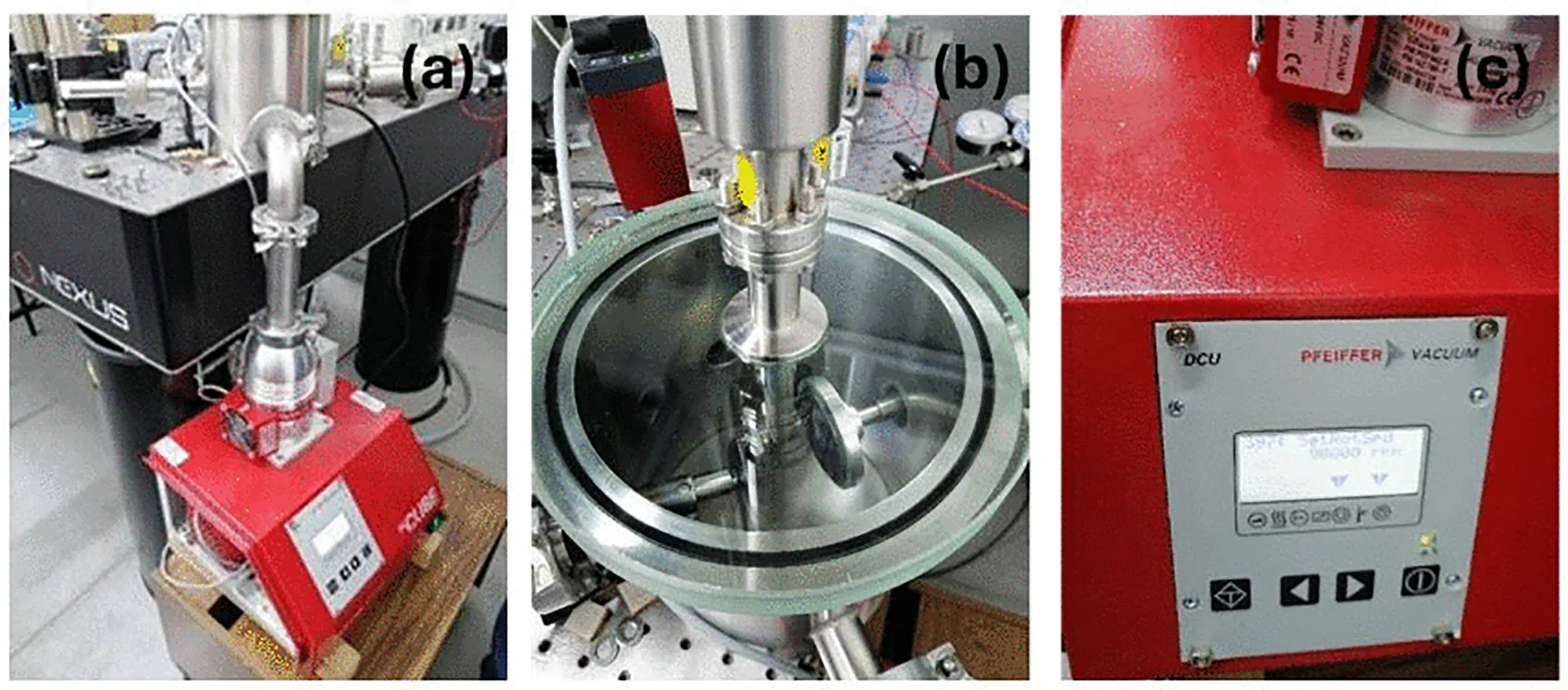
Vacuum equipment for deposition of thin films using pulsed lasers. (a) Assembly of the PFEIFFER VACUUM vacuum equipment. (b) Vacuum chamber with substrate and target holders. (c) Configuration of the vacuum equipment control panel. (Source: Author).

The deposition of HAp-Cu thin films was carried out using a custom-designed Plasma-Assisted Pulsed Laser Deposition (PAPLD) system. A schematic overview of the experimental setup is provided in Figs 2 and 3.

- **Laser Source and Optics:** A Q-switched Nd:YAG laser (Quantel Q-smart 850, or equivalent) operating at its second harmonic (λ = 532 nm) with a pulse duration of 6 ns was employed as the ablation source. The laser beam was directed to the target through a series of high-reflectivity dielectric mirrors (optimized for 532 nm) and focused onto the target surface using a UV-grade fused silica plano-convex lens with a focal length of 300 mm. The focusing optics were positioned outside the vacuum chamber, and the laser beam entered the chamber through an anti-reflection-coated quartz window. The laser spot size on the target surface was adjusted to approximately 0.60 in diameter, providing the desired fluence at the focal point.
- **Vacuum Chamber and Pumping System:** The deposition chamber consisted of a stainless-steel cylindrical vessel (cylindrical chamber of approx. 10 L volume) equipped with multiple ports for pumping, pressure monitoring, laser entry, and in-situ diagnostics (if applicable). High vacuum conditions were achieved using a turbomolecular pump (PFEIFFER VACUUM, HiCube 80 Eco, or equivalent), backed by a two-stage rotary vane pump. The system was capable of reaching a base pressure below 5 × 10^-6^ mbar (equivalent to 5 × 10^-4^ Pa or 3.75 × 10^-6^ Torr). Chamber pressure was continuously monitored using a combination of Pirani and cold cathode gauges.
- **Target Manipulator:** The sintered HAp target, modified with copper filaments as detailed in Section 2, was mounted on a motorized target manipulator enabling simultaneous rotation (set at 60 rpm) and rastering (programmable linear movement across the target radius, e.g., over a 15 mm track) during ablation. This dynamic manipulation ensured uniform target erosion, mitigated localized overheating or drilling effects, and contributed to the generation of a stable and homogeneous plasma plume throughout the deposition process.
- **Substrate Stage:** The cleaned silicon substrates were affixed to the substrate holder using stainless steel clips or thermally conductive silver paste if thermal contact was required; however, deposition here was performed at room temperature. The substrate holder was positioned directly opposite the target at a fixed target-to-substrate distance (d_t-s_) of 3.0 cm. No intentional substrate heating or cooling was applied during deposition; substrates remained at ambient temperature throughout the process.

Silicon substrates were selected as model substrates due to their smooth surface, chemical stability, and widespread use in thin-film deposition studies. Their use facilitates the characterization of deposited coatings while minimizing substrate-related effects on film growth. Although the methodology was developed using silicon substrates, the deposition strategy is expected to be transferable to metallic biomaterials such as titanium and titanium alloys, with appropriate optimization of deposition parameters to account for differences in surface properties, thermal conductivity, and coating adhesion.

- **System Stability:** To ensure mechanical stability and minimize vibrational disturbances that could compromise laser-target alignment or film uniformity, the entire PAPLD apparatus, including the laser system and vacuum chamber, was mounted on a vibration-isolation optical table (NEXUS, or equivalent).

### Doping and deposition process

**Fig 4:**
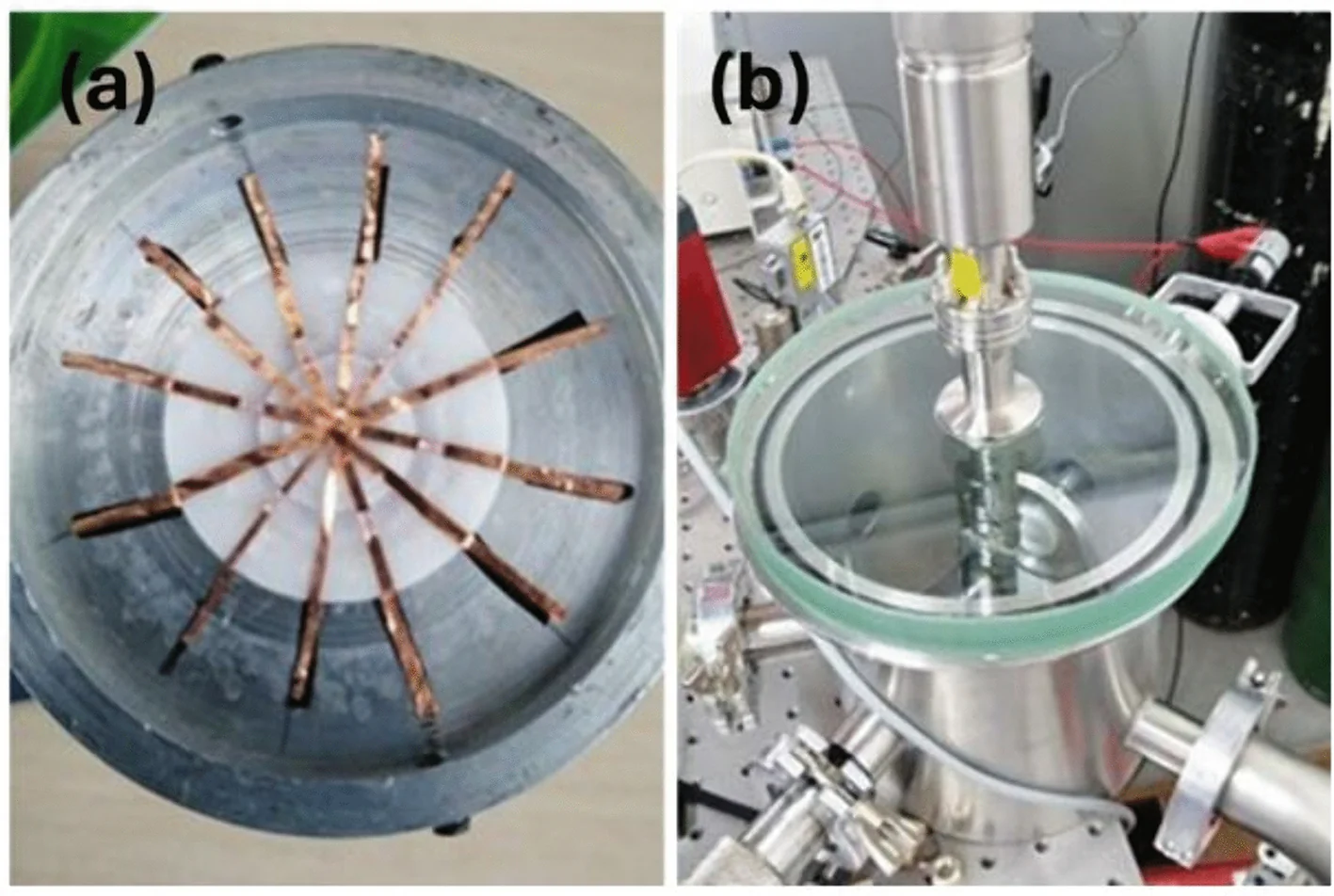
Hydroxyapatite target used in the experimental process. (a) Hydroxyapatite target with thin copper strips. (b) Target located in the vacuum chamber for the experimental process. (Source: Author).

A distinctive feature of this methodology is the in-situ incorporation of copper into the hydroxyapatite (HAp) thin films during the PAPLD process, achieved through the concurrent ablation of the HAp target and metallic copper sources placed directly onto its surface. A schematic representation of this configuration is provided in Fig 4 in the next section.

- **Target Preparation for Doping:** Prior to each deposition run, the surface of the sintered HAp target was prepared for doping. Thin strips of high-purity (99.99%) metallic copper foil (thickness: 0.1 mm; width: 1.0 mm) were precisely cut. A varying number of strips (2, 3, 4, 5, or 6, corresponding to samples labeled HApCu-2 through HApCu-6, respectively) were arranged symmetrically in a radial pattern on the ablating surface of the HAp target, as illustrated in Fig 4a. This symmetric radial configuration was intentionally designed to promote homogeneous copper incorporation throughout the deposited films. In combination with continuous target rotation and rastering during deposition, the arrangement ensured periodic laser interaction with both the hydroxyapatite surface and the copper strips, favoring a stable co-ablation process and reducing the likelihood of localized compositional gradients within the resulting coatings.

Despite the different thermal conductivity, optical absorption, and laser–material interaction characteristics of copper and hydroxyapatite, no significant instability of the ablation process was observed during deposition. The symmetric arrangement of the copper strips, combined with continuous target rotation and rastering, promoted uniform target erosion and stable plasma generation throughout repeated laser irradiation cycles. Furthermore, no evidence of target cracking, copper-strip detachment, or plasma plume fluctuations that could compromise deposition reproducibility was observed under the selected operating conditions.

The deposition target therefore consisted of a sintered hydroxyapatite ceramic modified with high-purity metallic copper strips, allowing the simultaneous ablation of both materials and the in-situ incorporation of copper into the growing film.

The use of metallic copper strips instead of pre-mixed copper-containing hydroxyapatite targets was intentionally adopted to provide greater flexibility in controlling the copper content during deposition. This approach allows the dopant level to be adjusted by varying the number of copper strips without requiring the fabrication of multiple ceramic targets with different compositions. Furthermore, it avoids potential structural modifications of hydroxyapatite associated with the high-temperature sintering of Cu-containing targets and facilitates rapid optimization of deposition conditions.

Additionally, pre-sintered Cu-containing HAp targets may undergo phase transformations or copper oxidation during high-temperature processing, whereas the use of metallic copper strips preserves the original hydroxyapatite target composition and introduces copper directly during the ablation stage.

The copper incorporation level was controlled by varying the number of copper strips placed on the target surface while maintaining all other deposition parameters constant. The symmetric radial arrangement of the copper strips, combined with continuous target rotation during deposition, promoted a reproducible co-ablation process and a homogeneous distribution of copper-containing species within the plasma plume. Copper incorporation into the deposited films was subsequently verified by EDS analysis.

Care was taken to ensure intimate physical contact between the copper strips and the HAp surface. The strips were secured using shallow surface indentations in the ceramic, temporary vacuum-compatible non-outgassing adhesives at the outer, non-ablated edges, or mechanical retention by the target manipulator’s clamping mechanism if applicable. This arrangement ensured that, as the target rotated and rastered during deposition, the laser beam would intermittently ablate both HAp and copper, enabling controlled co-deposition.

Deposition protocol:

The principal PAPVD parameters employed for all deposition experiments are summarized in Table 1.

**Table 1:** Summary of the experimental parameters used for PAPVD deposition of HAp-Cu thin films.

Parameter	Experimental value
Laser source	Q-switched Nd:YAG
Laser wavelength	532 nm
Pulse duration	6 ns
Energy per pulse	85 mJ
Laser spot diameter	~0.60 mm
Irradiated spot area	~0.283 mm^2^
On-target laser fluence	~30 J·cm^-2^
Pulse repetition rate	10 Hz
Target-to-substrate distance	3.0 cm
Substrate	Silicon (100)
Substrate temperature	Room temperature
Target composition	HAp target with 99.99% purity Cu strips
Number of Cu strips	0, 2, 3, 4, 5, or 6
Target rotation speed	60 rpm
Deposition time	5 min
Post-deposition cooling	30 min inside the chamber
Post-deposition thermal treatment	None
Base pressure	<5 × 10^-6^ mbar
Working/deposition pressure	6.3 × 10^-5^ mbar

**Fig 5:**
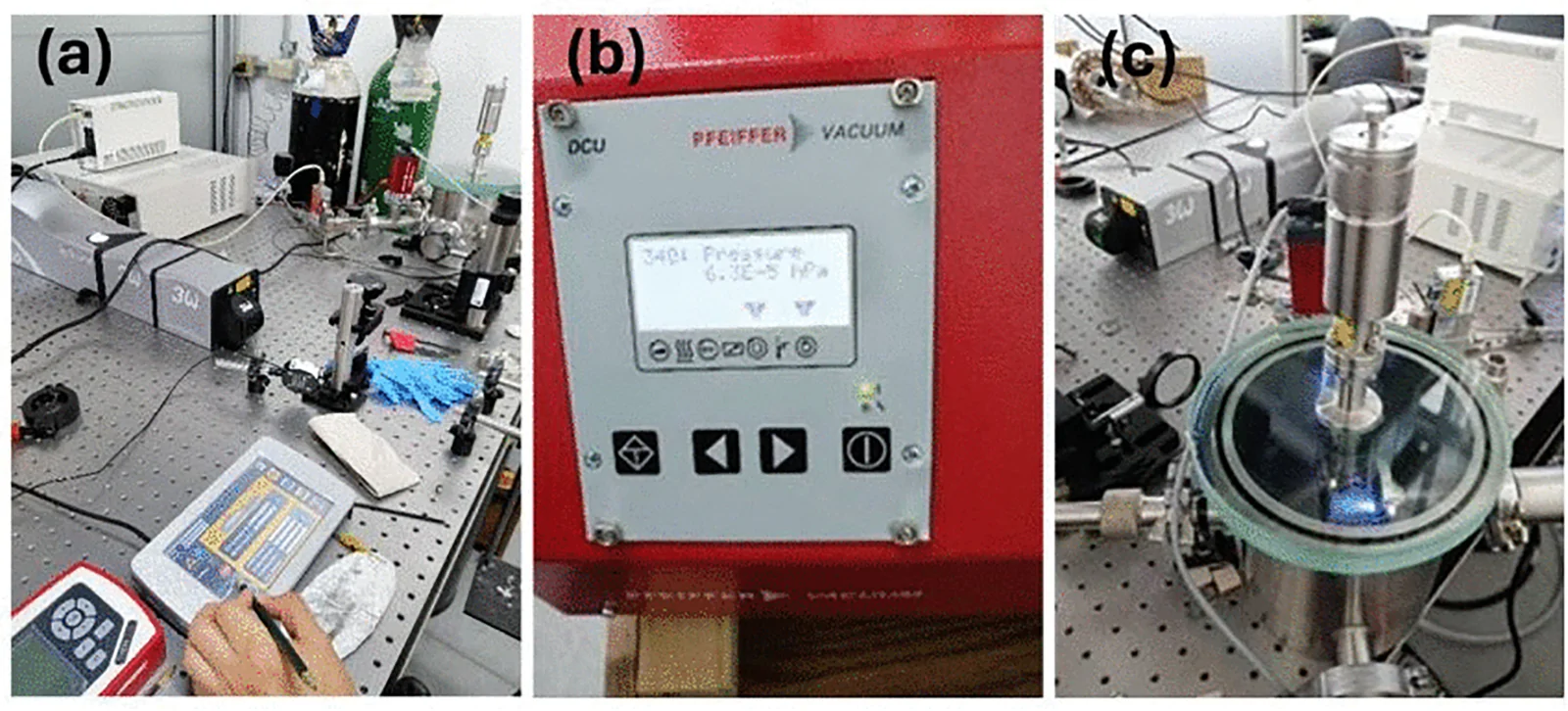
Deposit of copper-doped hydroxyapatite coating by laser ablation technique. (a) Laser configuration according to [10,13]. (b) Pressure reading obtained with the vacuum equipment. (c) Plasma generated in the vacuum chamber for the generation of the hydroxyapatite coating. (Source: Author).

The prepared HAp target and the cleaned silicon substrates were mounted in the deposition chamber (Fig 5b), which was evacuated to a base pressure below 5 × 10^-6^ mbar. A working pressure of 6.3 × 10^-5^ mbar was subsequently established and maintained throughout the deposition process [8,9,14].

Depositions were performed on silicon substrates maintained at room temperature without intentional substrate heating. The target-to-substrate distance was fixed at 3 cm throughout all experiments.

The target fluence was achieved by adjusting the focusing optics to produce a laser spot of approximately 0.60 mm in diameter, corresponding to an irradiated area of approximately 0.283 mm^2^ (2.83 × 10^-3^ cm^2^). Considering an energy per pulse of 85 mJ, this spot size corresponds to an on-target laser fluence of approximately 30 J/cm^2^.

The target holder was driven by an electric motor operating at 6.9 V and 0.01 A, providing a constant rotation speed of 10 rpm throughout the deposition process.

Each deposition was performed for a fixed duration of precisely 5 minutes. During this period, ablated species from both the HAp and copper components formed an energetic plasma plume, which expanded towards and condensed onto the room-temperature silicon substrate, resulting in the formation of the HAp-Cu thin film.

Under these operating conditions, a stable plasma plume was generated throughout the deposition process, enabling the reproducible transfer of both hydroxyapatite and copper species from the target to the substrate surface.

- **Post-Deposition Handling:** Upon completion of the 5-minute deposition, the laser system was deactivated, and the substrates were left to cool to near room temperature within the vacuum chamber. A minimum cooling period of 30 minutes was observed to ensure thermal equilibrium and to minimize potential thermal stress effects on the thin films.

Following cooling, the vacuum chamber was slowly vented to atmospheric pressure using high-purity dry nitrogen gas to prevent atmospheric moisture or contaminants from interacting with the freshly deposited films.

No additional post-deposition thermal treatment or annealing process was applied. Substrates were then retrieved and stored in clean, sealed containers (e.g., Petri dishes or dedicated sample boxes) under ambient laboratory conditions until further characterization.

## Section 3: Results and discussion

The successful formation of copper-doped hydroxyapatite (HAp-Cu) thin films using the described PAPLD protocol was validated through a targeted set of characterization techniques. These analyses were designed to confirm the occurrence of film deposition, the incorporation of copper, the morphological characteristics, and the primary crystalline phase of the films. A comprehensive analysis of the physicochemical and biological properties of the resulting coatings is available in a separate, detailed publication [1]. The present validation focuses solely on verifying the reproducibility and effectiveness of the methodological protocol.

### Visual appearance and film formation

**Fig 6:**
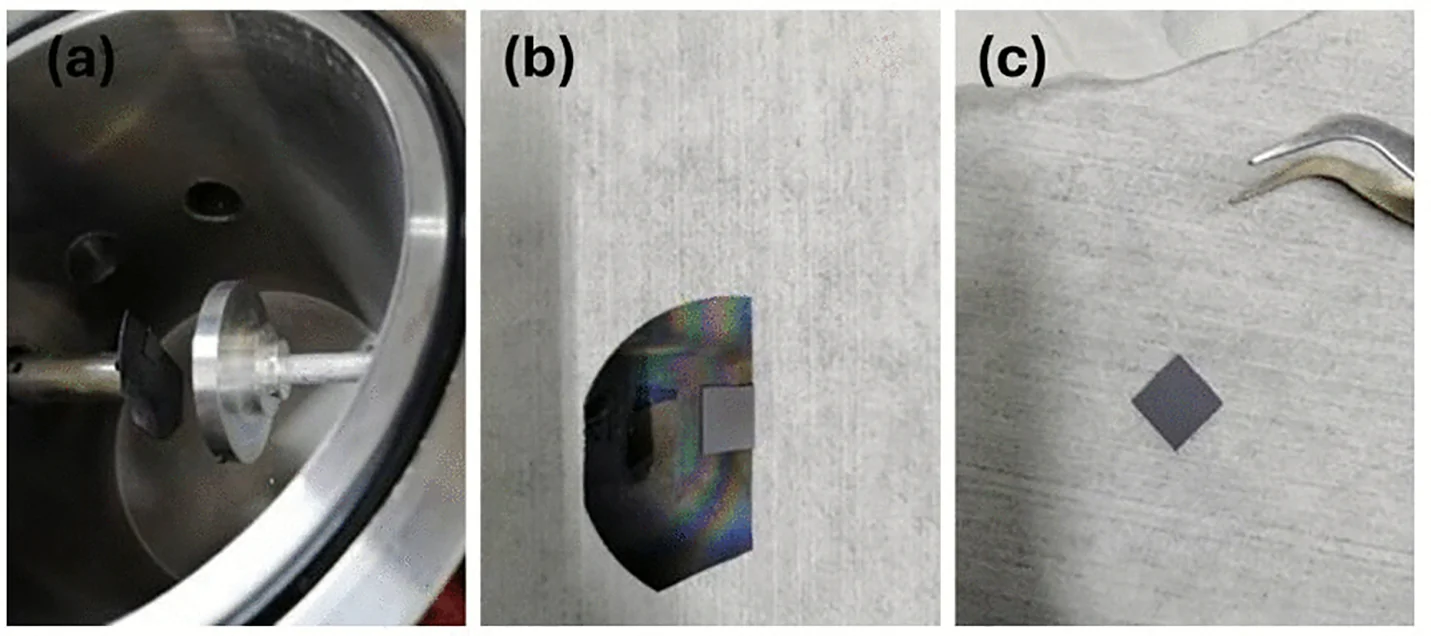
Substrate obtained from the experimental laser ablation process. (a) Vacuum chamber at the end of the experimental process. (b) Silicon substrate coated with hydroxyapatite on the equipment support. (c) Silicon substrate coated with copper-doped hydroxyapatite (Hap-Cu). (Source: Author).

Successful film deposition was initially confirmed through visual inspection of the substrates immediately after the deposition process (Fig 5). The HAp-Cu thin films exhibited a distinct visual appearance when compared to the bare silicon substrates, typically displaying interference colors indicative of thin, transparent, or semi-transparent coatings. These interference effects provided a qualitative indication of successful material transfer and uniform film growth across the substrate surface (Fig 6).

### Confirmation of Copper Incorporation (EDS)

**Fig 7:**
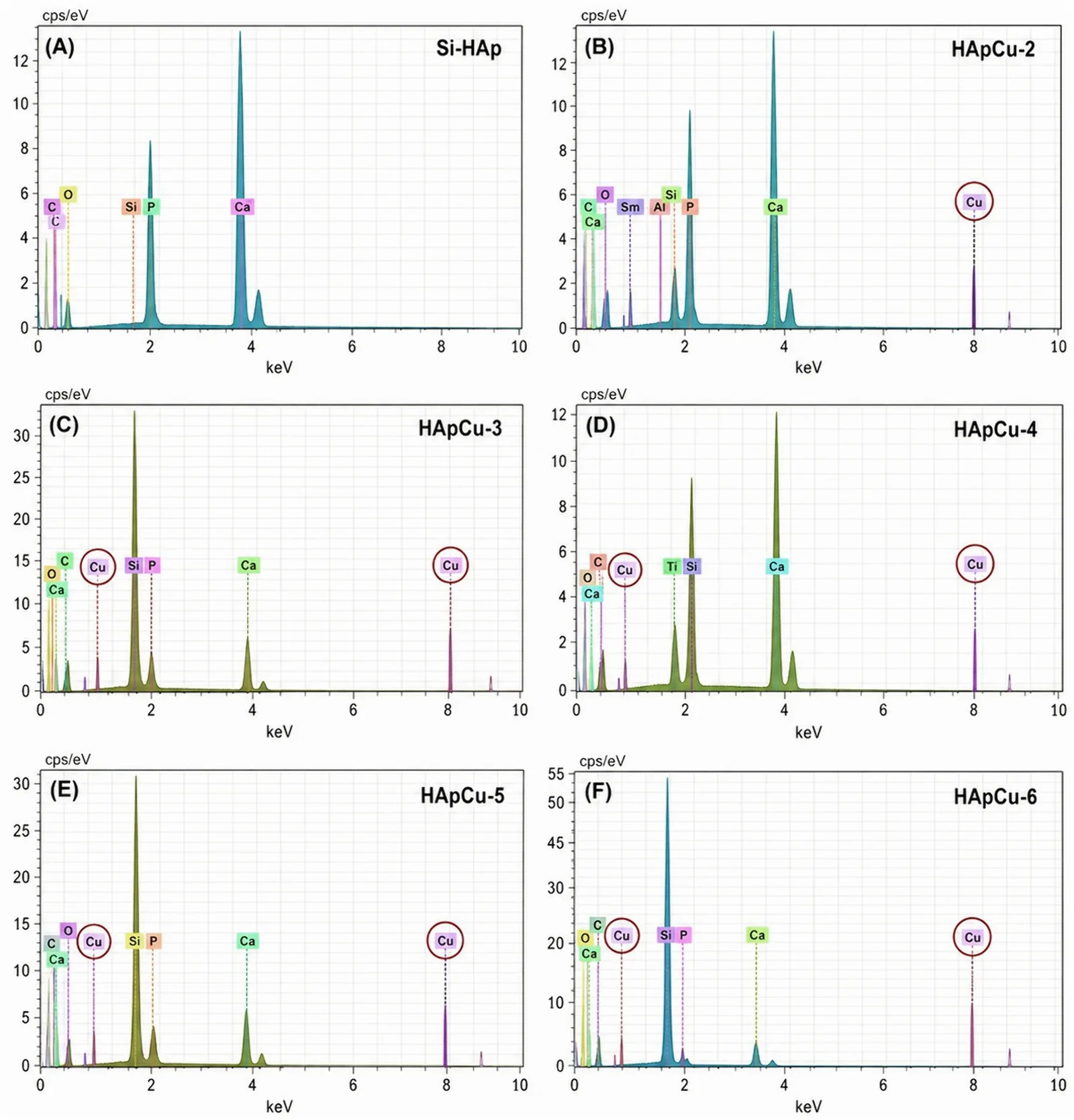
EDS spectrum obtained from a HAp-Cu thin film deposited by PAPLD. (A) Si sample with Cu-free HAp coating. (B) Si sample with HAp coating with two Cu wires. (C) Si sample with HAp coating with three Cu wires. (D) Si sample with HAp coating with four Cu wires. (E) Si sample with HAp coating with five Cu wires. (F) Si sample with HAp coating with six Cu wires. (Source: Author).

The presence of copper within the deposited HAp-Cu films was confirmed through Energy Dispersive X-ray Spectroscopy (EDS) analysis performed in conjunction with Scanning Electron Microscopy (SEM). Fig 7 presents the EDS spectra obtained from the Cu-free HAp coating and the HAp-Cu coatings prepared using two to six Cu strips.

The spectrum reveals the characteristic X-ray emission peaks corresponding to calcium (Ca Lα, Kα), phosphorus (P Kα), oxygen (O Kα), and silicon (Si Kα, originating from the substrate). Crucially, the detection of copper peaks (Cu Lα, Kα) provides clear evidence of the successful incorporation of copper into the HAp matrix via the co-ablation approach described.

Detailed quantitative compositional analyses and elemental mapping confirming the uniform distribution of copper across the film surface are available in our complementary publication [1].

### Film Morphology and Structure (SEM)

**Fig 8:**
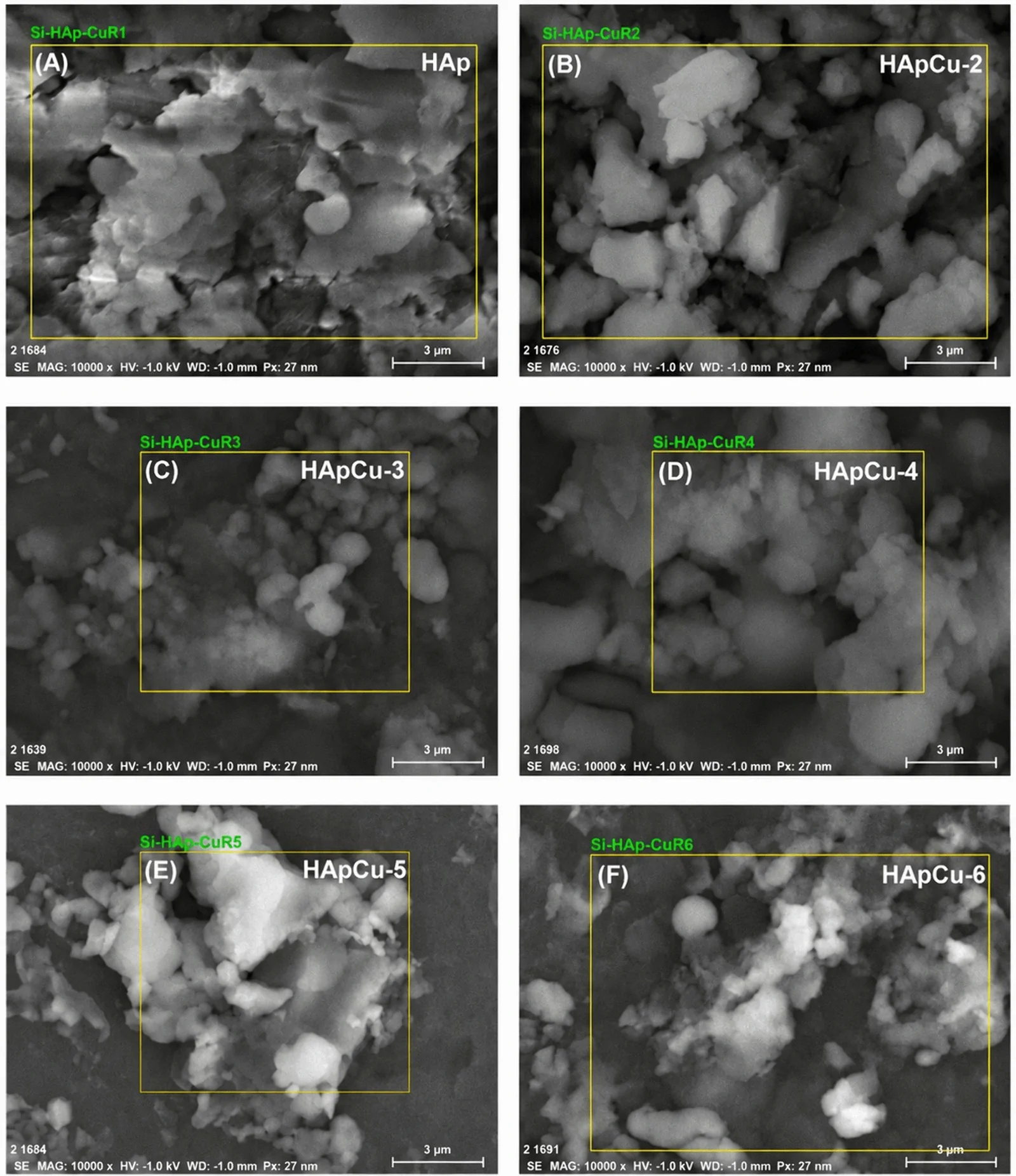
Surface SEM micrograph of the deposited coatings. (A) Si sample with Cu-free HAp coating. (B) Si sample with HAp coating with two Cu strips. (C) Si sample with HAp coating with three Cu strips. (D) Si sample with HAp coating with four Cu strips. (E) Si sample with HAp coating with five Cu strips. (F) Si sample with HAp coating with six Cu strips.

Scanning Electron Microscopy (SEM) was employed to evaluate the surface morphology and overall structural features of the deposited HAp-Cu films. Fig 8 shows surface SEM micrographs of the Cu-free HAp coating and the HAp-Cu coatings prepared using two to six Cu strips [11].

The image reveals **a continuous and relatively dense film composed of nano- to sub-micron-sized spherical or quasi-spherical particulates agglomerated into a uniform layer,** a morphology commonly observed in films produced via pulsed laser deposition (PLD) at room temperature. This morphology confirms the formation of a coherent, continuous coating rather than isolated particulates or discontinuous island structures.

Additional surface morphological and compositional characterization of these coatings is reported in our complementary publication [1]. However, the quantitative data obtained via SEM regarding the elemental composition of the surface are presented in the following table (Table 2).

**Table 2:** Composición elemental semicuantitativa de películas de HAp, HApCu-2, HApCu-3, HApCu-4, HApCu-5 y HApCu-6 [1].

	Elemento	Peso %	Ca/P
HAp	Ca P Cu	44.58 19.68 0	2.26
HApCu-2	Ca P Cu	39.14 16.37 0.23	2.39
HApCu-3	Ca P Cu	19.97 10.55 0.47	1.89
HApCu-4	Ca P Cu	39.06 15.82 0.52	2.47
HApCu-5	Ca P Cu	20.92 11.50 0.77	1.82
HApCu-6	Ca P Cu	12.31 7.31 0.62	1.68

### Crystalline Phase Confirmation (XRD)

**Fig 9:**
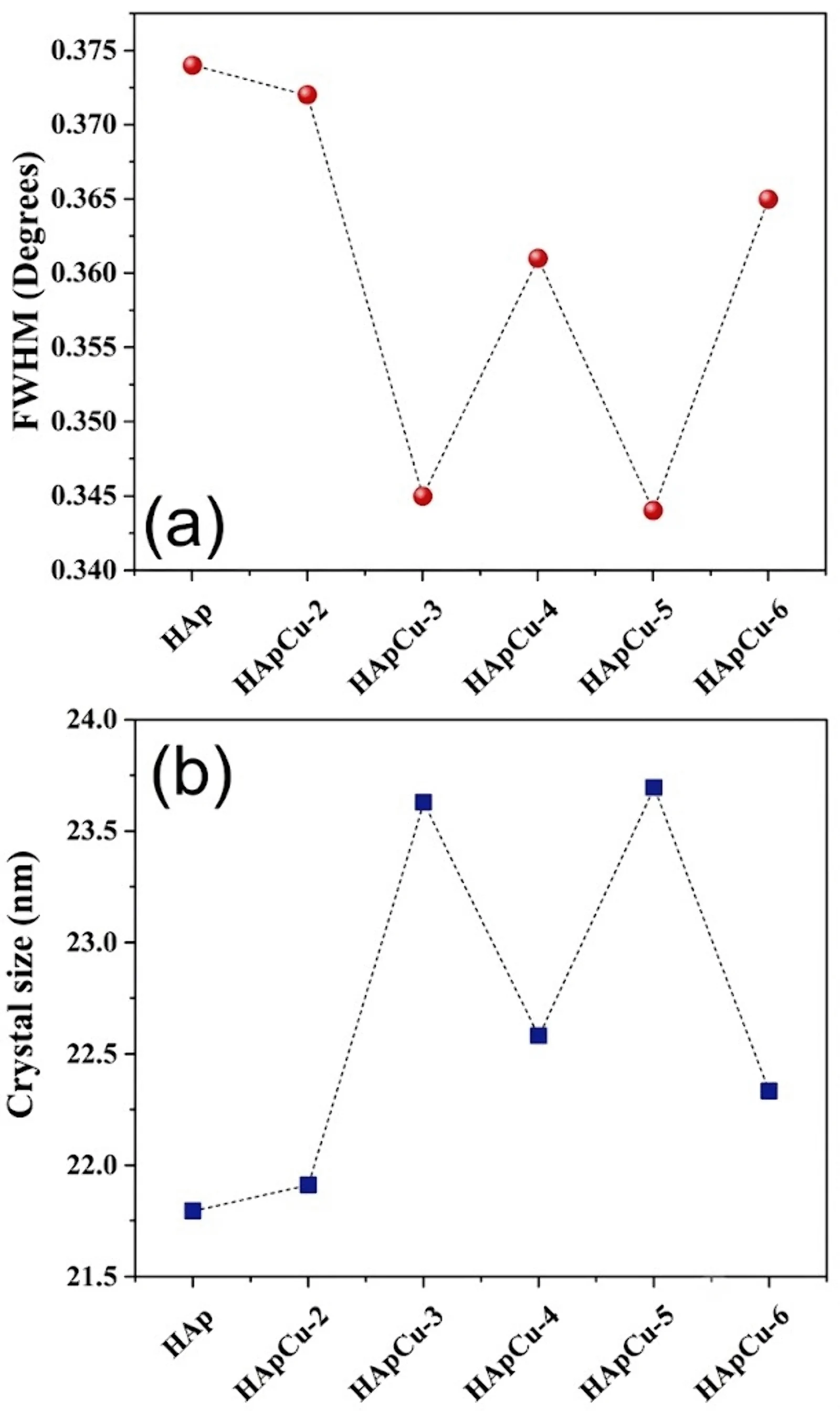
XRD-derived structural parameters of HAp and HAp-Cu coatings. (a) Full width at half maximum (FWHM) values obtained from the (002) reflection for HAp, HApCu-2, HApCu-3, HApCu-4, HApCu-5, and HApCu-6 coatings. (b) Crystallite size calculated from the corresponding XRD analysis using the Scherrer approach. Complete XRD patterns, phase identification, and Miller indices (hkl) are reported in the complementary publication [1].

X-ray diffraction (XRD) analysis was used to evaluate the crystalline characteristics of the HAp and HAp-Cu coatings. Fig 9 summarizes the full width at half maximum (FWHM) values and the crystallite sizes derived from the XRD analysis for the HAp, HApCu-2, HApCu-3, HApCu-4, HApCu-5, and HApCu-6 coatings. Crystallite sizes were determined from the (002) reflection using the Scherrer approach, as described in our complementary study [1]. The FWHM values varied from approximately 0.344° to 0.374°, while the corresponding crystallite sizes ranged from approximately 21.8 to 23.7 nm. As expected, an inverse relationship between FWHM and crystallite size was observed: coatings exhibiting narrower diffraction peaks showed comparatively larger crystallite sizes. The HAp and HApCu-2 coatings exhibited the smallest crystallite sizes, whereas HApCu-3 and HApCu-5 showed the largest values. Overall, the changes in FWHM and crystallite size with increasing number of Cu strips were non-monotonic, indicating that Cu incorporation did not produce a simple linear evolution of the crystalline domain size.

The complete XRD patterns corresponding to these same HAp and HAp-Cu coatings were previously reported and discussed in detail in our complementary publication [1]. In that study, the diffraction patterns of HAp, HApCu-2, HApCu-3, HApCu-4, HApCu-5, and HApCu-6 were indexed according to the standard hydroxyapatite reference PDF No. 00-09-0432, confirming the preservation of the characteristic crystalline HAp phase after deposition. No additional crystalline phases attributable to metallic copper, copper oxides, or secondary calcium phosphate phases were identified within the detection limits of XRD [1,12].

Furthermore, the analysis of the (002) reflection reported in [1] showed no significant displacement relative to the standard hydroxyapatite reference. This observation indicates that Cu incorporation under the investigated PAPVD conditions did not produce a measurable modification of the HAp crystalline lattice within the resolution of the XRD measurements. Accordingly, the absence of distinct Cu-containing crystalline phases suggests that the incorporated copper may predominantly occur in a non-crystalline or segregated state rather than forming a detectable independent crystalline phase. This interpretation is consistent with the complementary structural and compositional analyses previously reported for these coatings [1].

The preservation of the characteristic hydroxyapatite crystalline structure, together with the relatively small variations in FWHM and crystallite size, supports the structural stability of HAp under the PAPVD conditions employed. Although Cu incorporation influences the crystallographic characteristics of the coatings, the available XRD evidence does not indicate the formation of detectable secondary crystalline phases or a substantial transformation of the hydroxyapatite structure.

Because the complete diffraction patterns and their corresponding Miller indices (hkl) have already been published in the complementary study [1] and cannot be reproduced in the present manuscript, Fig 9 is restricted to the XRD-derived structural parameters. Readers are therefore referred to Ref [1]. for the complete diffraction profiles, Miller-index assignments, phase identification, and detailed crystallographic analysis.

## Limitations

While the described PAPLD methodology has proven effective for the fabrication of HAp-Cu thin films at the laboratory scale, several limitations inherent to the current approach should be acknowledged:

- **Precise Dopant Control and Uniformity:** Achieving highly precise stoichiometric control of copper at very low concentrations (e.g., sub-ppm levels), as well as ensuring absolute atomic-level homogeneity across the film, remains challenging with the present target configuration employing discrete copper strips. Although the presence of copper at the macro- and microscale is confirmed, nanoscale distribution uniformity may exhibit variability [7,10,13],.
- **Film Adhesion Quantification:** The primary focus of this work was on the fabrication methodology. Quantitative evaluation of film adhesion strength (e.g., scratch testing, pull-off testing, or nanoindentation-based adhesion measurements) was not performed. However, no visible delamination, peeling, or cracking was observed during sample handling and characterization, suggesting adequate film adherence for the purposes of this study. Future work should include quantitative adhesion testing, particularly for biomedical coating applications. Adhesion performance may vary depending on substrate type and surface condition. For more demanding applications, additional optimization strategies—such as the use of adhesion-promoting interlayers or substrate heating—may be necessary [7],.
- The influence of laser fluence, repetition rate, and chamber pressure was not systematically investigated in the present work. These parameters are known to affect plasma plume dynamics, deposition rate, and film composition, and therefore represent important variables for future optimization studies.
- **Scalability and Throughput:** The current PAPLD setup is optimized for small-area, laboratory-scale deposition (e.g., 1 cm² substrates). Scaling this process to larger substrate areas or higher-throughput production suitable for industrial applications would require significant engineering developments, including the design of larger targets or pre-doped composite targets with homogeneously distributed copper [9],. For biomedical implant applications, additional studies addressing coating adhesion, mechanical durability, long-term stability, corrosion behavior, and regulatory requirements would be necessary before clinical or industrial implementation.
- **Influence of Substrate Temperature:** All depositions were performed on room-temperature substrates. While this simplifies processing, it may limit the achievable film crystallinity or density compared to depositions performed at elevated temperatures. The specific effects of substrate temperature on copper incorporation efficiency and resulting film properties using this doping strategy remain to be systematically investigated.
- **Target Degradation:** Extended use of targets modified with metallic strips may lead to non-uniform erosion patterns or shifts in the relative ablation rates of HAp and Cu over time. Such effects could influence film composition in prolonged deposition runs or between targets if preparation consistency is not rigorously maintained.
- **Extension to Other Dopants:** The methodology described herein was demonstrated using copper as the dopant source. However, the same PAPLD strategy could potentially be adapted to other metallic dopants, such as Ag, Zn, or Mg, although additional optimization of deposition parameters would be required to account for differences in laser–material interaction and plasma generation characteristics.
- **Film Thickness Quantification:** Film thickness was not directly measured in the present study. Cross-sectional SEM or profilometric measurements were not performed during the original experimental campaign, and the original coated specimens are no longer available for retrospective thickness determination. Consequently, no numerical film-thickness value is reported. Future applications of this protocol should include direct thickness measurements, preferably by cross-sectional SEM or profilometry, to establish the deposition rate and further improve quantitative reproducibility.

## Section 4: Conclusions

A detailed and reproducible methodology for the fabrication of copper-doped hydroxyapatite (HAp-Cu) thin films by Plasma-Assisted Pulsed Laser Deposition was successfully established and experimentally validated. The proposed approach combines the synthesis of hydroxyapatite powder, the fabrication of mechanically robust ceramic targets, and an in situ copper incorporation strategy based on the controlled placement of metallic copper strips on the target surface, enabling simultaneous ablation of hydroxyapatite and copper during film growth.

The methodology demonstrated stable operation under the selected deposition conditions, including a Nd:YAG laser wavelength of 532 nm, a fluence of 30 J·cm^-2^, a repetition rate of 10 Hz, a chamber pressure of 6.3 × 10^-5^ mbar, and room-temperature deposition on silicon substrates. The symmetric radial arrangement of copper strips, combined with target rotation and rastering, provided a practical approach for promoting reproducible copper incorporation while maintaining process stability throughout repeated deposition cycles.

Method validation through visual inspection, SEM, EDS, and XRD analyses confirmed successful thin-film formation, effective copper incorporation, and preservation of the characteristic hydroxyapatite crystalline phase. EDS analysis verified the presence of copper within the deposited coatings, while XRD results revealed no detectable secondary crystalline phases associated with copper oxides or alternative calcium phosphate compounds, indicating that the incorporation strategy preserved the structural characteristics of hydroxyapatite within the detection limits of the technique.

A key contribution of the present work is the implementation of a flexible doping strategy that avoids the need for pre-synthesized Cu-containing hydroxyapatite powders or pre-doped ceramic targets. The copper incorporation level can be adjusted through simple modifications of the target configuration, providing a practical platform for systematic studies of dopant incorporation during PAPLD processing.

Overall, the methodology presented herein constitutes a robust and transferable experimental framework for the fabrication of Cu-doped hydroxyapatite coatings. Furthermore, the approach may be adapted to other metallic dopants and multifunctional bioactive systems, thereby expanding the possibilities for the development of advanced thin-film coatings for biomedical and surface engineering applications.

### Specifications table

**Table:** 

Subject area	Materials Science
More specific subject area	Hydroxyapatite doping; Pulsed Laser Deposition; Thin Film Synthesis; Plasma-Assisted Pulsed Laser Deposition; Bioactive coatings; Surface modification.
Name of your method	A Detailed Method for Copper Doping of Synthetic Hydroxyapatite (HAp) Thin Films via Plasma-Assisted Pulsed Laser Deposition (PAPLD)
Name and reference of original method	The specific adaptation of PAPVD for HAp-Cu doping detailed herein, along with its scientific outcomes, is first described in: Bohorquez-Santiago, L., et al. (2024), J. Am. Ceram. Soc., e20195 [1].

## Supporting information

S1 File Detailed step-by-step protocol for copper doping of synthetic hydroxyapatite thin films by plasma-assisted pulsed laser deposition (PAPVD). (PDF)

S2 File Experimental procedure, setup, and equipment specifications for copper doping of synthetic hydroxyapatite thin films by plasma-assisted pulsed laser deposition (PAPVD). (PDF)

S3 File Data PLOS. (XLSX)

S4 File Grafical Astract. (TIF)
