# Supplementary material for: A detailed method for copper doping of synthetic hydroxyapatite thin films via plasma-assisted pulsed laser deposition
Source: PLoS One. 2026 Sep 25;21(9):e0358441. doi: 10.1371/journal.pone.0358441 (PMC13614653; doi:10.1371/journal.pone.0358441)
Supplement: Supplementary file 1 [file pone.0358441.s001.pdf]

Mar 09, 2026

Detailed step-by-step protocol for copper doping of synthetic hydroxyapatite thin films by plasma-assisted pulsed laser deposition (PAPVD)

DOI
https://dx.doi.org/10.17504/protocols.io.q26g7kjdklwz/v1

LEONARDO BOHORQUEZ SANTIAGO[1]
[1]UNIVERSIDAD TECNOLOGICA DE PEREIRA

LEONARDO BOHORQUEZ SANTIAGO
UNIVERSIDAD TECNOLÓGICA DE PEREIRA

Create & collaborate more with a free account

Edit and publish protocols, collaborate in communities, share insights through comments, and track progress with run records.

Create free account

OPEN ACCESS

DOI: https://dx.doi.org/10.17504/protocols.io.q26g7kjdklwz/v1

Protocol Citation: LEONARDO BOHORQUEZ SANTIAGO 2026. Detailed step-by-step protocol for copper doping of synthetic hydroxyapatite thin films by plasma-assisted pulsed laser deposition (PAPVD). **protocols.io** https://dx.doi.org/10.17504/protocols.io.q26g7kjdklwz/v1

License: This is an open access protocol distributed under the terms of the **Creative Commons Attribution License**, which permits unrestricted use, distribution, and reproduction in any medium, provided the original author and source are credited

Protocol status: Working
We use this protocol and it's working

Created: March 07, 2026

Last Modified: March 09, 2026

Protocol Integer ID: 273331

Keywords: synthetic hydroxyapatite thin films by plasma, sintered hydroxyapatite, synthetic hydroxyapatite, doped hydroxyapatite, pulsed laser deposition, laser deposition, fabrication of copper, sintering of dense hap target, synthesis of hap powder, step protocol for copper doping, copper doping, thin films on si, deposition parameter, hap powder

Funders Acknowledgements:
Universidad Tecnologica de Pereira

Abstract

This protocol described the fabrication of copper-doped hydroxyapatite (HAp-Cu) thin films on Si (100) substrates using a plasma-assisted pulsed laser deposition (PAPVD) system. Copper was incorporated in situ through intermittent ablation of metallic copper strips placed on the surface of a sintered hydroxyapatite (HAp) target. The protocol included: (i) synthesis of HAp powder; (ii) compaction and sintering of dense HAp targets; (iii) substrate cleaning; (iv) modification of the target with copper strips; (v) deposition parameters and sequence; and (vi) post-deposition handling and verification points.

Image Attribution

Figure 1. Representative image of vacuum system, laser configuration, and radial Cu-strip arrangement.
Figure 2. Representative image of vacuum system, laser configuration, and radial Cu-strip arrangement.
Figure 3. Representative image of vacuum system, laser configuration, and radial Cu-strip arrangement.
Figure 4. Representative image of vacuum system, laser configuration, and radial Cu-strip arrangement.
Figure 5. Representative image of vacuum system, laser configuration, and radial Cu-strip arrangement.

Materials

Reagent / Material Specification
- Calcium nitrate tetrahydrate $Ca(NO_3)_2 \cdot 4H_2O$, ≥99% (CAS 13477-34-4)
- Diammonium hydrogen phosphate $(NH_4)_2HPO_4$, ≥98% (CAS 7783-28-0)
- Ammonium hydroxide NH_4OH, 28–30% NH_3
- Deionized water Resistivity ≥18 MΩ·cm
- Neutral detergent Decon 90 or equivalent (1:1 v/v with DI water)
- Acetone ≥99.8%
- Hydrofluoric acid (HF) 5% v/v solution
- Nitrogen gas 99.999% purity
- Copper foil 99.99%, ~0.1 mm thickness, ~1 mm width
- Ashless filter paper Whatman No. 42 or equivalent
- Stainless steel sieve 77 µm mesh

Equipment Specification
- Mechanical or propeller stirrer Vigorous stirring ~400 rpm.
- Thermostatic bath or heating blanket Temperature control at 37 ± 0.5 °C.
- Calibrated pH meter Continuous pH monitoring; target pH 9.0 ± 0.2.
- Peristaltic pump or burette Controlled addition ~5 mL/min.
- Vacuum filtration system Büchner funnel + vacuum source.
- Drying oven 100 °C for 24 h.
- Agate mortar and pestle Powder homogenization.
- Hydraulic press + 26 mm die Uniaxial pressing at 200 MPa for 10-15 min
- Programmable muffle furnace Up to 1150 °C with controlled ramps.
- Ultrasonic bath 40 kHz, ~100 W (or equivalent).
- PAPVD/PLD chamber Base pressure 3c5 × 10^-6 mbar; target rotation and scanning (rastering).
- Nd:YAG laser 532 nm (2nd harmonic), pulses ~6 ns; 10 Hz; 85 mJ per pulse.
- Optics High reflectivity mirrors; f = 300 mm lens; quartz window with anti-reflective coating.
- Vacuum pumps and gauges Turbomolecular pump with backup; Pirani gauges + cold cathode.
- Energy meter/beam profiler Recommended for verifying fluence and spot size.

Safety warnings

- All procedures involving hydrofluoric acid (HF) were performed inside an HF-certified fume hood using compatible personal protective equipment (double gloves, face shield, safety goggles, and chemical-resistant apron). Calcium gluconate gel was kept readily available for emergency use, and institutional emergency procedures were followed.
- The Nd:YAG pulsed laser (532 nm) was operated under Class 4 laser safety controls (interlocks, beam enclosure whenever possible, and certified protective eyewear for 532 nm). Only trained personnel handled the laser system.
- Furnace operation up to 1150 °C was conducted using thermal gloves and appropriate burn-prevention measures.
- Vacuum and high-voltage operations were carried out following standard vacuum safety practices (view-port protection and controlled venting) and equipment-specific electrical safety regulations.
- Waste solutions containing nitrates/ammonium, acetone, and HF residues were collected separately in designated containers. Incompatible waste streams were not mixed.

Before start

- Confirm the availability and proper functioning of the PAPVD chamber, pumps, pressure gauges, and laser safety interlocks.
- Clean all glassware and reaction vessels with deionized (DI) water. Avoid contamination by phosphates from detergents, especially in glassware intended for synthesis.
- Calibrate the pH meter using two-point calibration and check the temperature probe, if applicable.
- Prepare the work plan for handling hydrofluoric acid (HF): certified fume hood, PPE, spill kit, and calcium gluconate gel available.
- Provide clean, labeled containers for substrates and deposited samples.

Reference times: HAp synthesis (2 h addition + 48 h aging + 1–2 h washing/filtration + 24 h drying); target fabrication (pressing: 15 min; sintering: 20 h, including cooling); substrate cleaning (1 h); deposition run (1 h, including pumping and cooling)

6.7 Representative Experimental Images

1

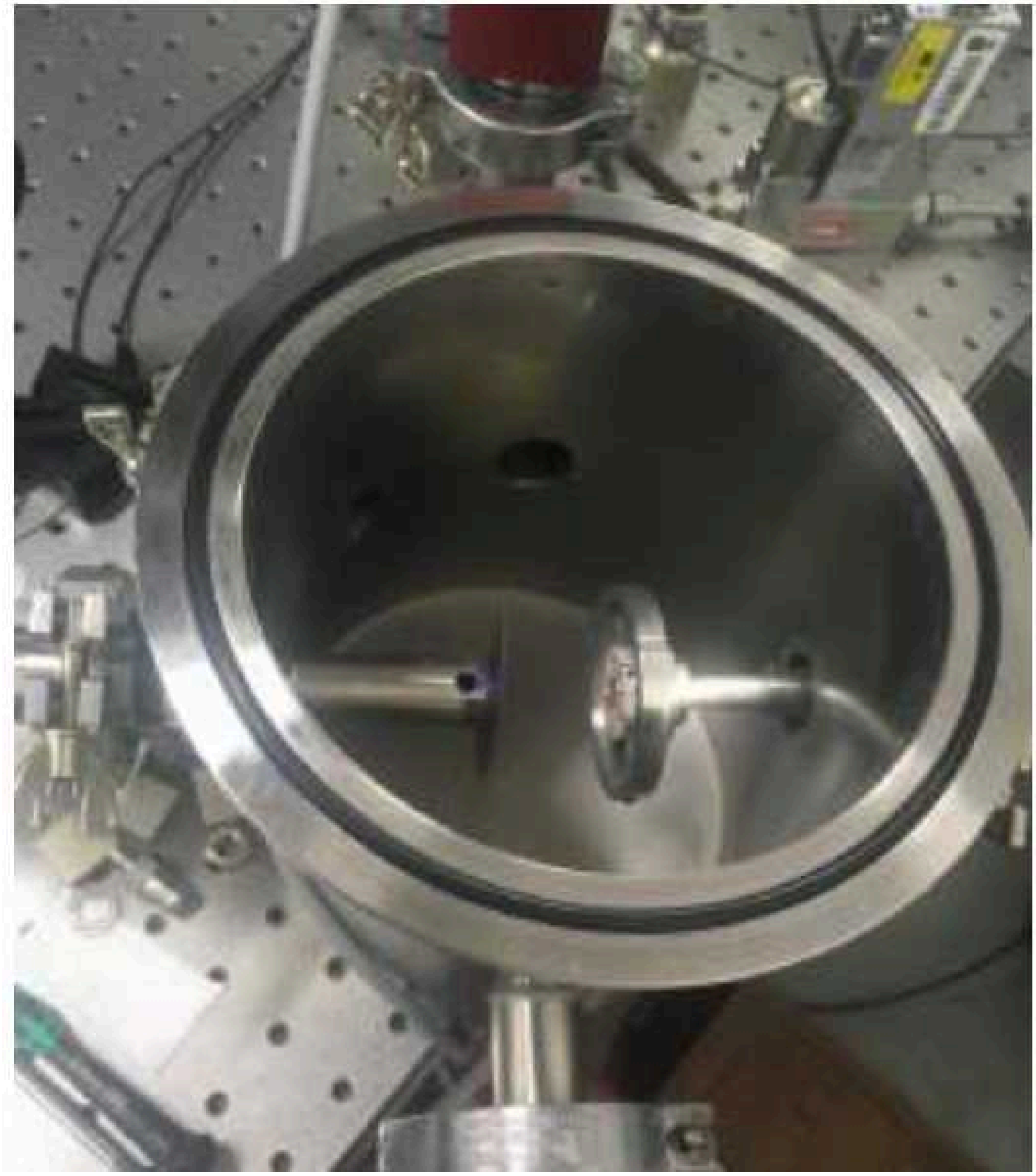

Figure 4. Representative image of vacuum system, laser configuration, and radial Cu-strip arrangement.

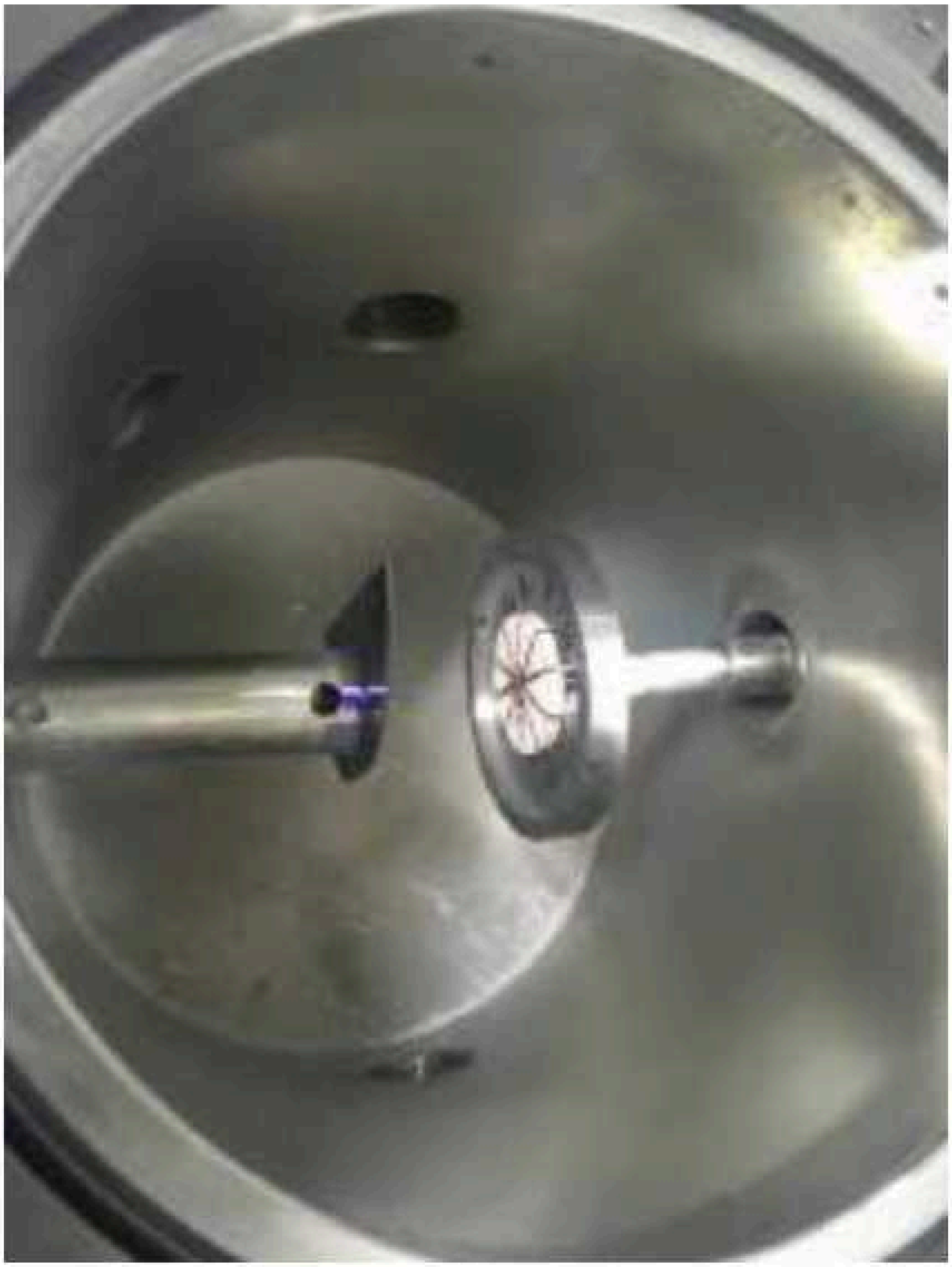

Figure 5. Representative image of vacuum system, laser configuration, and radial Cu-strip arrangement.

6.8 Post-deposition handling

2 Cool the substrates inside the chamber for at least 30 min.

3 Slowly vent the chamber to atmospheric pressure using dry nitrogen to limit moisture adsorption on the fresh films.

4 Remove the samples with clean tweezers and store them in sealed containers (Petri dishes or sample boxes) until characterization.

7. Expected results and verification points

5 A visual inspection was performed for interference colors or visible coating on the bare Si, indicating film formation. Additionally, surface and quantization techniques were used in this case, energy dispersive X-ray spectroscopy (EDS) for the detection of Ca, P, O, and Cu peaks (Si from the substrate) and scanning electron microscopy (SEM) on the continuous coating with nano to submicrometric particles typical of PLD at room temperature.
Finally, a structural analysis with XRD to identify hydroxyapatite peaks without prominent crystalline peaks of Cu/CuO (Cu may be amorphous or below the detection limit).

8. Troubleshooting (common problems)

6 Problem: Target cracks during sintering
Probable cause: Thermal stress; rapid ramps; inhomogeneous green compact
Action/mitigation: Ensure uniform pressing; follow slow ramps (3 °C/min up to 700 °C and 1 °C/min up to 1150 °C); allow to cool with the furnace closed.

7 Problem: No film observed / very low deposition rate
Probable cause: Laser misalignment; low fluence; shutter closed; target out of focus
Action/mitigation: Realign the beam; confirm energy on target; measure spot size; check lens position and window cleanliness.

8 Problem: Unstable pen / excess particles
Probable cause: Localized perforation; insufficient rastering; contaminated target surface
Action/mitigation: Increase rastering coverage; verify rotation at 10 rpm; gently polish/clean the surface; reduce energy per pulse if necessary.

9 Problem: Copper is not detected by EDS.
Probable cause: The strips are not ablated (poor contact/geometry); few strips; short run
Action/mitigation: Improve contact; reposition so that the beam intermittently intersects Cu during rotation; increase the number of strips within the study design; extend the deposition time consistently.

10 Problem: Low adhesion/detachment
Probable cause: Substrate contamination; rust regrowth; handling
Action/mitigation: Minimize the time between HF bath and loading; do not touch the active surface; consider in situ cleaning with plasma/ions if available.

11 Problem: Pressure peaks or surges
Probable cause: Leaks; adhesive outgassing; contaminated chamber
Action/mitigation: Check for leaks; bake/clean the chamber; avoid adhesives that are not vacuum compatible; ensure adequate pumping and throttling.
