# Supplementary material for: A detailed method for copper doping of synthetic hydroxyapatite thin films via plasma-assisted pulsed laser deposition
Source: PLoS One. 2026 Sep 25;21(9):e0358441. doi: 10.1371/journal.pone.0358441 (PMC13614653; doi:10.1371/journal.pone.0358441)
Supplement: Supplementary file 2 [file pone.0358441.s002.pdf]

S2 File. Detailed step-by-step protocol for copper doping of synthetic hydroxyapatite thin films by plasma-assisted pulsed laser deposition (PAPVD)

1. Scope

This protocol described the fabrication of copper-doped hydroxyapatite (HAp-Cu) thin films on Si (100) substrates using a plasma-assisted pulsed laser deposition (PAPVD) system. Copper was incorporated in situ through intermittent ablation of metallic copper strips placed on the surface of a sintered hydroxyapatite (HAp) target. The protocol included: (i) synthesis of HAp powder; (ii) compaction and sintering of dense HAp targets; (iii) substrate cleaning; (iv) modification of the target with copper strips;
(v) deposition parameters and sequence; and (vi) post-deposition handling and verification points.

2. Safety and Compliance Notes

- All procedures involving hydrofluoric acid (HF) were performed inside an HF-certified fume hood using compatible personal protective equipment (double gloves, face shield, safety goggles, and chemical-resistant apron). Calcium gluconate gel was kept readily available for emergency use, and institutional emergency procedures were followed.
- The Nd:YAG pulsed laser (532 nm) was operated under Class 4 laser safety controls (interlocks, beam enclosure whenever possible, and certified protective eyewear for 532 nm). Only trained personnel handled the laser system.
- Furnace operation up to 1150 °C was conducted using thermal gloves and appropriate burn-prevention measures.
- Vacuum and high-voltage operations were carried out following standard vacuum safety practices (view-port protection and controlled venting) and equipment-specific electrical safety regulations.
- Waste solutions containing nitrates/ammonium, acetone, and HF residues were collected separately in designated containers. Incompatible waste streams were not mixed.

3. Reagents and Consumables

Reagent / Material	Specification
Calcium nitrate tetrahydrate	Ca(NO3)2·4H2O, ≥99% (CAS 13477-34-4)
Diammonium hydrogen phosphate	(NH4)2HPO4, ≥98% (CAS 7783-28-0)
Ammonium hydroxide	NH4OH, 28–30% NH3
Deionized water	Resistivity >18 MΩ·cm
Neutral detergent	Decon 90 or equivalent (1:1 v/v with DI water)
Acetone	≥99.8%
Hydrofluoric acid (HF)	5% v/v solution
Nitrogen gas	99.999% purity
Copper foil	99.99%, ~0.1 mm thickness, ~1 mm width
Ashless filter paper	Whatman No. 42 or equivalent
Stainless steel sieve	77 µm mesh

4. Equipment

Equipment	Minimum capacity
Mechanical or propeller stirrer	Vigorous stirring ~400 rpm..
Thermostatic bath or heating blanket	Temperature control at 37 ± 0.5 °C.
Calibrated pH meter	Continuous pH monitoring; target pH 9.0 ± 0.2.
Peristaltic pump or burette	Controlled addition ~5 mL/min.
Vacuum filtration system	Büchner funnel + vacuum source.
Drying oven	100 °C for 24 h.
Agate mortar and pestle	Powder homogenization.
Hydraulic press + 26 mm die	Uniaxial pressing at 200 MPa for 10-15 min
Programmable muffle furnace	Up to 1150 °C with controlled ramps.
Ultrasonic bath	40 kHz, ~100 W (or equivalent).
PAPVD/PLD chamber	Base pressure <5 x 10^-6 mbar; target rotation and scanning (rastering).
Nd:YAG laser	532 nm (2nd harmonic), pulses ~6 ns; 10 Hz; 85 mJ per pulse.
Optics	High reflectivity mirrors; f = 300 mm lens; quartz window with anti-reflective coating.
Vacuum pumps and gauges	Turbomolecular pump with backup; Pirani gauges + cold cathode.
Energy meter/beam profiler	Recommended for verifying fluence and spot size.

5. Antes de comenzar

- Confirm the availability and proper functioning of the PAPVD chamber, pumps, pressure gauges, and laser safety interlocks.
- Clean all glassware and reaction vessels with deionized (DI) water. Avoid contamination by phosphates from detergents, especially in glassware intended for synthesis.
- Calibrate the pH meter using two-point calibration and check the temperature probe, if applicable.
- Prepare the work plan for handling hydrofluoric acid (HF): certified fume hood, PPE, spill kit, and calcium gluconate gel available.
- Provide clean, labeled containers for substrates and deposited samples.

Reference times: HAp synthesis (2 h addition + 48 h aging + 1–2 h washing/filtration + 24 h drying); target fabrication (pressing: 15 min; sintering: 20 h, including cooling); substrate cleaning (1 h); deposition run (1 h, including pumping and cooling)

6. Procedure

6.1 Synthesis of hydroxyapatite (HAp) powder

1. Prepare a 1.0 M aqueous solution of $Ca(NO_3)_2 \cdot 4H_2O$ and, separately, a 0.6 M aqueous solution of $(NH_4)_2HPO_4$, using high-purity deionized (DI) water. Adjust the volumes to maintain an overall Ca/P molar ratio of 1.67.
2. Place the calcium solution in a reactor inside a thermostatic bath at 37 ± 0.5 °C and start vigorous stirring (~400 rpm).
3. Add the phosphate solution dropwise to the calcium solution at a flow rate of approximately ~5 mL/min, while continuously monitoring the pH.

4. Maintain the pH at 9.0 ± 0.2 by controlled addition of NH_4OH throughout the phosphate addition

stage.

5. Continue the addition until all of the phosphate solution has been incorporated (typically ~2 h).
6. Stop stirring and allow the suspension to age for 48 h at room temperature to promote crystal growth.
7. Separate the solid by vacuum filtration using ash-free filter paper.
8. Repeatedly wash the filter cake with hot DI water (~80 °C) until the filtrate has a conductivity < 5 µS/cm to ensure the removal of soluble species (nitrates/ammonium).
9. Dry the washed solid at 100 °C for 24 h.
10. Grind and sieve the powder.
11. Grind the dry agglomerates in an agate mortar and sieve the powder through a 77 µm mesh to homogenize the particle size.

6.2 Manufacture of dense HAp ceramic blanks

12. Weigh the powder. Weigh 5.0 g of sieved HAp powder per blank.
13. Press the green compact. Load the powder into a 26 mm steel die and perform uniaxial pressing at 200 MPa for 10-15 min. Do not add binders if high chemical purity is required.
14. Program the sintering cycle. In a programmable muffle furnace in ambient air: (i) heat at 3 °C/min to 700 °C and hold for 30 min; (ii) heat at 1 °C/min to 1150 °C and hold for 2 h; (iii) turn off and cool naturally to room temperature with the furnace closed (~16 h).
15. Inspect the blank. Verify that it is free of cracks, mechanically robust, and ~24-25 mm in diameter and 3-4 mm thick. Store in a clean container until use.

6.3 Cleaning the silicon substrate (Si(100), 10 mm x 10 mm)

16. Immerse the substrates in a 1:1 (v/v) solution of neutral detergent: DI water and sonicate for 10 min (40 kHz).
17. Rinse thoroughly with DI water.
18. Sonicate in fresh DI water for 8 min; repeat until three cycles are complete to remove detergent residues.
19. Sonicate in acetone for 10 min to remove organic films.
20. Dry immediately with high-purity nitrogen.
21. Immerse in 5% (v/v) HF for 30 s to remove native SiO2. Perform under strict HF safety controls.
22. Rinse immediately with copious amounts of DI water and then perform three additional 8-min sonication cycles in DI water.
23. Dry with nitrogen and load the substrate into the deposition system within the next 30 min.

6.4 Modification of the target for copper doping

24. Cut high-purity Cu foil into strips (typically 0.1 mm thick, 1.0 mm wide). Prepare between 2 and 6 strips depending on the doping level (e.g., HApCu-2 to HApCu-6).
25. The strips were placed symmetrically in a radial pattern on the ablation surface of the HAp ceramic target. The angular separation between the strips was defined as 360°/N (where N is the number of strips) to ensure uniform radial symmetry.

26. Secure using small surface indentations in the ceramic and/or mechanical fastening; if adhesive is required, use only a vacuum-compatible, low-outgassing adhesive applied only to the outer (non-ablated) edges.
27. Mount the modified target on the motorized manipulator. Verify that rotation and rastering do not displace the strips.

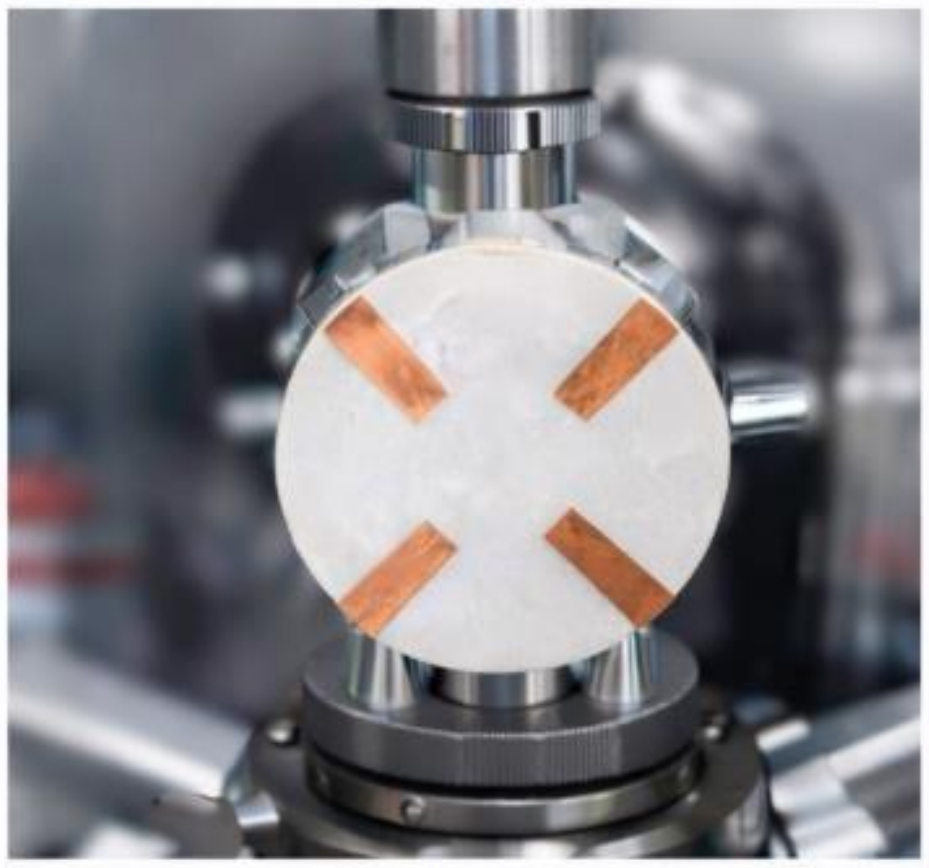

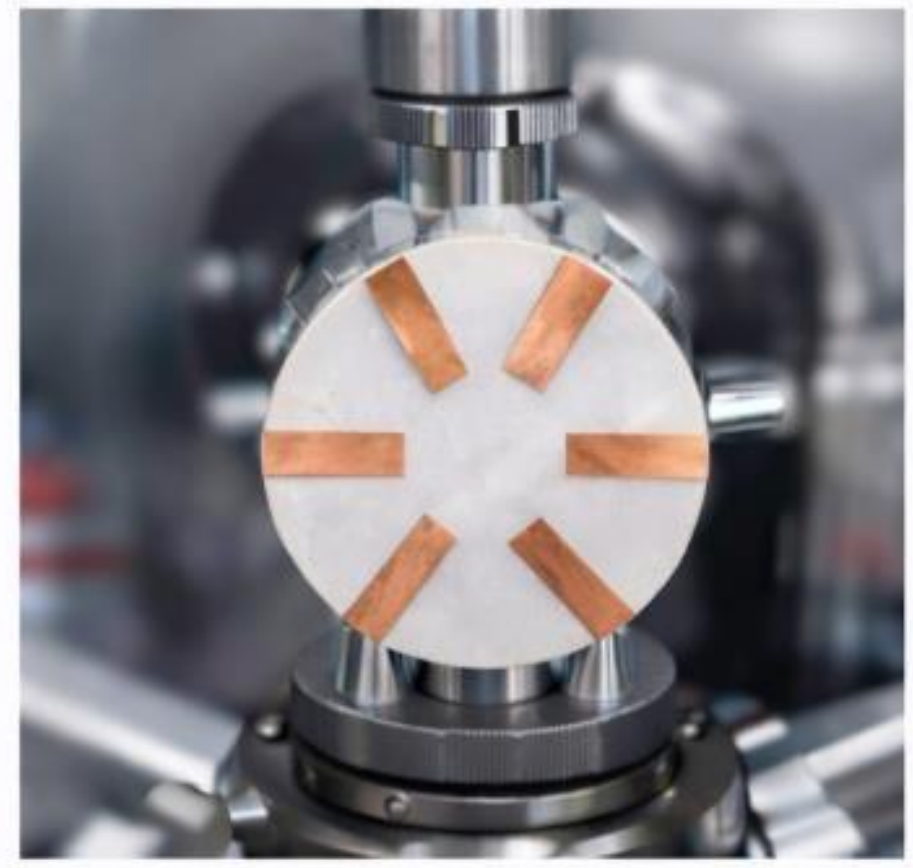

6.5 PAPVD deposition run (HAp-Cu thin films)

28. Mount the clean Si substrates on the sample holder (clips or conductive paste if necessary). Set the target-substrate distance to 5.0 cm. Deposition is performed at room temperature (without intentional heating).
29. Evacuate the chamber to a base pressure below 5 x 10-6 mbar. Check the stability of the pressure gauge readings.
30. Working pressure. Establish and maintain a pressure of 6.3 x 10-5 mbar during deposition (by throttling the pump and/or controlled gas admission, depending on the system). Record the method used.
31. Align the 532 nm Nd:YAG beam using mirrors and a 300 mm focal length lens toward the target through the quartz window. Adjust to achieve a spot diameter of ~0.6-0.7 mm on the target.
32. Set the repetition rate to 10 Hz and the energy per pulse to 85 mJ. Verify a fluence on the target of ~30 J/cm2 using the measured spot size.
33. Start rotating the target at 10 rpm and activate rastering over the surface (trajectory ~15 mm) to avoid localized perforation and stabilize the plume.
34. Fire the laser for 5.0 min while maintaining pressure and movement. Observe the stability of the plume through the viewing window and record any instability.
35. Turn off the laser and then stop the target movement when the plume ceases.
36. Samples were labeled as HAp, HApCu-2, HApCu-3, HApCu-4, HApCu-5, and HApCu-6.

6.6 . Vacuum and Energy Monitoring

37. Base pressure was monitored using a Pfeiffer Vacuum system and recorded prior to ablation.

38. Laser pulse energy stability was monitored using an energy meter. Mean energy, minimum/maximum values, and standard deviation were recorded for each deposition.
39. Flashlamp and Q-switch repetition rates were configured at 10 Hz. Synchronization delay was adjusted according to system optimization parameters.

6.7 Representative Experimental Images

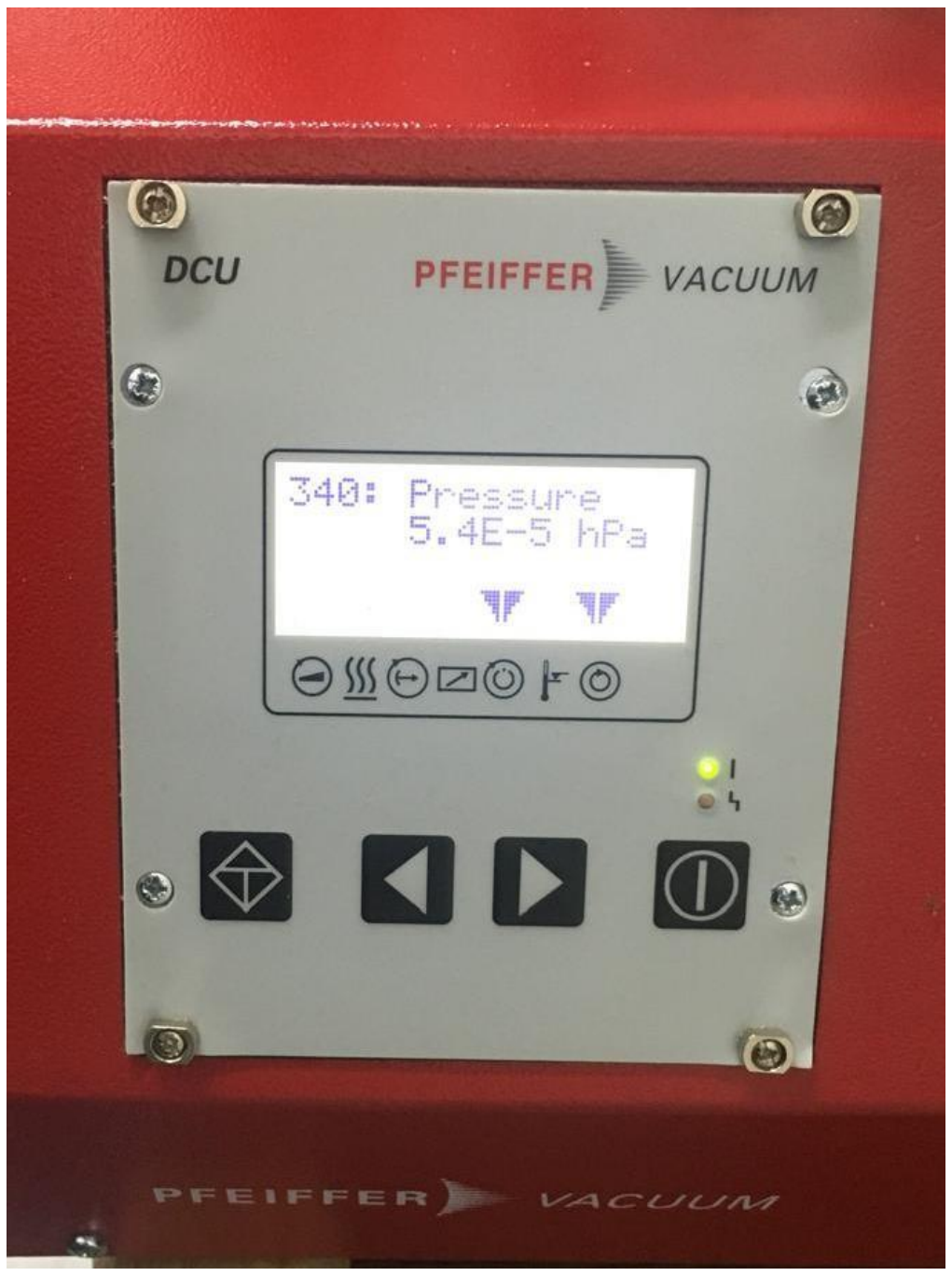

Figure 1. Representative image of vacuum system, laser configuration, and radial Cu-strip arrangement.

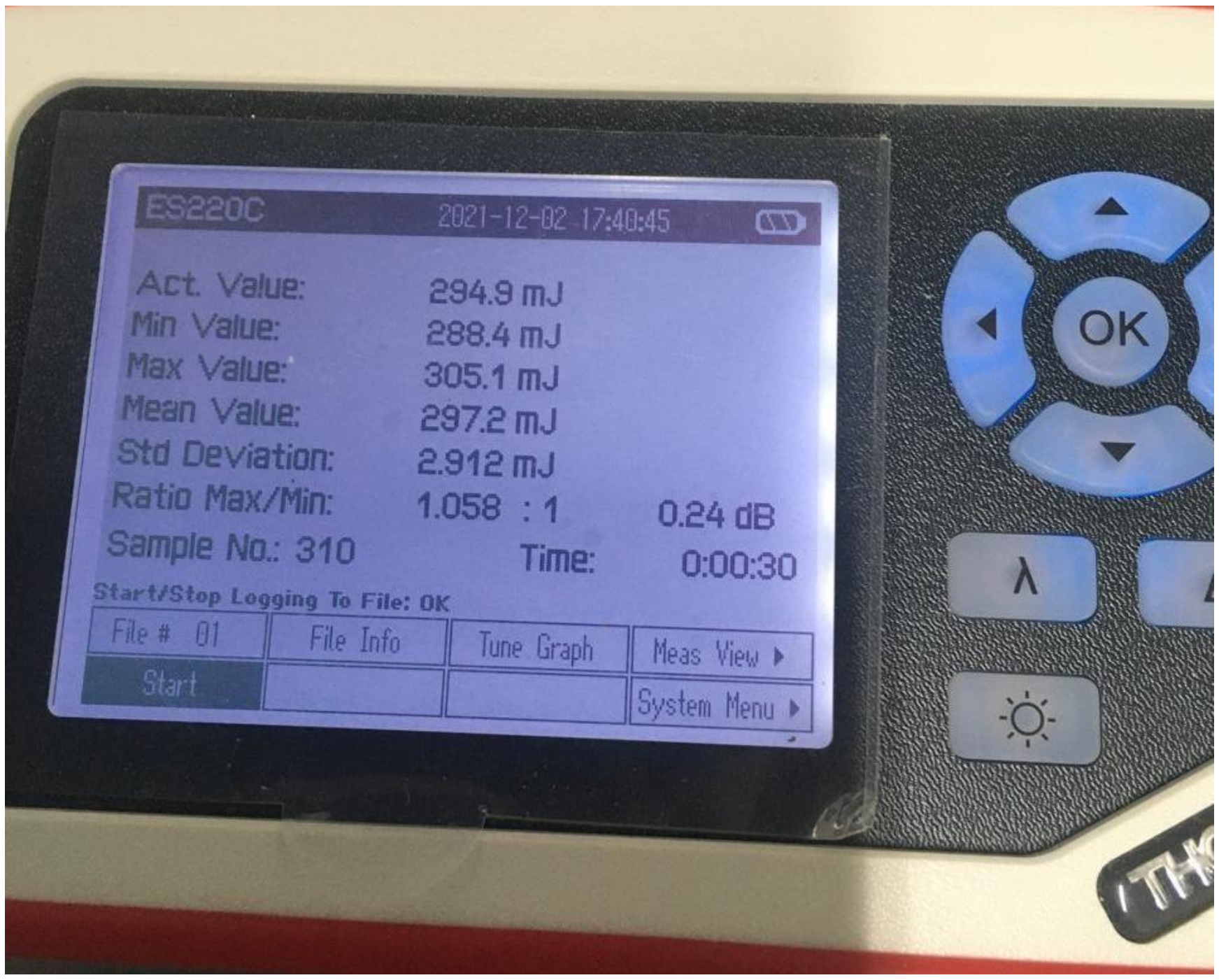

Figure 2. Representative image of vacuum system, laser configuration, and radial Cu-strip arrangement.

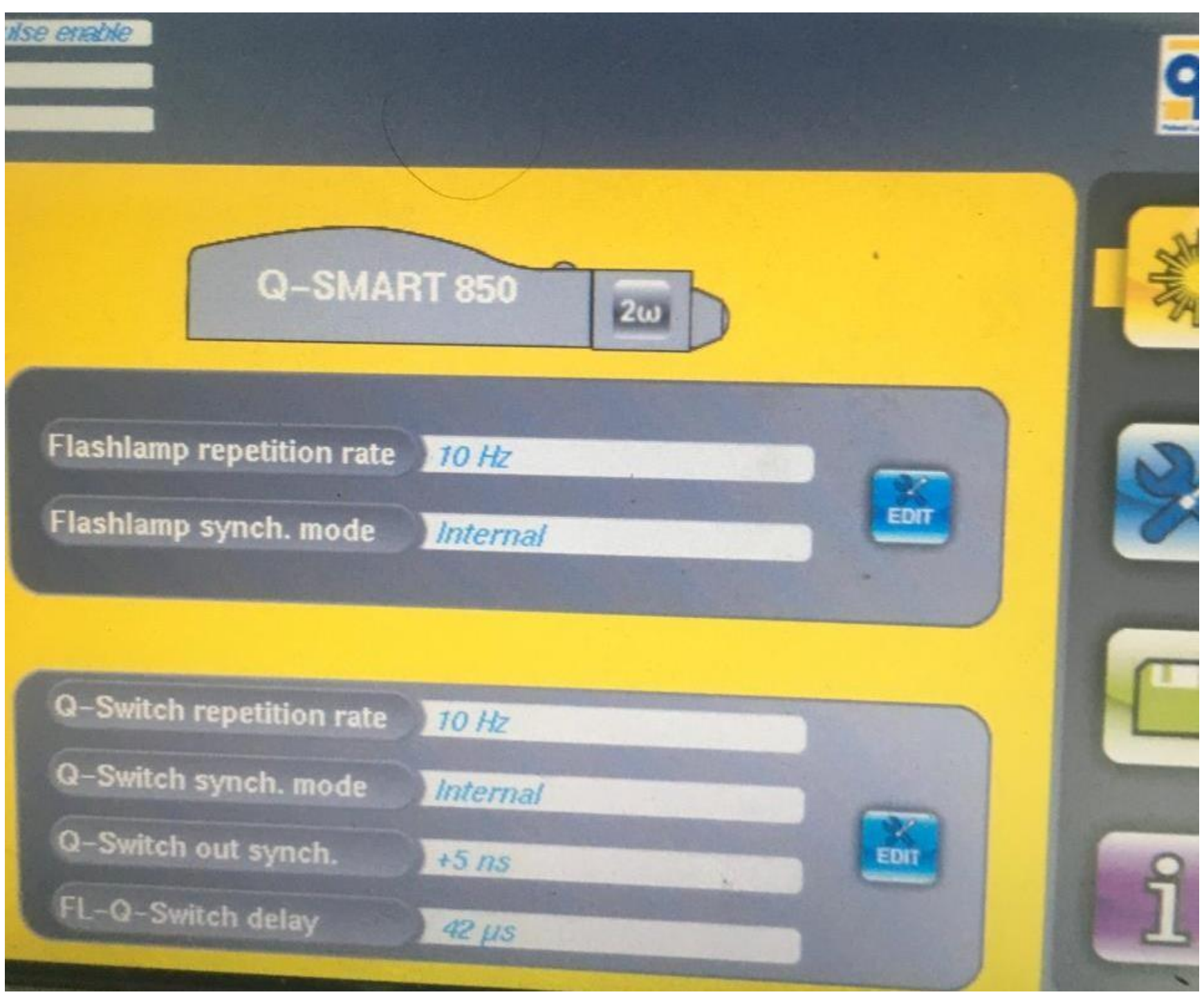

Figure 3. Representative image of vacuum system, laser configuration, and radial Cu-strip arrangement.

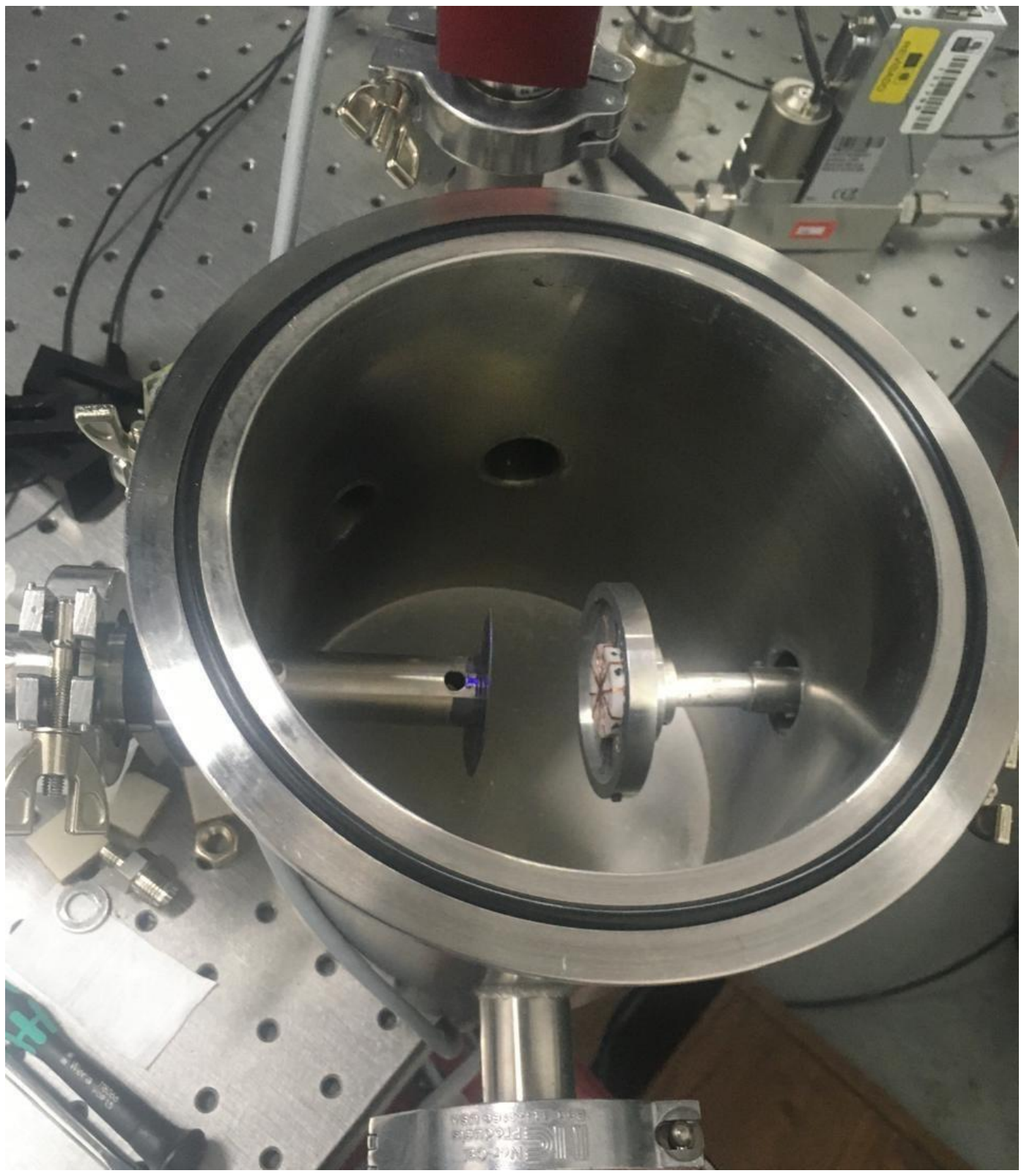

Figure 4. Representative image of vacuum system, laser configuration, and radial Cu-strip arrangement.

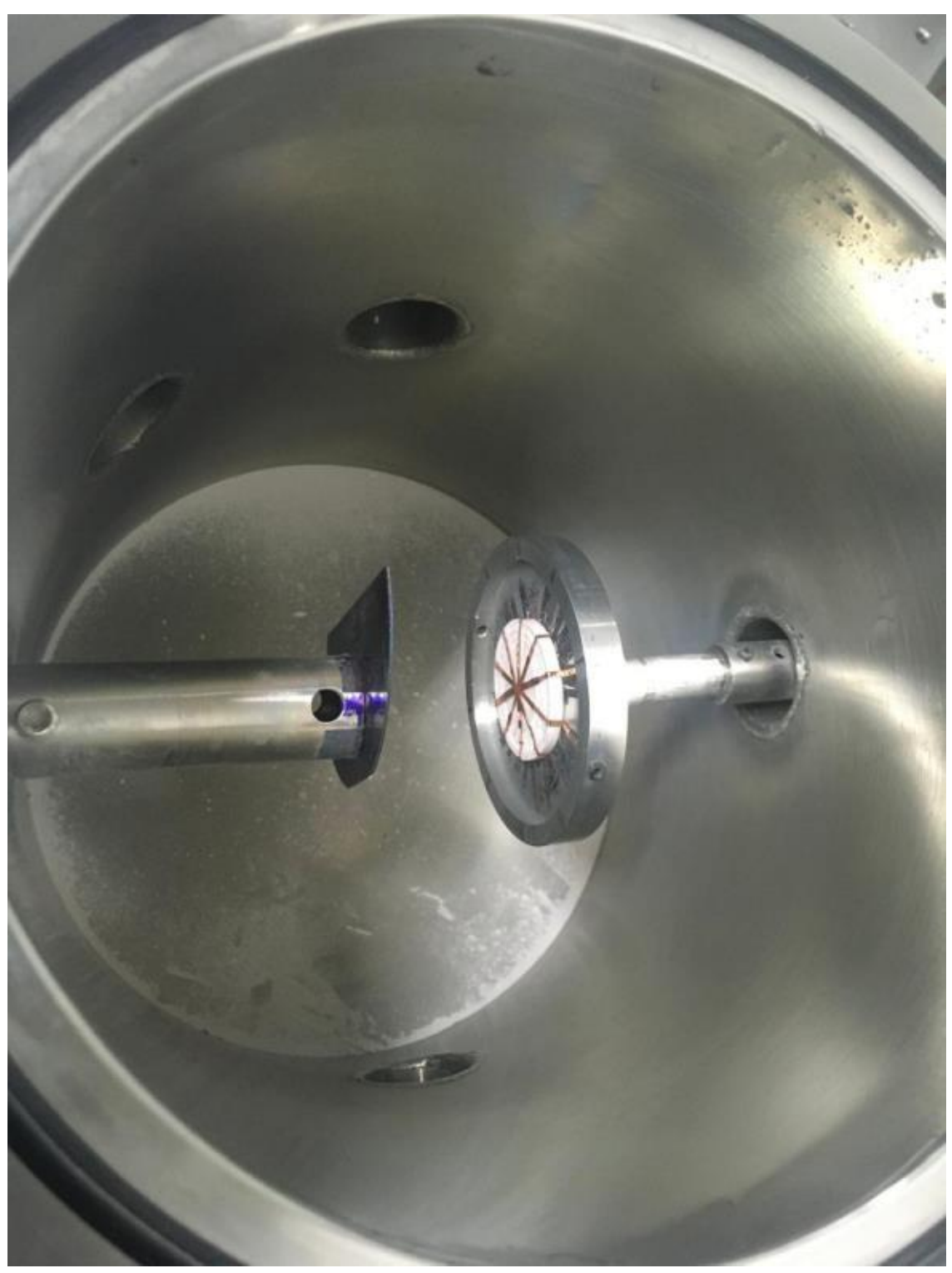

Figure 5. Representative image of vacuum system, laser configuration, and radial Cu-strip arrangement.

6.8 Post-deposition handling

40. Cool the substrates inside the chamber for at least 30 min.
41. Slowly vent the chamber to atmospheric pressure using dry nitrogen to limit moisture adsorption on the fresh films.

42. Remove the samples with clean tweezers and store them in sealed containers (Petri dishes or sample boxes) until characterization.

7. Expected results and verification points

- A visual inspection was performed for interference colors or visible coating on the bare Si, indicating film formation. Additionally, surface and quantization techniques were used in this case, energy dispersive X-ray spectroscopy (EDS) for the detection of Ca, P, O, and Cu peaks (Si from the substrate) and scanning electron microscopy (SEM) on the continuous coating with nano to submicrometric particles typical of PLD at room temperature.
- Finally, a structural analysis with XRD to identify hydroxyapatite peaks without prominent crystalline peaks of Cu/CuO (Cu may be amorphous or below the detection limit).

8. Troubleshooting (common problems)

Problem	Probable cause	Action/mitigation
Target cracks during sintering	Thermal stress; rapid ramps; inhomogeneous green compact	Ensure uniform pressing; follow slow ramps (3 °C/min up to 700 °C and 1 °C/min up to 1150 °C); allow to cool with the furnace closed.
No film observed / very low deposition rate	Laser misalignment; low fluence; shutter closed; target out of focus	Realign the beam; confirm energy on target; measure spot size; check lens position and window cleanliness.
Unstable pen / excess particles	Localized perforation; insufficient rastering; contaminated target surface	Increase rastering coverage; verify rotation at 10 rpm; gently polish/clean the surface; reduce energy per pulse if necessary.
Copper is not detected by EDS.	The strips are not ablated (poor contact/geometry); few strips; short run	Improve contact; reposition so that the beam intermittently intersects Cu during rotation; increase the number of strips within the study design; extend the deposition time consistently.
Low adhesion/detachment	Substrate contamination; rust regrowth; handling	Minimize the time between HF bath and loading; do not touch the active surface; consider in situ cleaning with plasma/ions if available.
Pressure peaks or surges	Leaks; adhesive outgassing; contaminated chamber	Check for leaks; bake/clean the chamber; avoid adhesives that are not vacuum compatible; ensure adequate pumping and throttling

9. Expected results and verification points

- Target ID, number of Cu strips, dimensions, and photo of the layout.
- Base pressure, working pressure, and method of maintaining it (throttling/gas admission).
- Laser configuration (wavelength, energy per pulse, frequency), measured spot size, and calculated fluence.
- Rotation / rastering parameters and total deposition time.
- Substrate cleaning date/time and elapsed time between the HF bath and loading.

10. Reference

Bohorquez-Santiago L, Devia-Narvaez DM, Ospina-Ospina R. A detailed method for copper doping of synthetic hydroxyapatite thin films via plasma-assisted pulsed laser deposition (PAPVD). Manuscript submitted to PLOS ONE (PONE-D-26-06266).
