# Supplementary material for: A detailed method for copper doping of synthetic hydroxyapatite thin films via plasma-assisted pulsed laser deposition
Source: PLoS One. 2026 Sep 25;21(9):e0358441. doi: 10.1371/journal.pone.0358441 (PMC13614653; doi:10.1371/journal.pone.0358441)
Supplement: Supplementary file 3 [file pone.0358441.s006.pdf]

Pereira, june 17 de 2026

Subject: Response to Reviewers' Comments on the Author

Below are the comments made by the reviewers, listed in the order they were received via email. In this letter, they are copied exactly as received, including their numbers for better organization. Those requiring a response from the author are addressed under the corresponding number with the subheading "Response" in bold. We thank the reviewers in advance for their comments and observations and hope to resolve any questions that may have arisen.

Reviewers' comments:

1. **Does the manuscript report a protocol which is of utility to the research community and adds value to the published literature?**

Reviewer #1: Yes

Reviewer #2: Yes

---

2. **Has the protocol been described in sufficient detail?**

To answer this question, please click the link to protocols.io in the Materials and Methods section of the manuscript (if a link has been provided) or consult the step-by-step protocol in the Supporting Information files.

The step-by-step protocol should contain sufficient detail for another researcher to be able to reproduce all experiments and analyses.

Reviewer #1: Yes

Reviewer #2: Partly

---

3. **Does the protocol describe a validated method?**

The manuscript must demonstrate that the protocol achieves its intended purpose: either by containing appropriate validation data, or referencing at least one original research article in which the protocol was used to generate data.

Reviewer #1: Yes

Reviewer #2: Yes

---

4. If the manuscript contains new data, have the authors made this data fully available?

The PLOS Data policy requires authors to make all data underlying the findings described in their manuscript fully available without restriction, with rare exception (please refer to the Data Availability Statement in the manuscript PDF file). The data should be provided as part of the manuscript or its supporting information, or deposited to a public repository. For example, in addition to summary statistics, the data points behind means, medians and variance measures should be available. If there are restrictions on publicly sharing data—e.g. participant privacy or use of data from a third party—those must be specified.

Reviewer #1: Yes

Reviewer #2: No

---

5. Is the article presented in an intelligible fashion and written in standard English?

PLOS ONE does not copyedit accepted manuscripts, so the language in submitted articles must be clear, correct, and unambiguous. Any typographical or grammatical errors should be corrected at revision, so please highlight any specific errors that need correcting in the box below.

Reviewer #1: No: The manuscript lacks a sufficiently detailed experimental methodology for the copper doping of synthetic hydroxyapatite thin films using plasma-assisted pulsed laser deposition (PA-PLD). The authors should provide comprehensive deposition parameters, including target composition, laser wavelength and fluence, substrate temperature, plasma conditions, chamber pressure, target-to-substrate distance, deposition time, and post-deposition treatment to ensure reproducibility of the work.

Response: We thank the reviewer for this valuable comment. The manuscript has been revised to provide a more detailed description of the deposition methodology. Additional information regarding the target composition (sintered hydroxyapatite target modified with high-purity copper strips), laser wavelength (532 nm), pulse duration (6 ns), fluence (30 $J\cdot cm^{-2}$), substrate temperature (room temperature), chamber pressure (6.3×10^{-5} mbar), target-to-substrate distance (3 cm), target rotation speed (60 rpm), deposition time (5 min), plasma plume conditions, and post-deposition handling has been incorporated into Section 3. These additions improve the reproducibility and transparency of the proposed PAPVD process.

Reviewer #2: No: English is fair, the presentation is not good, the restructuring of manuscript is highly recommended.

6. Review Comments to the Author

Please use the space provided to explain your answers to the questions above. You may also include additional comments for the author, including concerns about dual publication, research ethics, or publication ethics. (Please upload your review as an attachment if it exceeds 20,000 characters)

Reviewer #1: The manuscript lacks a sufficiently detailed experimental methodology for the copper doping of synthetic hydroxyapatite thin films using plasma-assisted pulsed laser deposition (PA-PLD). The authors should provide comprehensive deposition parameters, including target composition, laser wavelength and fluence, substrate temperature, plasma conditions, chamber pressure, target-to-substrate distance, deposition time, and post-deposition treatment to ensure reproducibility of the work.

Response:

We thank the reviewer for this valuable comment. The manuscript has been substantially revised to provide a more comprehensive and reproducible description of the PAPVD deposition methodology.

Specifically, the following experimental details have been incorporated into Section 3 (Doping and Deposition Process):

- Composition of the deposition target, consisting of a sintered hydroxyapatite (HAp) ceramic target modified with high-purity copper strips (99.99%) to enable in situ copper incorporation.
- Nd:YAG laser operating wavelength (532 nm) and pulse duration (6 ns).
- Laser fluence ($30\ J \cdot cm^{-2}$), pulse repetition rate (10 Hz), and pulse energy (85 mJ).
- Chamber base pressure ($< 5 \times 10^{-6}$ mbar) and working pressure (6.3×10^{-5} mbar).
- Target-to-substrate distance (3 cm).
- Deposition on silicon substrates maintained at room temperature without intentional heating.
- Target rotation speed (60 rpm) during deposition to ensure homogeneous ablation and stable plasma plume generation.
- Deposition time (5 min).
- Post-deposition treatment, consisting of cooling inside the vacuum chamber for 30 min followed by controlled venting with high-purity nitrogen, without additional thermal annealing.

In addition, a summary paragraph containing the principal deposition parameters has been added to facilitate reproducibility and improve the clarity of the experimental procedure

Reviewer #2:

1. What was the rationale behind selecting copper filaments as the dopant source instead of using pre-mixed copper-containing hydroxyapatite targets?

Response: We thank the reviewer for this important question. The use of metallic copper filaments placed on the surface of the hydroxyapatite target was intentionally selected as a flexible and cost-effective strategy for controlling copper incorporation during PAPVD deposition.

Unlike pre-mixed HAp-Cu ceramic targets, the filament-based approach allows the copper content to be modified simply by changing the number and arrangement of the copper strips without requiring the synthesis, pressing, and sintering of a new ceramic target for each composition. This significantly reduces material preparation time and facilitates rapid optimization of deposition conditions.

In addition, the use of metallic copper strips minimizes potential modifications of the hydroxyapatite crystalline structure that may occur during the high-temperature processing required for the fabrication of pre-doped ceramic targets. By maintaining the hydroxyapatite target composition unchanged and introducing copper only during laser ablation, the methodology enables a more direct evaluation of the influence of copper incorporation on the deposited films.

Furthermore, the intermittent co-ablation of hydroxyapatite and copper during target rotation provides a reproducible route for generating Cu-containing coatings while preserving the simplicity and adaptability of the deposition process. This approach was therefore selected as a practical methodology-oriented solution aimed at facilitating reproducibility and experimental flexibility.

2. How was the concentration of copper within the deposited HAp-Cu thin films controlled and monitored during the PAPVD process?

   Response: We thank the reviewer for this important question. The copper concentration was controlled through the deliberate modification of the hydroxyapatite target surface using a defined number of high-purity copper strips (2–6 strips), symmetrically distributed over the circular HAp target. This configuration was designed to promote homogeneous and reproducible co-ablation of both hydroxyapatite and copper during laser irradiation.

   The circular geometry of the target, combined with continuous target rotation during deposition, ensured that the laser beam periodically interacted with both the HAp surface and the copper strips, promoting a uniform incorporation of copper-containing species into the plasma plume. Consequently, the copper content in the deposited films was controlled by varying the number of copper strips while maintaining identical deposition conditions for all samples.

   Copper incorporation was subsequently monitored and verified by Energy Dispersive X-ray Spectroscopy (EDS), which confirmed the presence of copper in the deposited coatings. The systematic increase in the number of copper strips resulted in progressively higher copper incorporation levels, as reported in our complementary characterization study [1].

   The copper incorporation level was controlled by varying the number of copper strips placed on the target surface while maintaining all other deposition parameters constant. The symmetric radial arrangement of the copper strips, combined with continuous target rotation during deposition, promoted a reproducible co-ablation process and a homogeneous

distribution of copper-containing species within the plasma plume. Copper incorporation into the deposited films was subsequently verified by EDS analysis.

3. Did the incorporation of copper filaments influence the ablation behavior or stability of the hydroxyapatite target during repeated laser exposure?

   Response: We thank the reviewer for this insightful question. The incorporation of copper filaments did not produce observable instabilities in the ablation process nor compromise the structural integrity of the hydroxyapatite target during repeated laser irradiation cycles.
   Although copper and hydroxyapatite exhibit different thermal conductivities, optical absorption coefficients, and laser–material interaction characteristics, the symmetric distribution of the copper strips over the target surface, together with continuous target rotation and rastering, promoted a homogeneous ablation process throughout deposition.
   During the PAPVD experiments, no significant fluctuations in plasma plume formation, pressure stability, or deposition behavior were observed when compared with the undoped hydroxyapatite target. Furthermore, no evidence of target cracking, delamination of the copper strips, or localized damage that could adversely affect the deposition process was detected.
   These observations indicate that the incorporation of copper filaments is compatible with stable and reproducible PAPVD operation under the selected experimental conditions.

4. What measures were taken to ensure uniform distribution of copper throughout the deposited thin film?

   Response: We thank the reviewer for this important question. Several experimental measures were implemented to promote a homogeneous incorporation of copper throughout the deposited HAp-Cu films.
   First, the copper strips were symmetrically distributed over the surface of the circular hydroxyapatite target. This configuration was specifically designed to favor the homogeneous co-ablation of hydroxyapatite and copper during laser irradiation, minimizing localized compositional variations associated with preferential ablation.
   Second, continuous target rotation and rastering were employed during deposition, ensuring periodic interaction of the laser beam with both the hydroxyapatite surface and the copper strips. This dynamic ablation strategy contributed to the generation of a stable plasma plume containing species from both materials and reduced the likelihood of localized copper enrichment.
   Finally, critical deposition parameters, including laser fluence, chamber pressure, target-to-substrate distance, and deposition time, were maintained constant throughout all experiments to ensure process stability and reproducibility.
   The effectiveness of these measures was supported by EDS characterization, which confirmed the successful incorporation of copper within the deposited coatings.

5. Why was silicon selected as the substrate material, and how might the deposition behavior differ on metallic or biomedical implant substrates?

Response: We thank the reviewer for this important question. Silicon was selected as the substrate material because it provides a well-defined, smooth, chemically stable, and reproducible surface that is widely used in thin-film deposition studies. The use of silicon minimizes the influence of substrate-related variables and facilitates the characterization of the deposited coatings by techniques such as SEM, EDS, XRD, AFM, and profilometry.

In the present work, the primary objective was to establish and validate a reproducible methodology for the fabrication of copper-doped hydroxyapatite thin films by PAPVD. Therefore, silicon was employed as a model substrate to focus on the deposition process itself and the incorporation of copper into the hydroxyapatite matrix.

We recognize that deposition behavior may differ on metallic substrates commonly used in biomedical applications, such as titanium and titanium alloys. Factors including surface roughness, thermal conductivity, surface energy, native oxide layers, and differences in thermal expansion coefficients may influence nucleation, film adhesion, growth kinetics, residual stress development, and coating morphology. Nevertheless, the fundamental co-ablation and copper-incorporation strategy described in this work is expected to remain applicable to metallic implant materials, although further optimization of deposition parameters may be required for each specific substrate.

This clarification has been incorporated into the revised manuscript.

6. Were there any observable changes in crystallinity, phase purity, or preferred orientation of hydroxyapatite after copper doping?

Response: We thank the reviewer for this important question. XRD analysis revealed that all deposited coatings maintained the characteristic crystalline structure of hydroxyapatite, and no additional crystalline phases associated with copper, copper oxides, or other calcium phosphate compounds were detected. These results indicate that the phase purity of hydroxyapatite was preserved after copper incorporation.

Although no significant shifts were observed in the position of the HAp (002) diffraction peak, variations in the full width at half maximum (FWHM) and crystallite size were detected among the Cu-containing samples. These changes suggest that copper incorporation influenced the crystalline quality of the coatings without inducing structural phase transformations.

Furthermore, the absence of significant changes in the (002) peak position indicates that copper was not substantially incorporated into the hydroxyapatite crystal lattice. Instead, the combined XRD and XPS results suggest that copper was mainly present at the coating surface or in an amorphous form associated with the hydroxyapatite matrix.

Therefore, copper incorporation modified the crystallinity-related parameters of the coatings while preserving the hydroxyapatite phase composition and without evidence of significant changes in preferred crystallographic orientation.

7. How sensitive is the proposed methodology to variations in laser fluence, repetition rate, or chamber pressure?

Response: We thank the reviewer for this valuable question. The primary objective of the present study was to demonstrate the feasibility of copper incorporation into hydroxyapatite thin films using the proposed PAPVD methodology rather than to perform a systematic

optimization of the deposition parameters. Therefore, laser fluence, repetition rate, and chamber pressure were maintained constant throughout all experiments.
Nevertheless, these parameters are known to strongly influence the ablation rate, plasma plume dynamics, energy transfer, and ultimately the composition and morphology of the deposited coatings. Consequently, variations in these parameters are expected to affect the deposition process and the resulting film characteristics. A detailed parametric study evaluating the sensitivity of the methodology to these variables is considered an important topic for future work.

Was the adhesion strength of the HAp-Cu thin films to the substrate evaluated or qualitatively assessed during the study?
Response: We thank the reviewer for this important observation. Quantitative adhesion measurements, such as scratch testing, pull-off testing, or nanoindentation-based adhesion analysis, were not performed in the present study, as the primary objective was to establish and validate the PAPVD methodology for the fabrication of Cu-doped hydroxyapatite thin films.
Nevertheless, qualitative observations were carried out during sample handling and subsequent characterization by SEM, EDS, and XRD. No visible film delamination, peeling, cracking, or spontaneous detachment from the silicon substrates was observed during these procedures. Furthermore, SEM analysis revealed continuous and relatively dense coatings, suggesting adequate film adherence for characterization purposes.
We acknowledge that quantitative adhesion testing would provide valuable information regarding the mechanical integrity of the coatings and therefore represents an important topic for future work, particularly for biomedical coating applications.

8. Could the proposed PAPVD methodology be extended to incorporate other metallic dopants such as silver, zinc, or magnesium for multifunctional bioactive coatings?

   Response: We thank the reviewer for this interesting question. In principle, the proposed PAPVD methodology is not restricted to copper and could be extended to other metallic dopants such as silver, zinc, magnesium, or combinations thereof. The strategy employed in this work relies on the physical incorporation of metallic sources onto the hydroxyapatite target surface, allowing their simultaneous ablation with hydroxyapatite during the deposition process.
   However, the successful implementation of alternative dopants would require optimization of the deposition parameters because each material exhibits different laser–matter interaction characteristics, including optical absorption, ablation threshold, thermal conductivity, melting temperature, vaporization behavior, and plasma plume dynamics. These factors may influence dopant incorporation efficiency, film composition, and coating morphology.
   Nevertheless, the general methodology described in this work provides a flexible platform that could potentially be adapted for the fabrication of multifunctional hydroxyapatite-based coatings incorporating antibacterial, osteogenic, angiogenic, or other therapeutic metallic species. The exploration of alternative dopants represents an interesting direction for future studies.

9. What are the main limitations or challenges associated with scaling this copper-doping methodology for larger-area coatings or industrial biomedical applications?

Response: We thank the reviewer for this important question. Although the proposed PAPVD methodology proved effective for the fabrication of Cu-doped hydroxyapatite thin films at laboratory scale, several challenges must be addressed before large-scale implementation.

First, maintaining a homogeneous copper distribution over larger substrate areas may become increasingly difficult due to the localized nature of laser ablation and the use of discrete copper strips as the dopant source. Larger-area coatings would require optimization of the target geometry, scanning strategy, and substrate motion to ensure compositional uniformity.

Second, prolonged operation may lead to non-uniform target erosion and variations in the relative ablation rates of hydroxyapatite and copper, potentially affecting the reproducibility of film composition during extended deposition campaigns.

Third, industrial-scale production would require improvements in deposition throughput, including larger targets, automated target replacement systems, higher-power laser sources, or multi-beam configurations to increase coating area and productivity.

From a biomedical perspective, additional studies would be necessary to evaluate coating adhesion, mechanical durability, long-term stability, corrosion resistance, biocompatibility, and regulatory compliance on clinically relevant substrates such as titanium and titanium alloys.

Therefore, while the proposed methodology demonstrates considerable flexibility and reproducibility at the laboratory scale, further engineering development and application-specific optimization would be required for large-scale industrial implementation.

10. Restructure the manuscript in such a way it is easy to read, also present in the order Title, Abstract, Keywords, Introduction, Methods and materials, Results and Discussion, Conclusions, references.

Response: We thank the reviewer for this valuable suggestion. The manuscript has been extensively reorganized to improve its readability, logical flow, and overall clarity.

In the revised version, the manuscript now follows the recommended structure:

- Title
- Abstract
- Keywords
- Introduction
- Materials and Methods
- Results and Discussion
- Conclusions
- References

To address this comment, the previous methodology-centered organization was restructured into a conventional scientific article format. All experimental procedures, including hydroxyapatite target preparation, substrate cleaning, PAPVD experimental setup, copper doping strategy, deposition protocol, and post-deposition handling, have been consolidated within the Materials and Methods section. Likewise, the validation of the proposed methodology through visual inspection, SEM, EDS, and XRD analyses has been integrated into the Results and Discussion section.

In addition, a dedicated Conclusions section has been incorporated to summarize the main methodological contributions and key findings of the study. Section numbering, subsection

organization, and figure placement were also revised to improve the continuity of the manuscript and facilitate navigation for readers.
These modifications were implemented to enhance the overall accessibility, readability, and scientific presentation of the manuscript.

11. Check the grammatical errors and do a thorough proof reading

    Response: We thank the reviewer for this observation. The manuscript has been carefully revised and thoroughly proofread to correct grammatical, typographical, punctuation, and formatting errors.
    In the revised version, we reviewed sentence structure, terminology consistency, figure citations, section numbering, unit notation, spacing, and overall readability. Particular attention was given to improving the clarity of the Materials and Methods and Results and Discussion sections, ensuring that the experimental procedure and methodological validation are presented in a clearer and more coherent manner.
    These revisions were made to improve the scientific readability and overall quality of the manuscript.

12. Also, no need to use reference in abstract itself, remove that citation

    Response: We thank the reviewer for this observation. The abstract has been revised accordingly, and the citation previously included in the abstract has been removed. References are now cited exclusively within the main body of the manuscript, in accordance with the journal guidelines. This modification improves the readability of the abstract and ensures consistency with the recommended manuscript format.

13. State the novelty clearly and highlight the difference between already conducted studies and your study.

    Response: We thank the reviewer for this valuable observation, which has allowed us to further clarify the contribution and novelty of the present work.
    The principal novelty of this study lies in the implementation of an experimental PAPVD methodology for the fabrication of Cu-doped hydroxyapatite thin films through the direct incorporation of metallic copper strips onto the surface of a hydroxyapatite target. During laser irradiation, this configuration promotes the simultaneous ablation of hydroxyapatite and copper, enabling in situ copper incorporation during film growth without requiring the prior synthesis of Cu-containing hydroxyapatite powders or the fabrication of pre-doped ceramic targets.
    In contrast to many previously reported approaches, where metallic ions are incorporated into hydroxyapatite through chemical synthesis routes, ion-substitution processes, or the preparation of pre-doped targets before deposition, the methodology proposed here introduces a direct and flexible co-ablation strategy in which the dopant source is incorporated physically into the target assembly immediately prior to deposition.
    An additional advantage of this approach is that the copper incorporation level can be progressively modified by varying the number of copper strips placed on the hydroxyapatite target while maintaining the remaining deposition parameters unchanged. This provides a

simple route for controlling dopant incorporation without the need to fabricate multiple targets with different compositions.

Furthermore, while our previous publication focused on the physicochemical, structural, surface, and biological characterization of the resulting HAp-Cu coatings [1], the objective of the present manuscript is to provide a detailed, reproducible, and transferable methodology describing target preparation, dopant incorporation, PAPVD configuration, deposition conditions, and validation procedures.

Therefore, the contribution of the present work is not only the demonstration of a viable copper incorporation strategy for hydroxyapatite thin films, but also the establishment of a reproducible PAPVD methodology that may serve as a platform for future studies involving metallic-doped hydroxyapatite coatings and multifunctional bioactive thin films.

This letter addresses all the comments made by the reviewers and clarifies that the requested adjustments to the main document will be made according to the feedback provided and in accordance with the responses given in this letter. We again thank the reviewers for their valuable contributions to improving the quality of the article. We look forward to your feedback.

Leonardo Bohorquez S.

Firmado digitalmente por LEONARDO BOHORQUEZ SANTIAGO
Fecha: 2026.06.17 10:24:05 -05'00'

Leonardo Bohorques Santiago
Sing
lbohorquez@utp.edu.co
