# Supplementary material for: A detailed method for copper doping of synthetic hydroxyapatite thin films via plasma-assisted pulsed laser deposition
Source: PLoS One. 2026 Sep 25;21(9):e0358441. doi: 10.1371/journal.pone.0358441 (PMC13614653; doi:10.1371/journal.pone.0358441)
Supplement: Supplementary file 4 [file pone.0358441.s007.pdf]

Response to Reviewers

Manuscript ID: PONE-D-26-06266R1

Manuscript title: *Detailed method for copper doping of synthetic hydroxyapatite thin films by plasma-assisted pulsed laser deposition*

Dear Dr. Mehnath Sivaraj and Reviewers,

We sincerely thank the Academic Editor and the Reviewers for their careful evaluation of our revised manuscript and for their constructive comments. We greatly appreciate the positive assessment of the scientific and methodological quality of our work.

In response to the remaining comment, Figures 6, 7, and 8 have been replaced with improved, high-resolution versions. The changes made in the revised manuscript are described below. The corresponding modifications are shown in the marked-up version of the manuscript.

Reviewer #1

Comment:
"The manuscript contains comprehensive experimental data with full methodological justification, including synthesis, deposition, and characterization of copper-doped hydroxyapatite thin films, providing sufficient evidence to support the conclusions and warrant publication."

Response:
We sincerely thank the Reviewer for the positive assessment of our manuscript and for recognizing the methodological detail, experimental validation, and scientific relevance of the presented protocol. We appreciate the Reviewer's time and careful evaluation of our work.

Reviewer #2

Comment:
"Figure 6, Figure 7 and Figure 8 image qualities are very poor, Improve Resolution or replace it. The other comments have been addressed well."

Response:
We thank the Reviewer for this important observation. In response to the comment, Figures 6, 7, and 8 have been replaced with improved, high-resolution versions. The revised figures were prepared from the original source files to enhance their sharpness, resolution, and overall readability while preserving the original scientific information and interpretation.

The updated figures have been incorporated into both the marked-up and clean versions of the revised manuscript. In addition, Figures 6, 7, and 8 have been prepared as separate publication-quality files and will be uploaded individually to the submission system, replacing the previously submitted versions.

Changes in the revised manuscript:

- Figure 6 was replaced with a higher-resolution version.
- Figure 7 was replaced with a higher-resolution version.
- Figure 8 was replaced with a higher-resolution version.

- No changes were made to the scientific results, figure interpretation, or conclusions associated with these figures.

Location in the revised manuscript: Figure 6: page 20; Figure 7: page 22; Figure 8: page 24.

We thank the Reviewer again for the helpful recommendation, which has improved the visual quality and presentation of the manuscript.

Journal Requirements

The reference list has been reviewed to ensure its completeness, accuracy, and correspondence with the citations included in the manuscript. No references were added solely at the request of the Reviewers, as no specific publications were recommended for citation.

We hope that the revised manuscript and the improved figures satisfactorily address the remaining concern. We sincerely thank the Academic Editor and the Reviewers for their valuable time and consideration.

Sincerely,

Leonardo Bohorquez Santiago
Firmado digitalmente por Leonardo Bohorquez Santiago
Fecha: 2026.07.30 14:46:09 -05'00'

Leonardo Bohórquez Santiago
On behalf of all authors
