# Supplementary material for: A detailed method for copper doping of synthetic hydroxyapatite thin films via plasma-assisted pulsed laser deposition
Source: PLoS One. 2026 Sep 25;21(9):e0358441. doi: 10.1371/journal.pone.0358441 (PMC13614653; doi:10.1371/journal.pone.0358441)
Supplement: Supplementary file 5 [file pone.0358441.s008.pdf]

Pereira, 24 de august de 2026

PLOS One

Revision required [PONE-D-26-06266R2]

Subject: answer to Review Comments to the Author.

We appreciate the comments provided. To address them, we will respond to each reviewer's observations point by point. This approach will clarify the issues raised and the scope of the responses.

Response to Reviewer #1

We sincerely thank Reviewer #1 for the careful evaluation of our manuscript and for the constructive comments. We have revised the manuscript accordingly. Our point-by-point responses are provided below.

- *Inconsistent terminology — "Plasma-Assisted Pulsed Laser Deposition (PAPVD)" in abstract vs "Plasma-Assisted Physical Vapor Deposition (PAPVD)" in introduction. PAPVD = Plasma-Assisted Pulsed Laser Deposition (a variant of PLD, not PVD)*

Response:

We thank the Reviewer for identifying this inconsistency. We agree that the terminology should be used consistently throughout the manuscript. Accordingly, all instances in which PAPVD was incorrectly expanded as "Plasma-Assisted Physical Vapor Deposition" have been corrected to "Plasma-Assisted Pulsed Laser Deposition." The terminology has been standardized in the Keywords, Introduction, Specifications Table, Materials and Methods,

Conclusions, and method title. These corrections are terminological only and do not affect the experimental methodology, results, or conclusions of the manuscript.

- *Ca/P ratio (1.67) mentioned but no verification method provided. Add XRD or FTIR confirmation of HAp phase after synthesis*

Response:

We thank the Reviewer for this important observation. We have clarified that the Ca/P molar ratio of 1.67 refers to the stoichiometric precursor ratio selected for hydroxyapatite synthesis and not to an experimentally measured Ca/P ratio of the final material. Direct XRD or FTIR characterization of the as-synthesized powder was not performed as a separate measurement in the present methodological study. Therefore, to avoid overstating the available evidence, the revised manuscript now states that the hydroxyapatite phase obtained using this synthesis route and subsequently deposited as thin films was experimentally verified in our previously published complementary study [1]. In that work, XRD confirmed the characteristic crystalline HAp phase and the diffraction patterns were indexed according to PDF No. 00-009-0432; FTIR also showed the characteristic phosphate vibrational bands at approximately 1024 and 960 cm^{-1}. This clarification has been added to Section 2.1.1 of the revised manuscript.

- *Cooling rate missing — "allowed to cool naturally" should specify ~3-5°C/min for 1. Line 357.*

Response:

We thank the Reviewer for this observation. The cooling procedure has been clarified in the revised manuscript. Following the 2 h dwell at 1150 °C, the furnace was switched off and the HAp targets were allowed to cool passively inside the closed furnace to room temperature over approximately 16 h. Because this cooling stage was not actively programmed, the cooling rate was not constant. Based on the overall temperature decrease and cooling time, the average cooling rate was approximately 1.2 °C/min. This information has been added to

Section 2.1.2 to improve reproducibility without introducing an unsupported programmed cooling rate.

- *Laser spot size stated as "~0.6 mm diameter" — INCONSISTENT with Line 236! Use single value.*

Response:

We thank the Reviewer for identifying this inconsistency. The laser spot size has been standardized throughout the manuscript to approximately 0.6 mm in diameter. The value previously reported as 0.7 mm in the Experimental Setup section has been corrected accordingly to ensure consistency with the deposition parameters and the reported laser fluence.

- *CRITICAL ERROR — Fluence calculation wrong. 85 mJ / 0.028 mm² = 3.03 J/cm², NOT 30 J/cm². Correct calculation or verify measurement*

Response:

We thank the Reviewer for identifying this inconsistency. After carefully verifying the laser parameters and calculation, we found that the error was in the reported irradiated spot area rather than in the laser fluence.

For a laser spot diameter of approximately 0.60 mm, the corresponding irradiated area is approximately 0.283 mm² (2.83×10^{-3} cm²), rather than 0.028 mm² as incorrectly stated in the previous version of the manuscript. Using an energy per pulse of 85 mJ, the resulting fluence is therefore:

$$F = 0.085 \text{ J} / 2.83 \times 10^{-3} \text{ cm}^2 \approx 30 \text{ J/cm}^2.$$

Accordingly, the spot area has been corrected from 0.028 mm² to 0.283 mm², and the laser spot diameter has been standardized to approximately 0.60 mm throughout the manuscript. The reported on-target fluence of approximately 30 J/cm² therefore remains unchanged.

- *No target density measurement — "dense targets" should be quantified: ~95% of theoretical density*

Response:

We thank the Reviewer for this important observation. The density of the sintered HAp targets was not experimentally measured; therefore, reporting a numerical relative density (for example, ~95% of the theoretical density) would not be supported by the available data. To avoid an unsupported quantitative claim, the term “dense” has been removed when describing the targets. They are now described as mechanically robust ceramic targets with sufficient structural integrity for PLD, which reflects the experimentally observed characteristics relevant to laser ablation. No numerical density value has been introduced.}

- *No film thickness measurement — essential for reproducibility (add cross-sectional SEM data)*

Response:

We thank the Reviewer for this important observation and agree that film thickness is a relevant parameter for the quantitative characterization of thin-film deposition. However, cross-sectional SEM and profilometric thickness measurements were not performed during the original experimental campaign, and the original coated specimens are no longer available for retrospective measurement. We therefore consider that providing an estimated thickness would be scientifically inappropriate.

To address this limitation transparently, the revised manuscript removes any statement that could imply that cross-sectional SEM measurements were available and explicitly adds film-thickness quantification to the Limitations section. The manuscript now states that no numerical film-thickness value is reported and recommends cross-sectional SEM or profilometry in future applications of the protocol. In addition, the experimentally controlled deposition parameters required to reproduce the process are reported in detail.

- *Give complage XRD patterns — add Miller indices (hkl) above peaks*

Response:

We thank the Reviewer for this valuable suggestion and agree that complete diffraction patterns with Miller-index assignments are important for phase identification. The complete XRD patterns corresponding to the HAp, HApCu-2, HApCu-3, HApCu-4, HApCu-5, and HApCu-6 coatings, including phase identification and Miller indices (hkl), were previously published and discussed in our complementary study [1] and cannot be reproduced in the present manuscript.

To address the Reviewer's concern without duplicating the previously published figure, we revised the XRD section to clearly distinguish the information presented in the current manuscript from that available in Ref. [1]. The section is now entitled "XRD-Derived Structural Parameters," and Figure 9 presents only the FWHM values and crystallite sizes derived from the (002) reflection. The text and figure caption explicitly direct readers to Ref. [1] for the complete diffraction profiles, phase identification, and Miller-index assignments. We also removed statements that could incorrectly imply that Figure 9 itself displays the complete XRD patterns.

- *Add a flowchart summarizing the entire experimental procedure from HAp synthesis to thin film characterization*

Response:

We thank the Reviewer for this valuable suggestion. To improve the clarity and readability of the manuscript, a new flowchart has been added summarizing the complete experimental procedure, from hydroxyapatite synthesis and target fabrication to copper-assisted deposition, post-deposition handling, and thin-film characterization.

This new schematic overview has been incorporated as Figure 1 and is referenced at the end of the Introduction. It provides a concise visual summary of the full workflow followed in the present methodological study.

- *Add a table of deposition parameters for quick reference (laser wavelength, fluence, repetition rate, pressure, distance, time, etc.)*

Response:

We thank the Reviewer for this helpful suggestion. A new table has been added to the Materials and Methods section to provide a concise summary of the principal PAPVD deposition parameters used throughout the experiments.

The table includes the laser wavelength, pulse duration, energy per pulse, laser spot size, fluence, repetition rate, chamber pressure, target-to-substrate distance, substrate temperature, target rotation speed, deposition time, and post-deposition conditions.

This addition provides a quick reference to the experimental conditions and improves the clarity and reproducibility of the described methodology.

- *Add a quantitative comparison table showing Cu content (EDS/ICP) for HApCu-2 through HApCu-6 samples*

Response:

We thank the reviewer for this important suggestion. To address this suggestion, a table showing the weight percentage of elements present in the deposited film obtained through SEM characterization in this research is included. This table allows for the quantitative identification of the amount of copper deposited in relation to the Ca/P ratio in the hydroxyapatite film, bearing in mind that most of this copper is presumed to be on the surface.

- *Add cross-sectional SEM images to show film thickness and uniformity*

Response:

We thank the Reviewer for this important suggestion. We agree that cross-sectional SEM imaging would provide valuable quantitative information regarding film thickness and through-thickness uniformity. However, cross-sectional SEM images were not acquired during the original experimental campaign, and the original coated specimens are no longer available for retrospective cross-sectional preparation and analysis. Therefore, it is not possible to provide reliable cross-sectional SEM images or film-thickness measurements at this stage without repeating the experimental deposition procedure.

To address this limitation transparently, the revised manuscript has been modified in two ways. First, the SEM section now explicitly states that the available SEM observations correspond to surface-view images and therefore cannot be used to determine film thickness or cross-sectional uniformity. Second, the Limitations section has been expanded to explicitly acknowledge that neither film thickness nor through-thickness uniformity was directly evaluated.

We have deliberately avoided providing an estimated film thickness or inferring cross-sectional uniformity from the available surface SEM images, as such conclusions would not be adequately supported by the experimental data. The revised manuscript now recommends cross-sectional SEM and/or profilometric measurements in future applications of the protocol to determine film thickness, evaluate cross-sectional uniformity, and establish the deposition rate.

Kind regards,

Leonardo Bohorquez S.

Firmado digitalmente por
LEONARDO BOHORQUEZ
SANTIAGO
Fecha: 2026.08.24 21:34:54 -05'00'

Leonardo Bohorquez Santiago
Sing
