# Supplementary material for: A detailed method for copper doping of synthetic hydroxyapatite thin films via plasma-assisted pulsed laser deposition
Source: PLoS One. 2026 Sep 25;21(9):e0358441. doi: 10.1371/journal.pone.0358441 (PMC13614653; doi:10.1371/journal.pone.0358441)
Supplement: Peer review history

# Peer review: Decision Letter 0

**DOI:** 10.1371/journal.pone.0358441.r001

**Authors:** Mehnath Sivaraj

13 May 2026

Dear Dr. Bohorquez Santiago,

Thank you for submitting your manuscript to PLOS ONE. After careful consideration, we feel that it has merit but does not fully meet PLOS ONE’s publication criteria as it currently stands. Therefore, we invite you to submit a revised version of the manuscript that addresses the points raised during the review process.

Please submit your revised manuscript by Jun 27 2026 11:59PM. If you will need more time than this to complete your revisions, please reply to this message or contact the journal office at plosone@plos.org. When you're ready to submit your revision, log on to https://www.editorialmanager.com/pone/ and select the 'Submissions Needing Revision' folder to locate your manuscript file.

- A letter that responds to each point raised by the academic editor and reviewer(s). You should upload this letter as a separate file labeled 'Response to Reviewers'.
- A marked-up copy of your manuscript that highlights changes made to the original version. You should upload this as a separate file labeled 'Revised Manuscript with Track Changes'.
- An unmarked version of your revised paper without tracked changes. You should upload this as a separate file labeled 'Manuscript'.

If you would like to make changes to your financial disclosure, please include your updated statement in your cover letter. Guidelines for resubmitting your figure files are available below the reviewer comments at the end of this letter.

If applicable, we recommend that you deposit your laboratory protocols in protocols.io to enhance the reproducibility of your results. Protocols.io assigns your protocol its own identifier (DOI) so that it can be cited independently in the future. For instructions see: https://journals.plos.org/plosone/s/submission-guidelines#loc-laboratory-protocols. Additionally, PLOS ONE offers an option for publishing peer-reviewed Lab Protocol articles, which describe protocols hosted on protocols.io. Read more information on sharing protocols at https://plos.org/protocols?utm_medium=editorial-email&utm_source=authorletters&utm_campaign=protocols.

As the corresponding author, your ORCID iD is verified in the submission system and will appear in the published article. PLOS supports the use of ORCID, and we encourage all coauthors to register for an ORCID iD and use it as well. Please encourage your coauthors to verify their ORCID iD within the submission system before final acceptance, as unverified ORCID iDs will not appear in the published article. *Only*  the individual author can complete the verification step; PLOS staff *cannot*  verify ORCID iDs on behalf of authors.

We look forward to receiving your revised manuscript.

Kind regards,

Mehnath Sivaraj

Academic Editor

PLOS One

Journal Requirements:

When submitting your revision, we need you to address these additional requirements.

1. Please ensure that your manuscript meets PLOS ONE's style requirements, including those for file naming. The PLOS ONE style templates can be found at

https://journals.plos.org/plosone/s/file?id=wjVg/PLOSOne_formatting_sample_main_body.pdf and

https://journals.plos.org/plosone/s/file?id=ba62/PLOSOne_formatting_sample_title_authors_affiliations.pdf

2. Please include a link to the protocols.io entry in your Methods section.

3. Thank you for stating the following in the Acknowledgments Section of your manuscript:

“The authors also acknowledge the Doctoral Program in Sciences of the Inter-University Agreement between Universidad Tecnológica de Pereira, Universidad de Caldas, and Universidad del Quindío for its academic support throughout this work, as well as the project “Recubrimientos de hidroxiapatita incorporando iones de cobre por sistemas PAPVD para posibles aplicaciones médicas” (Code 3247) for its financial support.”

We note that you have provided funding information that is not currently declared in your Funding Statement. However, funding information should not appear in the Acknowledgments section or other areas of your manuscript. We will only publish funding information present in the Funding Statement section of the online submission form.

Please remove any funding-related text from the manuscript and let us know how you would like to update your Funding Statement. Currently, your Funding Statement reads as follows:

“The authors also acknowledge the Doctoral Program in Sciences of the Inter-University Agreement between Universidad Tecnológica de Pereira, Universidad de Caldas, and Universidad del Quindío for its academic support throughout this work, as well as the project “Recubrimientos de hidroxiapatita incorporando iones de cobre por sistemas PAPVD para posibles aplicaciones médicas” (Code 3247) for its financial support.”

Please include your amended statements within your cover letter; we will change the online submission form on your behalf.

4. Thank you for stating the following financial disclosure:

“The authors also acknowledge the Doctoral Program in Sciences of the Inter-University Agreement between Universidad Tecnológica de Pereira, Universidad de Caldas, and Universidad del Quindío for its academic support throughout this work, as well as the project “Recubrimientos de hidroxiapatita incorporando iones de cobre por sistemas PAPVD para posibles aplicaciones médicas” (Code 3247) for its financial support.”

Please state what role the funders took in the study.  If the funders had no role, please state: "The funders had no role in study design, data collection and analysis, decision to publish, or preparation of the manuscript."

If this statement is not correct you must amend it as needed.

Please include this amended Role of Funder statement in your cover letter; we will change the online submission form on your behalf.

5. Thank you for stating the following financial disclosure:

“The authors also acknowledge the Doctoral Program in Sciences of the Inter-University Agreement between Universidad Tecnológica de Pereira, Universidad de Caldas, and Universidad del Quindío for its academic support throughout this work, as well as the project “Recubrimientos de hidroxiapatita incorporando iones de cobre por sistemas PAPVD para posibles aplicaciones médicas” (Code 3247) for its financial support.”

We note that one or more of the authors is affiliated with the funding organization, indicating the funder may have had some role in the design, data collection, analysis or preparation of your manuscript for publication; in other words, the funder played an indirect role through the participation of the co-authors. If the funding organization did not play a role in the study design, data collection and analysis, decision to publish, or preparation of the manuscript and only provided financial support in the form of authors' salaries and/or research materials, please do the following:

1. Review your statements relating to the author contributions, and ensure you have specifically and accurately indicated the role(s) that these authors had in your study. These amendments should be made in the online form.

2. Confirm in your cover letter that you agree with the following statement, and we will change the online submission form on your behalf:

“The funder provided support in the form of salaries for authors [insert relevant initials], but did not have any additional role in the study design, data collection and analysis, decision to publish, or preparation of the manuscript. The specific roles of these authors are articulated in the ‘author contributions’ section.

6. Please amend either the title on the online submission form (via Edit Submission) or the title in the manuscript so that they are identical.

7. Please amend the manuscript submission data (via Edit Submission) to include author Leonardo Bohosquez-Santiago.

8. Please amend your authorship list in your manuscript file to include author Leonardo Bohorquez Santiago.

9. Please amend either the abstract on the online submission form (via Edit Submission) or the abstract in the manuscript so that they are identical.

10. Your abstract cannot contain citations. Please only include citations in the body text of the manuscript, and ensure that they remain in ascending numerical order on first mention.

11. Please upload a copy of Figure 5, X, Y and Z, to which you refer in your text on page 14, 17, 18 and 19. If the figure is no longer to be included as part of the submission please remove all reference to it within the text.

12. Please ensure that you refer to Figure 7 and 8 in your text as, if accepted, production will need this reference to link the reader to the figure.

13. We note that Figures 1 - 4 and 6 in your submission contain copyrighted images. All PLOS content is published under the Creative Commons Attribution License (CC BY 4.0), which means that the manuscript, images, and Supporting Information files will be freely available online, and any third party is permitted to access, download, copy, distribute, and use these materials in any way, even commercially, with proper attribution. For more information, see our copyright guidelines: http://journals.plos.org/plosone/s/licenses-and-copyright.

We require you to either (1) present written permission from the copyright holder to publish these figures specifically under the CC BY 4.0 license, or (2) remove the figures from your submission:

1. You may seek permission from the original copyright holder of Figure(s) [#] to publish the content specifically under the CC BY 4.0 license.

We recommend that you contact the original copyright holder with the Content Permission Form (http://journals.plos.org/plosone/s/file?id=7c09/content-permission-form.pdf) and the following text:

“I request permission for the open-access journal PLOS ONE to publish XXX under the Creative Commons Attribution License (CCAL) CC BY 4.0 (http://creativecommons.org/licenses/by/4.0/). Please be aware that this license allows unrestricted use and distribution, even commercially, by third parties. Please reply and provide explicit written permission to publish XXX under a CC BY license and complete the attached form.”

Please upload the completed Content Permission Form or other proof of granted permissions as an "Other" file with your submission.

In the figure caption of the copyrighted figure, please include the following text: “Reprinted from [ref] under a CC BY license, with permission from [name of publisher], original copyright [original copyright year].”

2. If you are unable to obtain permission from the original copyright holder to publish these figures under the CC BY 4.0 license or if the copyright holder’s requirements are incompatible with the CC BY 4.0 license, please either i) remove the figure or ii) supply a replacement figure that complies with the CC BY 4.0 license. Please check copyright information on all replacement figures and update the figure caption with source information. If applicable, please specify in the figure caption text when a figure is similar but not identical to the original image and is therefore for illustrative purposes only.

14. We note you have included a table to which you do not refer in the text of your manuscript. Please ensure that you refer to Table Specifications table in your text; if accepted, production will need this reference to link the reader to the Table.

15. We note you have not yet provided a protocols.io PDF version of your protocol and/or a protocols.io DOI. When you submit your revision, please provide a PDF version of your protocol as generated by protocols.io (the file will have the protocols.io logo in the upper right corner of the first page) as a Supporting Information file. The filename should be S1_file.pdf, and you should enter “S1 File” into the Description field. Any additional protocols should be numbered S2, S3, and so on. Please also follow the instructions for Supporting Information captions [https://journals.plos.org/plosone/s/supporting-information#loc-captions]. The title in the caption should read: “Step-by-step protocol, also available on protocols.io.”

Please assign your protocol a protocols.io DOI, if you have not already done so, and include the following line in the Materials and Methods section of your manuscript: “The protocol described in this peer-reviewed article is published on protocols.io (https://dx.doi.org/10.17504/protocols.io.[...]) and is included for printing purposes as S1 File.” You should also supply the DOI in the Protocols.io DOI field of the submission form when you submit your revision.

If you have not yet uploaded your protocol to protocols.io, you are invited to use the platform’s protocol entry service [https://www.protocols.io/we-enter-protocols] for doing so, at no charge. Through this service, the team at protocols.io will enter your protocol for you and format it in a way that takes advantage of the platform’s features. When submitting your protocol to the protocol entry service please include the customer code PLOS2022 in the Note field and indicate that your protocol is associated with a PLOS ONE Lab Protocol Submission. You should also include the title and manuscript number of your PLOS ONE submission.

16. If the reviewer comments include a recommendation to cite specific previously published works, please review and evaluate these publications to determine whether they are relevant and should be cited. There is no requirement to cite these works unless the editor has indicated otherwise.

[Note: HTML markup is below. Please do not edit.]

Reviewers' comments:

Reviewer's Responses to Questions

**Comments to the Author**

Reviewer #1: Yes

Reviewer #2: Yes

**********

2. Has the protocol been described in sufficient detail?

To answer this question, please click the link to protocols.io in the Materials and Methods section of the manuscript (if a link has been provided) or consult the step-by-step protocol in the Supporting Information files.

Reviewer #1: Yes

Reviewer #2: Partly

**********

3. Does the protocol describe a validated method?

Reviewer #1: Yes

Reviewer #2: Yes

**********

4. If the manuscript contains new data, have the authors made this data fully available?

The PLOS Data policy

Reviewer #1: Yes

Reviewer #2: No

**********

**5. Is the article presented in an intelligible fashion and written in standard English?**

Reviewer #1: **No:**  The manuscript lacks a sufficiently detailed experimental methodology for the copper doping of synthetic hydroxyapatite thin films using plasma-assisted pulsed laser deposition (PA-PLD). The authors should provide comprehensive deposition parameters, including target composition, laser wavelength and fluence, substrate temperature, plasma conditions, chamber pressure, target-to-substrate distance, deposition time, and post-deposition treatment to ensure reproducibility of the work.

Reviewer #2: **No:**  English is fair, the presentation is not good, the restructuring of manuscript is highly recommended.

**********

Reviewer #1: The manuscript lacks a sufficiently detailed experimental methodology for the copper doping of synthetic hydroxyapatite thin films using plasma-assisted pulsed laser deposition (PA-PLD). The authors should provide comprehensive deposition parameters, including target composition, laser wavelength and fluence, substrate temperature, plasma conditions, chamber pressure, target-to-substrate distance, deposition time, and post-deposition treatment to ensure reproducibility of the work.

Reviewer #2: 1. What was the rationale behind selecting copper filaments as the dopant source instead of using pre-mixed copper-containing hydroxyapatite targets?

2. How was the concentration of copper within the deposited HAp-Cu thin films controlled and monitored during the PAPVD process?

3. Did the incorporation of copper filaments influence the ablation behavior or stability of the hydroxyapatite target during repeated laser exposure?

4. What measures were taken to ensure uniform distribution of copper throughout the deposited thin film?

5. Why was silicon selected as the substrate material, and how might the deposition behavior differ on metallic or biomedical implant substrates?

6. Were there any observable changes in crystallinity, phase purity, or preferred orientation of hydroxyapatite after copper doping?

7. How sensitive is the proposed methodology to variations in laser fluence, repetition rate, or chamber pressure?

Was the adhesion strength of the HAp-Cu thin films to the substrate evaluated or qualitatively assessed during the study?

8. Could the proposed PAPVD methodology be extended to incorporate other metallic dopants such as silver, zinc, or magnesium for multifunctional bioactive coatings?

9. What are the main limitations or challenges associated with scaling this copper-doping methodology for larger-area coatings or industrial biomedical applications?

10. Restructure the manuscript in such a way it is easy to read, also present in the order Title, Abstract, Keywords, Introduction, Methods and materials, Results and Discussion, Conclusions, references.

11. Check the grammatical errors and do a thorough proof reading

12. Also no need to use reference in abstract itself, remove that citation

13. State the novelty clearly and highlight the difference between already conducted studies and your study.

**********

what does this mean?). If published, this will include your full peer review and any attached files.

If you choose “no”, your identity will remain anonymous but your review may still be made public.

**Do you want your identity to be public for this peer review?**  For information about this choice, including consent withdrawal, please see our Privacy Policy

Reviewer #1: **Yes:**  J H Markna

Reviewer #2: No

**********

[NOTE: If reviewer comments were submitted as an attachment file, they will be attached to this email and accessible via the submission site. Please log into your account, locate the manuscript record, and check for the action link "View Attachments". If this link does not appear, there are no attachment files.]

To ensure your figures meet our technical requirements, please review our figure guidelines: https://journals.plos.org/plosone/s/figures

You may also use PLOS’s free figure tool, NAAS, to help you prepare publication quality figures: https://journals.plos.org/plosone/s/figures#loc-tools-for-figure-preparation.

NAAS will assess whether your figures meet our technical requirements by comparing each figure against our figure specifications.

# Author comment: Author response to Decision Letter 1

**DOI:** 10.1371/journal.pone.0358441.r002

1 Jul 2026

Subject: Response to Reviewers' Comments on the Author

Below are the comments made by the reviewers, listed in the order they were received via email. In this letter, they are copied exactly as received, including their numbers for better organization. Those requiring a response from the author are addressed under the corresponding number with the subheading "Response" in bold. We thank the reviewers in advance for their comments and observations and hope to resolve any questions that may have arisen.

Reviewers' comments:

1. Does the manuscript report a protocol which is of utility to the research community and adds value to the published literature?

Reviewer #1: Yes

Reviewer #2: Yes

________________________________________

2. Has the protocol been described in sufficient detail?

To answer this question, please click the link to protocols.io in the Materials and Methods section of the manuscript (if a link has been provided) or consult the step-by-step protocol in the Supporting Information files.

The step-by-step protocol should contain sufficient detail for another researcher to be able to reproduce all experiments and analyses.

Reviewer #1: Yes

Reviewer #2: Partly

________________________________________

3. Does the protocol describe a validated method?

The manuscript must demonstrate that the protocol achieves its intended purpose: either by containing appropriate validation data, or referencing at least one original research article in which the protocol was used to generate data.

Reviewer #1: Yes

Reviewer #2: Yes

________________________________________

4. If the manuscript contains new data, have the authors made this data fully available?

The PLOS Data policy requires authors to make all data underlying the findings described in their manuscript fully available without restriction, with rare exception (please refer to the Data Availability Statement in the manuscript PDF file). The data should be provided as part of the manuscript or its supporting information, or deposited to a public repository. For example, in addition to summary statistics, the data points behind means, medians and variance measures should be available. If there are restrictions on publicly sharing data—e.g. participant privacy or use of data from a third party—those must be specified.

Reviewer #1: Yes

Reviewer #2: No

________________________________________

5. Is the article presented in an intelligible fashion and written in standard English?

PLOS ONE does not copyedit accepted manuscripts, so the language in submitted articles must be clear, correct, and unambiguous. Any typographical or grammatical errors should be corrected at revision, so please highlight any specific errors that need correcting in the box below.

Reviewer #1: No: The manuscript lacks a sufficiently detailed experimental methodology for the copper doping of synthetic hydroxyapatite thin films using plasma-assisted pulsed laser deposition (PA-PLD). The authors should provide comprehensive deposition parameters, including target composition, laser wavelength and fluence, substrate temperature, plasma conditions, chamber pressure, target-to-substrate distance, deposition time, and post-deposition treatment to ensure reproducibility of the work.

Response: We thank the reviewer for this valuable comment. The manuscript has been revised to provide a more detailed description of the deposition methodology. Additional information regarding the target composition (sintered hydroxyapatite target modified with high-purity copper strips), laser wavelength (532 nm), pulse duration (6 ns), fluence (30 J·cm⁻²), substrate temperature (room temperature), chamber pressure (6.3 × 10⁻⁵ mbar), target-to-substrate distance (3 cm), target rotation speed (60 rpm), deposition time (5 min), plasma plume conditions, and post-deposition handling has been incorporated into Section 3. These additions improve the reproducibility and transparency of the proposed PAPVD process.

Reviewer #2: No: English is fair, the presentation is not good, the restructuring of manuscript is highly recommended.

________________________________________

6. Review Comments to the Author

Please use the space provided to explain your answers to the questions above. You may also include additional comments for the author, including concerns about dual publication, research ethics, or publication ethics. (Please upload your review as an attachment if it exceeds 20,000 characters)

Reviewer #1: The manuscript lacks a sufficiently detailed experimental methodology for the copper doping of synthetic hydroxyapatite thin films using plasma-assisted pulsed laser deposition (PA-PLD). The authors should provide comprehensive deposition parameters, including target composition, laser wavelength and fluence, substrate temperature, plasma conditions, chamber pressure, target-to-substrate distance, deposition time, and post-deposition treatment to ensure reproducibility of the work.

Response:

We thank the reviewer for this valuable comment. The manuscript has been substantially revised to provide a more comprehensive and reproducible description of the PAPVD deposition methodology.

Specifically, the following experimental details have been incorporated into Section 3 (Doping and Deposition Process):

• Composition of the deposition target, consisting of a sintered hydroxyapatite (HAp) ceramic target modified with high-purity copper strips (99.99%) to enable in situ copper incorporation.

• Nd:YAG laser operating wavelength (532 nm) and pulse duration (6 ns).

• Laser fluence (30 J·cm⁻²), pulse repetition rate (10 Hz), and pulse energy (85 mJ).

• Chamber base pressure (< 5 × 10⁻⁶ mbar) and working pressure (6.3 × 10⁻⁵ mbar).

• Target-to-substrate distance (3 cm).

• Deposition on silicon substrates maintained at room temperature without intentional heating.

• Target rotation speed (60 rpm) during deposition to ensure homogeneous ablation and stable plasma plume generation.

• Deposition time (5 min).

• Post-deposition treatment, consisting of cooling inside the vacuum chamber for 30 min followed by controlled venting with high-purity nitrogen, without additional thermal annealing.

In addition, a summary paragraph containing the principal deposition parameters has been added to facilitate reproducibility and improve the clarity of the experimental procedure

Reviewer #2:

1. What was the rationale behind selecting copper filaments as the dopant source instead of using pre-mixed copper-containing hydroxyapatite targets?

Response: We thank the reviewer for this important question. The use of metallic copper filaments placed on the surface of the hydroxyapatite target was intentionally selected as a flexible and cost-effective strategy for controlling copper incorporation during PAPVD deposition.

Unlike pre-mixed HAp-Cu ceramic targets, the filament-based approach allows the copper content to be modified simply by changing the number and arrangement of the copper strips without requiring the synthesis, pressing, and sintering of a new ceramic target for each composition. This significantly reduces material preparation time and facilitates rapid optimization of deposition conditions.

In addition, the use of metallic copper strips minimizes potential modifications of the hydroxyapatite crystalline structure that may occur during the high-temperature processing required for the fabrication of pre-doped ceramic targets. By maintaining the hydroxyapatite target composition unchanged and introducing copper only during laser ablation, the methodology enables a more direct evaluation of the influence of copper incorporation on the deposited films.

Furthermore, the intermittent co-ablation of hydroxyapatite and copper during target rotation provides a reproducible route for generating Cu-containing coatings while preserving the simplicity and adaptability of the deposition process. This approach was therefore selected as a practical methodology-oriented solution aimed at facilitating reproducibility and experimental flexibility.

2. How was the concentration of copper within the deposited HAp-Cu thin films controlled and monitored during the PAPVD process?

Response: We thank the reviewer for this important question. The copper concentration was controlled through the deliberate modification of the hydroxyapatite target surface using a defined number of high-purity copper strips (2–6 strips), symmetrically distributed over the circular HAp target. This configuration was designed to promote homogeneous and reproducible co-ablation of both hydroxyapatite and copper during laser irradiation.

The circular geometry of the target, combined with continuous target rotation during deposition, ensured that the laser beam periodically interacted with both the HAp surface and the copper strips, promoting a uniform incorporation of copper-containing species into the plasma plume. Consequently, the copper content in the deposited films was controlled by varying the number of copper strips while maintaining identical deposition conditions for all samples.

Copper incorporation was subsequently monitored and verified by Energy Dispersive X-ray Spectroscopy (EDS), which confirmed the presence of copper in the deposited coatings. The systematic increase in the number of copper strips resulted in progressively higher copper incorporation levels, as reported in our complementary characterization study [1].

The copper incorporation level was controlled by varying the number of copper strips placed on the target surface while maintaining all other deposition parameters constant. The symmetric radial arrangement of the copper strips, combined with continuous target rotation during deposition, promoted a reproducible co-ablation process and a homogeneous distribution of copper-containing species within the plasma plume. Copper incorporation into the deposited films was subsequently verified by EDS analysis.

3. Did the incorporation of copper filaments influence the ablation behavior or stability of the hydroxyapatite target during repeated laser exposure?

Response: We thank the reviewer for this insightful question. The incorporation of copper filaments did not produce observable instabilities in the ablation process nor compromise the structural integrity of the hydroxyapatite target during repeated laser irradiation cycles.

Although copper and hydroxyapatite exhibit different thermal conductivities, optical absorption coefficients, and laser–material interaction characteristics, the symmetric distribution of the copper strips over the target surface, together with continuous target rotation and rastering, promoted a homogeneous ablation process throughout deposition.

During the PAPVD experiments, no significant fluctuations in plasma plume formation, pressure stability, or deposition behavior were observed when compared with the undoped hydroxyapatite target. Furthermore, no evidence of target cracking, delamination of the copper strips, or localized damage that could adversely affect the deposition process was detected.

These observations indicate that the incorporation of copper filaments is compatible with stable and reproducible PAPVD operation under the selected experimental conditions.

4. What measures were taken to ensure uniform distribution of copper throughout the deposited thin film?

Response: We thank the reviewer for this important question. Several experimental measures were implemented to promote a homogeneous incorporation of copper throughout the deposited HAp-Cu films.

First, the copper strips were symmetrically distributed over the surface of the circular hydroxyapatite target. This configuration was specifically designed to favor the homogeneous co-ablation of hydroxyapatite and copper during laser irradiation, minimizing localized compositional variations associated with preferential ablation.

Second, continuous target rotation and rastering were employed during deposition, ensuring periodic interaction of the laser beam with both the hydroxyapatite surface and the copper strips. This dynamic ablation strategy contributed to the generation of a stable plasma plume containing species from both materials and reduced the likelihood of localized copper enrichment.

Finally, critical deposition parameters, including laser fluence, chamber pressure, target-to-substrate distance, and deposition time, were maintained constant throughout all experiments to ensure process stability and reproducibility.

The effectiveness of these measures was supported by EDS characterization, which confirmed the successful incorporation of copper within the deposited coatings.

5. Why was silicon selected as the substrate material, and how might the deposition behavior differ on metallic or biomedical implant substrates?

Response: We thank the reviewer for this important question. Silicon was selected as the substrate material because it provides a well-defined, smooth, chemically stable, and reproducible surface that is widely used in thin-film deposition studies. The use of silicon minimizes the influence of substrate-related variables and facilitates the characterization of the deposited coatings by techniques such as SEM, EDS, XRD, AFM, and profilometry.

In the present work, the primary objective was to establish and validate a reproducible methodology for the fabrication of copper-doped hydroxyapatite thin films by PAPVD. Therefore, silicon was employed as a model substrate to focus on the deposition process itself and the incorporation of copper into the hydroxyapatite matrix.

We recognize that deposition behavior may differ on metallic substrates commonly used in biomedical applications, such as titanium and titanium alloys. Factors including surface roughness, thermal conductivity, surface energy, native oxide layers, and differences in thermal expansion coefficients may influence nucleation, film adhesion, growth kinetics, residual stress development, and coating morphology. Nevertheless, the fundamental co-ablation and copper-incorporation strategy described in this work is expected to remain applicable to metallic implant materials, although further optimization of deposition parameters may be required for each specific substrate.

This clarification has been incorporated into the revised manuscript.

6. Were there any observable changes in crystallinity, phase purity, or preferred orientation of hydroxyapatite after copper doping?

Response: We thank the reviewer for this important question. XRD analysis revealed that all deposited coatings maintained the characteristic crystalline structure of hydroxyapatite, and no additional crystalline phases associated with copper, copper oxides, or other calcium phosphate compounds were detected. These results indicate that the phase purity of hydroxyapatite was preserved after copper incorporation.

Although no significant shifts were observed in the position of the HAp (002) diffraction peak, variations in the full width at half maximum (FWHM) and crystallite size were detected among the Cu-containing samples. These changes suggest that copper incorporation influenced the crystalline quality of the coatings without inducing structural phase transformations.

Furthermore, the absence of significant changes in the (002) peak position indicates that copper was not substantially incorporated into the hydroxyapatite crystal lattice. Instead, the combined XRD and XPS results suggest that copper was mainly present at the coating surface or in an amorphous form associated with the hydroxyapatite matrix.

Therefore, copper incorporation modified the crystallinity-related parameters of the coatings while preserving the hydroxyapatite phase composition and without evidence of significant changes in preferred crystallographic orientation.

7. How sensitive is the proposed methodology to variations in laser fluence, repetition rate, or chamber pressure?

Response: We thank the reviewer for this valuable question. The primary objective of the present study was to demonstrate the feasibility of copper incorporation into hydroxyapatite thin films using the proposed PAPVD methodology rather than to perform a systematic optimization of the deposition parameters. Therefore, laser fluence, repetition rate, and chamber pressure were maintained constant throughout all experiments.

Nevertheless, these parameters are known to strongly influence the ablation rate, plasma plume dynamics, energy transfer, and ultimately the composition and morphology of the deposited coatings. Consequently, variations in these parameters are expected to affect the deposition process and the resulting film characteristics. A detailed parametric study evaluating the sensitivity of the methodology to these variables is considered an important topic for future work.

Was the adhesion strength of the HAp-Cu thin films to the substrate evaluated or qualitatively assessed during the study?

Response: We thank the reviewer for this important observation. Quantitative adhesion measurements, such as scratch testing, pull-off testing, or nanoindentation-based adhesion analysis, were not performed in the present study, as the primary objective was to establish and validate the PAPVD methodology for the fabrication of Cu-doped hydroxyapatite thin films.

Nevertheless, qualitative observations were carried out during sample handling and subsequent characterization by SEM, EDS, and XRD. No visible film delamination, peeling, cracking, or spontaneous detachment from the silicon substrates was observed during these procedures. Furthermore, SEM analysis revealed continuous and relatively dense coatings, suggesting adequate film adherence for characterization purposes.

We acknowledge that quantitative adhesion testing would provide valuable information regarding the mechanical integrity of the coatings and therefore represents an important topic for future work, particularly for biomedical coating applications.

8. Could the proposed PAPVD methodology be extended to incorporate other metallic dopants such as silver, zinc, or magnesium for multifunctional bioactive coatings?

Response: We thank the reviewer for this interesting question. In principle, the proposed PAPVD methodology is not restricted to copper and could be extended to other metallic dopants such as silver, zinc, magnesium, or combinations thereof. The strategy employed in this work relies on the physical incorporation of metallic sources onto the hydroxyapatite target surface, allowing their simultaneous ablation with hydroxyapatite during the deposition process.

However, the successful implementation of alternative dopants would require optimization of the deposition parameters because each material exhibits different laser–matter interaction characteristics, including optical absorption, ablation threshold, thermal conductivity, melting temperature, vaporization behavior, and plasma plume dynamics. These factors may influence dopant incorporation efficiency, film composition, and coating morphology.

Nevertheless, the general methodology described in this work provides a flexible platform that could potentially be adapted for the fabrication of multifunctional hydroxyapatite-based coatings incorporating antibacterial, osteogenic, angiogenic, or other therapeutic metallic species. The exploration of alternative dopants represents an interesting direction for future studies.

9. What are the main limitations or challenges associated with scaling this copper-doping methodology for larger-area coatings or industrial biomedical applications?

Response: We thank the reviewer for this important question. Although the proposed PAPVD methodology proved effective for the fabrication of Cu-doped hydroxyapatite thin films at laboratory scale, several challenges must be addressed before large-scale implementation.

First, maintaining a homogeneous copper distribution over larger substrate areas may become increasingly difficult due to the localized nature of laser ablation and the use of discrete copper strips as the dopant source. Larger-area coatings would require optimization of the target geometry, scanning strategy, and substrate motion to ensure compositional uniformity.

Second, prolonged operation may lead to non-uniform target erosion and variations in the relative ablation rates of hydroxyapatite and copper, potentially affecting the reproducibility of film composition during extended deposition campaigns.

Third, industrial-scale production would require improvements in deposition throughput, including larger targets, automated target replacement systems, higher-power laser sources, or multi-beam configurations to increase coating area and productivity.

From a biomedical perspective, additional studies would be necessary to evaluate coating adhesion, mechanical durability, long-term stability, corrosion resistance, biocompatibility, and regulatory compliance on clinically relevant substrates such as titanium and titanium alloys.

Therefore, while the proposed methodology demonstrates considerable flexibility and reproducibility at the laboratory scale, further engineering development and application-specific optimization would be required for large-scale industrial implementation.

10. Restructure the manuscript in such a way it is easy to read, also present in the order Title, Abstract, Keywords, Introduction, Methods and materials, Results and Discussion, Conclusions, references.

Response: We thank the reviewer for this valuable suggestion. The manuscript has been extensively reorganized to improve its readability, logical flow, and overall clarity.

In the revised version, the manuscript now follows the recommended structure:

• Title

• Abstract

• Keywords

• Introduction

• Materials and Methods

• Results and Discussion

• Conclusions

• References

To address this comment, the previous methodology-centered organization was restructured into a conventional scientific article format. All experimental procedures, including hydroxyapatite target preparation, substrate cleaning, PAPVD experimental setup, copper doping strategy, deposition protocol, and post-deposition handling, have been consolidated within the Materials and Methods section. Likewise, the validation of the proposed methodology through visual inspection, SEM, EDS, and XRD analyses has been integrated into the Results and Discussion section.

In addition, a dedicated Conclusions section has been incorporated to summarize the main methodological contributions and key findings of the study. Section numbering, subsection organization, and figure placement were also revised to improve the continuity of the manuscript and facilitate navigation for readers.

These modifications were implemented to enhance the overall accessibility, readability, and scientific presentation of the manuscript.

11. Check the grammatical errors and do a thorough proof reading

Response: We thank the reviewer for this observation. The manuscript has been carefully revised and thoroughly proofread to correct grammatical, typographical, punctuation, and formatting errors.

In the revised version, we reviewed sentence structure, terminology consistency, figure citations, section numbering, unit notation, spacing, and overall readability. Particular attention was given to improving the clarity of the Materials and Methods and Results and Discussion sections, ensuring that the experimental procedure and methodological validation are presented in a clearer and more coherent manner.

These revisions were made to improve the scientific readability and overall quality of the manuscript.

12. Also, no need to use reference in abstract itself, remove that citation

Response: We thank the reviewer for this observation. The abstract has been revised accordingly, and the citation previously included in the abstract has been removed. References are now cited exclusively within the main body of the manuscript, in accordance with the journal guidelines. This modification improves the readability of the abstract and ensures consistency with the recommended manuscript format.

13. State the novelty clearly and highlight the difference between already conducted studies and your study.

Response: We thank the reviewer for this valuable observation, which has allowed us to further clarify the contribution and novelty of the present work.

The principal novelty of this study lies in the implementation of an experimental PAPVD methodology for the fabrication of Cu-doped hydroxyapatite thin films through the direct incorporation of metallic copper strips onto the surface of a hydroxyapatite target. During laser irradiation, this configuration promotes the simultaneous ablation of hydroxyapatite and copper, enabling in situ copper incorporation during film growth without requiring the prior synthesis of Cu-containing hydroxyapatite powders or the fabrication of pre-doped ceramic targets.

In contrast to many previously reported approaches, where metallic ions are incorporated into hydroxyapatite through chemical synthesis routes, ion-substitution processes, or the preparation of pre-doped targets before deposition, the methodology proposed here introduces a direct and flexible co-ablation strategy in which the dopant source is incorporated physically into the target assembly immediately prior to deposition.

An additional advantage of this approach is that the copper incorporation level can be progressively modified by varying the number of copper strips placed on the hydroxyapatite target while maintaining the remaining deposition parameters unchanged. This provides a simple route for controlling dopant incorporation without the need to fabricate multiple targets with different compositions.

Furthermore, while our previous publication focused on the physicochemical, structural, surface, and biological characterization of the resulting HAp-Cu coatings [1], the objective of the present manuscript is to provide a detailed, reproducible, and transferable methodology describing target preparation, dopant incorporation, PAPVD configuration, deposition conditions, and validation procedures.

Therefore, the contribution of the present work is not only the demonstration of a viable copper incorporation strategy for hydroxyapatite thin films, but also the establishment of a reproducible PAPVD methodology that may serve as a platform for future studies involving metallic-doped hydroxyapatite coatings and multifunctional bioactive thin films.

________________________________________

This letter addresses all the comments made by the reviewers and clarifies that the requested adjustments to the main document will be made according to the feedback provided and in accordance with the responses given in this letter. We again thank the reviewers for their valuable contributions to improving the quality of the article. We look forward to your feedback.

Attachment Submitted filename: Letter1 _LBohorquez_Singed.pdf

# Peer review: Decision Letter 1

**DOI:** 10.1371/journal.pone.0358441.r003

**Authors:** Mehnath Sivaraj

28 Jul 2026

Dear Dr. Bohorquez Santiago,

Thank you for submitting your manuscript to PLOS ONE. After careful consideration, we feel that it has merit but does not fully meet PLOS ONE’s publication criteria as it currently stands. Therefore, we invite you to submit a revised version of the manuscript that addresses the points raised during the review process.

Please submit your revised manuscript by Sep 11 2026 11:59PM. If you will need more time than this to complete your revisions, please reply to this message or contact the journal office at plosone@plos.org. When you're ready to submit your revision, log on to https://www.editorialmanager.com/pone/ and select the 'Submissions Needing Revision' folder to locate your manuscript file.

- A letter that responds to each point raised by the academic editor and reviewer(s). You should upload this letter as a separate file labeled 'Response to Reviewers'.
- A marked-up copy of your manuscript that highlights changes made to the original version. You should upload this as a separate file labeled 'Revised Manuscript with Track Changes'.
- An unmarked version of your revised paper without tracked changes. You should upload this as a separate file labeled 'Manuscript'.

If you would like to make changes to your financial disclosure, please include your updated statement in your cover letter. Guidelines for resubmitting your figure files are available below the reviewer comments at the end of this letter.

If applicable, we recommend that you deposit your laboratory protocols in protocols.io to enhance the reproducibility of your results. Protocols.io assigns your protocol its own identifier (DOI) so that it can be cited independently in the future. For instructions see: https://journals.plos.org/plosone/s/submission-guidelines#loc-laboratory-protocols. Additionally, PLOS ONE offers an option for publishing peer-reviewed Lab Protocol articles, which describe protocols hosted on protocols.io. Read more information on sharing protocols at https://plos.org/protocols?utm_medium=editorial-email&utm_source=authorletters&utm_campaign=protocols.

As the corresponding author, your ORCID iD is verified in the submission system and will appear in the published article. PLOS supports the use of ORCID, and we encourage all coauthors to register for an ORCID iD and use it as well. Please encourage your coauthors to verify their ORCID iD within the submission system before final acceptance, as unverified ORCID iDs will not appear in the published article. *Only*  the individual author can complete the verification step; PLOS staff *cannot*  verify ORCID iDs on behalf of authors.

We look forward to receiving your revised manuscript.

Kind regards,

Mehnath Sivaraj

Academic Editor

PLOS One

Journal Requirements:

1. If the reviewer comments include a recommendation to cite specific previously published works, please review and evaluate these publications to determine whether they are relevant and should be cited. There is no requirement to cite these works unless the editor has indicated otherwise.

2. Please review your reference list to ensure that it is complete and correct. If you have cited papers that have been retracted, please include the rationale for doing so in the manuscript text, or remove these references and replace them with relevant current references. Any changes to the reference list should be mentioned in the rebuttal letter that accompanies your revised manuscript. If you need to cite a retracted article, indicate the article’s retracted status in the References list and also include a citation and full reference for the retraction notice.

[Note: HTML markup is below. Please do not edit.]

Reviewer's Responses to Questions

**Comments to the Author**

Reviewer #1: Yes

Reviewer #2: Yes

**********

2. Has the protocol been described in sufficient detail?

To answer this question, please click the link to protocols.io in the Materials and Methods section of the manuscript (if a link has been provided) or consult the step-by-step protocol in the Supporting Information files.

Reviewer #1: Yes

Reviewer #2: Yes

**********

3. Does the protocol describe a validated method?

Reviewer #1: Yes

Reviewer #2: Yes

**********

4. If the manuscript contains new data, have the authors made this data fully available?

The PLOS Data policy

Reviewer #1: Yes

Reviewer #2: Yes

**********

**5. Is the article presented in an intelligible fashion and written in standard English?**

Reviewer #1: Yes

Reviewer #2: Yes

**********

Reviewer #1: The manuscript contains comprehensive experimental data with full methodological justification, including synthesis, deposition, and characterization of copper-doped hydroxyapatite thin films, providing sufficient evidence to support the conclusions and warrant publication.

Reviewer #2: Figure 6, Figure 7 and Figure 8 image qualities are very poor, Improve Resolution or replace it. The other comments have been addressed well.

**********

what does this mean?). If published, this will include your full peer review and any attached files.

If you choose “no”, your identity will remain anonymous but your review may still be made public.

**Do you want your identity to be public for this peer review?**  For information about this choice, including consent withdrawal, please see our Privacy Policy

Reviewer #1: **Yes:**  J H Markna

Reviewer #2: No

**********

[NOTE: If reviewer comments were submitted as an attachment file, they will be attached to this email and accessible via the submission site. Please log into your account, locate the manuscript record, and check for the action link "View Attachments". If this link does not appear, there are no attachment files.]

To ensure your figures meet our technical requirements, please review our figure guidelines: https://journals.plos.org/plosone/s/figures

You may also use PLOS’s free figure tool, NAAS, to help you prepare publication quality figures: https://journals.plos.org/plosone/s/figures#loc-tools-for-figure-preparation.

NAAS will assess whether your figures meet our technical requirements by comparing each figure against our figure specifications.

# Author comment: Author response to Decision Letter 2

**DOI:** 10.1371/journal.pone.0358441.r004

3 Aug 2026

Response to Reviewers

Manuscript ID: PONE-D-26-06266R1

Manuscript title: Detailed method for copper doping of synthetic hydroxyapatite thin films by plasma-assisted pulsed laser deposition

Dear Dr. Mehnath Sivaraj and Reviewers,

We sincerely thank the Academic Editor and the Reviewers for their careful evaluation of our revised manuscript and for their constructive comments. We greatly appreciate the positive assessment of the scientific and methodological quality of our work.

In response to the remaining comment, Figures 6, 7, and 8 have been replaced with improved, high-resolution versions. The changes made in the revised manuscript are described below. The corresponding modifications are shown in the marked-up version of the manuscript.

Reviewer #1

Comment: “The manuscript contains comprehensive experimental data with full methodological justification, including synthesis, deposition, and characterization of copper-doped hydroxyapatite thin films, providing sufficient evidence to support the conclusions and warrant publication.”

Response: We sincerely thank the Reviewer for the positive assessment of our manuscript and for recognizing the methodological detail, experimental validation, and scientific relevance of the presented protocol. We appreciate the Reviewer’s time and careful evaluation of our work.

Reviewer #2

Comment: “Figure 6, Figure 7 and Figure 8 image qualities are very poor, Improve Resolution or replace it. The other comments have been addressed well.”

Response: We thank the Reviewer for this important observation. In response to the comment, Figures 6, 7, and 8 have been replaced with improved, high-resolution versions. The revised figures were prepared from the original source files to enhance their sharpness, resolution, and overall readability while preserving the original scientific information and interpretation.

The updated figures have been incorporated into both the marked-up and clean versions of the revised manuscript. In addition, Figures 6, 7, and 8 have been prepared as separate publication-quality files and will be uploaded individually to the submission system, replacing the previously submitted versions.

Changes in the revised manuscript:

• Figure 6 was replaced with a higher-resolution version.

• Figure 7 was replaced with a higher-resolution version.

• Figure 8 was replaced with a higher-resolution version.

No changes were made to the scientific results, figure interpretation, or conclusions associated with these figures.

Location in the revised manuscript: Figure 6: page 20; Figure 7: page 22; Figure 8: page 24.

We thank the Reviewer again for the helpful recommendation, which has improved the visual quality and presentation of the manuscript.

Journal Requirements

The reference list has been reviewed to ensure its completeness, accuracy, and correspondence with the citations included in the manuscript. No references were added solely at the request of the Reviewers, as no specific publications were recommended for citation.

We hope that the revised manuscript and the improved figures satisfactorily address the remaining concern. We sincerely thank the Academic Editor and the Reviewers for their valuable time and consideration.

Attachment Submitted filename: Response to Reviewers.pdf

# Peer review: Decision Letter 2

**DOI:** 10.1371/journal.pone.0358441.r005

**Authors:** Wenguo Cui

12 Aug 2026

Dear Dr. Bohorquez Santiago,

Thank you for submitting your manuscript to PLOS ONE. After careful consideration, we feel that it has merit but does not fully meet PLOS ONE’s publication criteria as it currently stands. Therefore, we invite you to submit a revised version of the manuscript that addresses the points raised during the review process.

Please submit your revised manuscript by Sep 26 2026 11:59PM. If you will need more time than this to complete your revisions, please reply to this message or contact the journal office at plosone@plos.org. When you're ready to submit your revision, log on to https://www.editorialmanager.com/pone/ and select the 'Submissions Needing Revision' folder to locate your manuscript file.

- A letter that responds to each point raised by the academic editor and reviewer(s). You should upload this letter as a separate file labeled 'Response to Reviewers'.
- A marked-up copy of your manuscript that highlights changes made to the original version. You should upload this as a separate file labeled 'Revised Manuscript with Track Changes'.
- An unmarked version of your revised paper without tracked changes. You should upload this as a separate file labeled 'Manuscript'.

If you would like to make changes to your financial disclosure, please include your updated statement in your cover letter. Guidelines for resubmitting your figure files are available below the reviewer comments at the end of this letter.

If applicable, we recommend that you deposit your laboratory protocols in protocols.io to enhance the reproducibility of your results. Protocols.io assigns your protocol its own identifier (DOI) so that it can be cited independently in the future. For instructions see: https://journals.plos.org/plosone/s/submission-guidelines#loc-laboratory-protocols. Additionally, PLOS ONE offers an option for publishing peer-reviewed Lab Protocol articles, which describe protocols hosted on protocols.io. Read more information on sharing protocols at https://plos.org/protocols?utm_medium=editorial-email&utm_source=authorletters&utm_campaign=protocols.

As the corresponding author, your ORCID iD is verified in the submission system and will appear in the published article. PLOS supports the use of ORCID, and we encourage all coauthors to register for an ORCID iD and use it as well. Please encourage your coauthors to verify their ORCID iD within the submission system before final acceptance, as unverified ORCID iDs will not appear in the published article. *Only*  the individual author can complete the verification step; PLOS staff *cannot*  verify ORCID iDs on behalf of authors.

We look forward to receiving your revised manuscript.

Kind regards,

Wenguo Cui, Ph.D

Academic Editor

PLOS One

Journal Requirements:

If the reviewer comments include a recommendation to cite specific previously published works, please review and evaluate these publications to determine whether they are relevant and should be cited. There is no requirement to cite these works unless the editor has indicated otherwise.

Please review your reference list to ensure that it is complete and correct. If you have cited papers that have been retracted, please include the rationale for doing so in the manuscript text, or remove these references and replace them with relevant current references. Any changes to the reference list should be mentioned in the rebuttal letter that accompanies your revised manuscript. If you need to cite a retracted article, indicate the article’s retracted status in the References list and also include a citation and full reference for the retraction notice.

[Note: HTML markup is below. Please do not edit.]

Reviewers' comments:

Reviewer's Responses to Questions

**Comments to the Author**

Reviewer #1: Yes

Reviewer #2: Yes

**********

2. Has the protocol been described in sufficient detail?

To answer this question, please click the link to protocols.io in the Materials and Methods section of the manuscript (if a link has been provided) or consult the step-by-step protocol in the Supporting Information files.

Reviewer #1: Yes

Reviewer #2: Yes

**********

3. Does the protocol describe a validated method?

Reviewer #1: Yes

Reviewer #2: Yes

**********

4. If the manuscript contains new data, have the authors made this data fully available?

The PLOS Data policy

Reviewer #1: Yes

Reviewer #2: Yes

**********

**5. Is the article presented in an intelligible fashion and written in standard English?**

Reviewer #1: Yes

Reviewer #2: Yes

**********

Reviewer #1: 1. Inconsistent terminology — "Plasma-Assisted Pulsed Laser Deposition (PAPVD)" in abstract vs "Plasma-Assisted Physical Vapor Deposition (PAPVD)" in introduction. PAPVD = Plasma-Assisted Pulsed Laser Deposition (a variant of PLD, not PVD)

2. Ca/P ratio (1.67) mentioned but no verification method provided. Add XRD or FTIR confirmation of HAp phase after synthesis

3. Cooling rate missing — "allowed to cool naturally" should specify ~3-5°C/min for 1. Line 357.

4. Laser spot size stated as "~0.6 mm diameter" — INCONSISTENT with Line 236! Use single value.

5. CRITICAL ERROR — Fluence calculation wrong. 85 mJ / 0.028 mm² = 3.03 J/cm², NOT 30 J/cm². Correct calculation or verify measurement

6. No target density measurement — "dense targets" should be quantified: ~95% of theoretical density

7. No film thickness measurement — essential for reproducibility (add cross-sectional SEM data)

8. Give complage XRD patterns — add Miller indices (hkl) above peaks

9. Add a flowchart summarizing the entire experimental procedure from HAp synthesis to thin film characterization

10. Add a table of deposition parameters for quick reference (laser wavelength, fluence, repetition rate, pressure, distance, time, etc.)

11. Add a quantitative comparison table showing Cu content (EDS/ICP) for HApCu-2 through HApCu-6 samples

12. Add cross-sectional SEM images to show film thickness and uniformity

To the Authors:

I recommend citing the following three articles from your own research group to strengthen the methodological validation, theoretical context, and structure-property correlation in your manuscript:

https://doi.org/10.1007/s11664-026-12982-z

https://doi.org/10.1016/j.asej.2016.04.015

These additions will improve the scientific rigor and contextual depth of your work.

Reviewer #2: All my questions have been addressed satisfactorily. Congratulations to the authors. The article may be accepted in its current form.

**********

what does this mean?). If published, this will include your full peer review and any attached files.

If you choose “no”, your identity will remain anonymous but your review may still be made public.

**Do you want your identity to be public for this peer review?**  For information about this choice, including consent withdrawal, please see our Privacy Policy

Reviewer #1: **Yes:**  J H Markna

Reviewer #2: No

**********

[NOTE: If reviewer comments were submitted as an attachment file, they will be attached to this email and accessible via the submission site. Please log into your account, locate the manuscript record, and check for the action link "View Attachments". If this link does not appear, there are no attachment files.]

To ensure your figures meet our technical requirements, please review our figure guidelines: https://journals.plos.org/plosone/s/figures

You may also use PLOS’s free figure tool, NAAS, to help you prepare publication quality figures: https://journals.plos.org/plosone/s/figures#loc-tools-for-figure-preparation.

NAAS will assess whether your figures meet our technical requirements by comparing each figure against our figure specifications.

# Author comment: Author response to Decision Letter 3

**DOI:** 10.1371/journal.pone.0358441.r006

30 Aug 2026

Subject: answer to Review Comments to the Author.

We appreciate the comments provided. To address them, we will respond to each reviewer's observations point by point. This approach will clarify the issues raised and the scope of the responses.

Response to Reviewer #1

We sincerely thank Reviewer #1 for the careful evaluation of our manuscript and for the constructive comments. We have revised the manuscript accordingly. Our point-by-point responses are provided below.

- Inconsistent terminology — "Plasma-Assisted Pulsed Laser Deposition (PAPVD)" in abstract vs "Plasma-Assisted Physical Vapor Deposition (PAPVD)" in introduction. PAPVD = Plasma-Assisted Pulsed Laser Deposition (a variant of PLD, not PVD)

Response:

We thank the Reviewer for identifying this inconsistency. We agree that the terminology should be used consistently throughout the manuscript. Accordingly, all instances in which PAPVD was incorrectly expanded as “Plasma-Assisted Physical Vapor Deposition” have been corrected to “Plasma-Assisted Pulsed Laser Deposition.” The terminology has been standardized in the Keywords, Introduction, Specifications Table, Materials and Methods, Conclusions, and method title. These corrections are terminological only and do not affect the experimental methodology, results, or conclusions of the manuscript.

- Ca/P ratio (1.67) mentioned but no verification method provided. Add XRD or FTIR confirmation of HAp phase after synthesis

Response:

We thank the Reviewer for this important observation. We have clarified that the Ca/P molar ratio of 1.67 refers to the stoichiometric precursor ratio selected for hydroxyapatite synthesis and not to an experimentally measured Ca/P ratio of the final material. Direct XRD or FTIR characterization of the as-synthesized powder was not performed as a separate measurement in the present methodological study. Therefore, to avoid overstating the available evidence, the revised manuscript now states that the hydroxyapatite phase obtained using this synthesis route and subsequently deposited as thin films was experimentally verified in our previously published complementary study [1]. In that work, XRD confirmed the characteristic crystalline HAp phase and the diffraction patterns were indexed according to PDF No. 00-009-0432; FTIR also showed the characteristic phosphate vibrational bands at approximately 1024 and 960 cm⁻¹. This clarification has been added to Section 2.1.1 of the revised manuscript.

- Cooling rate missing — "allowed to cool naturally" should specify ~3-5°C/min for 1. Line 357.

Response:

We thank the Reviewer for this observation. The cooling procedure has been clarified in the revised manuscript. Following the 2 h dwell at 1150 °C, the furnace was switched off and the HAp targets were allowed to cool passively inside the closed furnace to room temperature over approximately 16 h. Because this cooling stage was not actively programmed, the cooling rate was not constant. Based on the overall temperature decrease and cooling time, the average cooling rate was approximately 1.2 °C/min. This information has been added to Section 2.1.2 to improve reproducibility without introducing an unsupported programmed cooling rate.

- Laser spot size stated as "~0.6 mm diameter" — INCONSISTENT with Line 236! Use single value.

Response:

We thank the Reviewer for identifying this inconsistency. The laser spot size has been standardized throughout the manuscript to approximately 0.6 mm in diameter. The value previously reported as 0.7 mm in the Experimental Setup section has been corrected accordingly to ensure consistency with the deposition parameters and the reported laser fluence.

- CRITICAL ERROR — Fluence calculation wrong. 85 mJ / 0.028 mm² = 3.03 J/cm², NOT 30 J/cm². Correct calculation or verify measurement

Response:

We thank the Reviewer for identifying this inconsistency. After carefully verifying the laser parameters and calculation, we found that the error was in the reported irradiated spot area rather than in the laser fluence.

For a laser spot diameter of approximately 0.60 mm, the corresponding irradiated area is approximately 0.283 mm² (2.83 × 10⁻³ cm²), rather than 0.028 mm² as incorrectly stated in the previous version of the manuscript. Using an energy per pulse of 85 mJ, the resulting fluence is therefore:

F = 0.085 J / 2.83 × 10⁻³ cm² ≈ 30 J/cm².

Accordingly, the spot area has been corrected from 0.028 mm² to 0.283 mm², and the laser spot diameter has been standardized to approximately 0.60 mm throughout the manuscript. The reported on-target fluence of approximately 30 J/cm² therefore remains unchanged.

- No target density measurement — "dense targets" should be quantified: ~95% of theoretical density

Response:

We thank the Reviewer for this important observation. The density of the sintered HAp targets was not experimentally measured; therefore, reporting a numerical relative density (for example, ~95% of the theoretical density) would not be supported by the available data. To avoid an unsupported quantitative claim, the term “dense” has been removed when describing the targets. They are now described as mechanically robust ceramic targets with sufficient structural integrity for PLD, which reflects the experimentally observed characteristics relevant to laser ablation. No numerical density value has been introduced.}

- No film thickness measurement — essential for reproducibility (add cross-sectional SEM data)

Response:

We thank the Reviewer for this important observation and agree that film thickness is a relevant parameter for the quantitative characterization of thin-film deposition. However, cross-sectional SEM and profilometric thickness measurements were not performed during the original experimental campaign, and the original coated specimens are no longer available for retrospective measurement. We therefore consider that providing an estimated thickness would be scientifically inappropriate.

To address this limitation transparently, the revised manuscript removes any statement that could imply that cross-sectional SEM measurements were available and explicitly adds film-thickness quantification to the Limitations section. The manuscript now states that no numerical film-thickness value is reported and recommends cross-sectional SEM or profilometry in future applications of the protocol. In addition, the experimentally controlled deposition parameters required to reproduce the process are reported in detail.

- Give complage XRD patterns — add Miller indices (hkl) above peaks

Response:

We thank the Reviewer for this valuable suggestion and agree that complete diffraction patterns with Miller-index assignments are important for phase identification. The complete XRD patterns corresponding to the HAp, HApCu-2, HApCu-3, HApCu-4, HApCu-5, and HApCu-6 coatings, including phase identification and Miller indices (hkl), were previously published and discussed in our complementary study [1] and cannot be reproduced in the present manuscript.

To address the Reviewer’s concern without duplicating the previously published figure, we revised the XRD section to clearly distinguish the information presented in the current manuscript from that available in Ref. [1]. The section is now entitled “XRD-Derived Structural Parameters,” and Figure 9 presents only the FWHM values and crystallite sizes derived from the (002) reflection. The text and figure caption explicitly direct readers to Ref. [1] for the complete diffraction profiles, phase identification, and Miller-index assignments. We also removed statements that could incorrectly imply that Figure 9 itself displays the complete XRD patterns.

- Add a flowchart summarizing the entire experimental procedure from HAp synthesis to thin film characterization

Response:

We thank the Reviewer for this valuable suggestion. To improve the clarity and readability of the manuscript, a new flowchart has been added summarizing the complete experimental procedure, from hydroxyapatite synthesis and target fabrication to copper-assisted deposition, post-deposition handling, and thin-film characterization.

This new schematic overview has been incorporated as Figure 1 and is referenced at the end of the Introduction. It provides a concise visual summary of the full workflow followed in the present methodological study.

- Add a table of deposition parameters for quick reference (laser wavelength, fluence, repetition rate, pressure, distance, time, etc.)

Response:

We thank the Reviewer for this helpful suggestion. A new table has been added to the Materials and Methods section to provide a concise summary of the principal PAPVD deposition parameters used throughout the experiments.

The table includes the laser wavelength, pulse duration, energy per pulse, laser spot size, fluence, repetition rate, chamber pressure, target-to-substrate distance, substrate temperature, target rotation speed, deposition time, and post-deposition conditions.

This addition provides a quick reference to the experimental conditions and improves the clarity and reproducibility of the described methodology.

- Add a quantitative comparison table showing Cu content (EDS/ICP) for HApCu-2 through HApCu-6 samples

Response:

We thank the reviewer for this important suggestion. To address this suggestion, a table showing the weight percentage of elements present in the deposited film obtained through SEM characterization in this research is included. This table allows for the quantitative identification of the amount of copper deposited in relation to the Ca/P ratio in the hydroxyapatite film, bearing in mind that most of this copper is presumed to be on the surface.

- Add cross-sectional SEM images to show film thickness and uniformity

Response:

We thank the Reviewer for this important suggestion. We agree that cross-sectional SEM imaging would provide valuable quantitative information regarding film thickness and through-thickness uniformity. However, cross-sectional SEM images were not acquired during the original experimental campaign, and the original coated specimens are no longer available for retrospective cross-sectional preparation and analysis. Therefore, it is not possible to provide reliable cross-sectional SEM images or film-thickness measurements at this stage without repeating the experimental deposition procedure.

To address this limitation transparently, the revised manuscript has been modified in two ways. First, the SEM section now explicitly states that the available SEM observations correspond to surface-view images and therefore cannot be used to determine film thickness or cross-sectional uniformity. Second, the Limitations section has been expanded to explicitly acknowledge that neither film thickness nor through-thickness uniformity was directly evaluated.

We have deliberately avoided providing an estimated film thickness or inferring cross-sectional uniformity from the available surface SEM images, as such conclusions would not be adequately supported by the experimental data. The revised manuscript now recommends cross-sectional SEM and/or profilometric measurements in future applications of the protocol to determine film thickness, evaluate cross-sectional uniformity, and establish the deposition rate.

__________________________________________________________________________________________________________________________

August 30 / 2026

We appreciate your comments regarding the improvement of our manuscript. In response to your feedback, we noted the absence of the supplementary information; we have now included this material in the current submission to address the issue. Additionally, the figures have been removed from the manuscript text and are now provided as separate TIFF files, each labeled with its corresponding figure number. We hope all issues have been satisfactorily resolved and look forward to your further comments. Thank you, and have a pleasant day.

Attachment Submitted filename: Answer Latter L Bohorquez sign.pdf

# Editor report: Decision Letter 3

**DOI:** 10.1371/journal.pone.0358441.r007

**Authors:** Wenguo Cui

2 Sep 2026

A Detailed Method for Copper Doping of Synthetic Hydroxyapatite Thin Films via Plasma-Assisted Pulsed Laser Deposition

PONE-D-26-06266R3

Dear Dr. Bohorquez Santiago,

We’re pleased to inform you that your manuscript has been judged scientifically suitable for publication and will be formally accepted for publication once it meets all outstanding technical requirements.

Within one week, you’ll receive an e-mail detailing the required amendments. When these have been addressed, you’ll receive a formal acceptance letter and your manuscript will be scheduled for publication.

An invoice will be generated when your article is formally accepted. Please note, if your institution has a publishing partnership with PLOS and your article meets the relevant criteria, all or part of your publication costs will be covered. Please make sure your user information is up-to-date by logging into Editorial Manager at Editorial Manager® and clicking the ‘Update My Information' link at the top of the page. For questions related to billing, please contact billing support.

If your institution or institutions have a press office, please notify them about your upcoming paper to help maximize its impact. If they’ll be preparing press materials, please inform our press team as soon as possible -- no later than 48 hours after receiving the formal acceptance. Your manuscript will remain under strict press embargo until 2 pm Eastern Time on the date of publication. For more information, please contact onepress@plos.org.

Kind regards,

Wenguo Cui, Ph.D

Academic Editor

PLOS One

Additional Editor Comments (optional):

Reviewers' comments:

# Editor report: Acceptance letter

**DOI:** 10.1371/journal.pone.0358441.r008

**Authors:** Wenguo Cui

PONE-D-26-06266R3

PLOS One

Dear Dr. Bohorquez Santiago,

I'm pleased to inform you that your manuscript has been deemed suitable for publication in PLOS One. Congratulations! Your manuscript is now being handed over to our production team.

At this stage, our production department will prepare your paper for publication. This includes ensuring the following:

* All references, tables, and figures are properly cited

* All relevant supporting information is included in the manuscript submission,

* There are no issues that prevent the paper from being properly typeset

You will receive further instructions from the production team, including instructions on how to review your proof when it is ready. Please keep in mind that we are working through a large volume of accepted articles, so please give us a few days to review your paper and let you know the next and final steps.

Lastly, if your institution or institutions have a press office, please let them know about your upcoming paper now to help maximize its impact. If they'll be preparing press materials, please inform our press team within the next 48 hours. Your manuscript will remain under strict press embargo until 2 pm Eastern Time on the date of publication. For more information, please contact onepress@plos.org.

You will receive an invoice from PLOS for your publication fee after your manuscript has reached the completed accept phase. If you receive an email requesting payment before acceptance or for any other service, this may be a phishing scheme. Learn how to identify phishing emails and protect your accounts at https://explore.plos.org/phishing.

If we can help with anything else, please email us at customercare@plos.org.

Thank you for submitting your work to PLOS One and supporting open access.

Kind regards,

PLOS One Editorial Office Staff

on behalf of

Professor Wenguo Cui

Academic Editor

PLOS One
